# Supplementary material for: Astaxanthin: Past, Present, and Future
Source: Mar Drugs. 2023 Sep 28;21(10):514. doi: 10.3390/md21100514 (PMC10608541; doi:10.3390/md21100514)
Supplement: Supplementary file 1 [file marinedrugs-21-00514-s001.zip › marinedrugs-21-00514-s001/S1.pdf]

Supplementary Table S1.

Supplementary Table S1. Physical properties and main spectral data of astaxanthin

Physical property	Description
**Melting point**	182—183℃(Recrystallized from acetone-petroleum ether)<br>215—216℃(Recrystallized from pyridine)
**Solubility**	Easily soluble in CH_3Cl, CH_2Cl_2, CCl_4, DMSO, DHF, benzene and pyridine; soluble in acetone, EtOH and ether, slightly soluble in hexane; insoluble in water
**Visible absorption maxima (λ max)**	485 nm (in CH_3Cl), 480 nm （in Acetone), 472 nm (EtOH), 472 nm (MeOH) and 466 nm (Hexane)
**Circular dichroism (CD) spectrum**	Shown in Supplementary Figure S2.
**MS (Major ions in electron ionization mass spectra)**	m/z 596 $[M]^+$ (Relative intensity 20%), 580 (4%), 578 (10%), 504 (3%), 490 (3%), 106 (40%), 91 (100%)
**NMR**	Chemical shift (ppm) in $CDCl_3$ solution

^{1}H-NMR

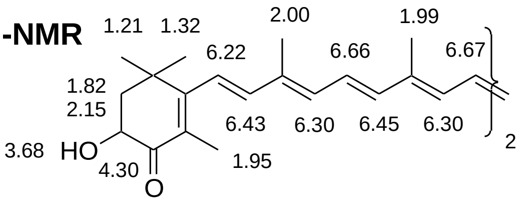

^{13}C-NMR

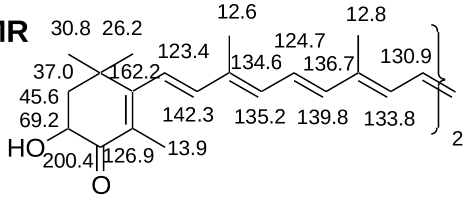

Supplementary Figure S1.

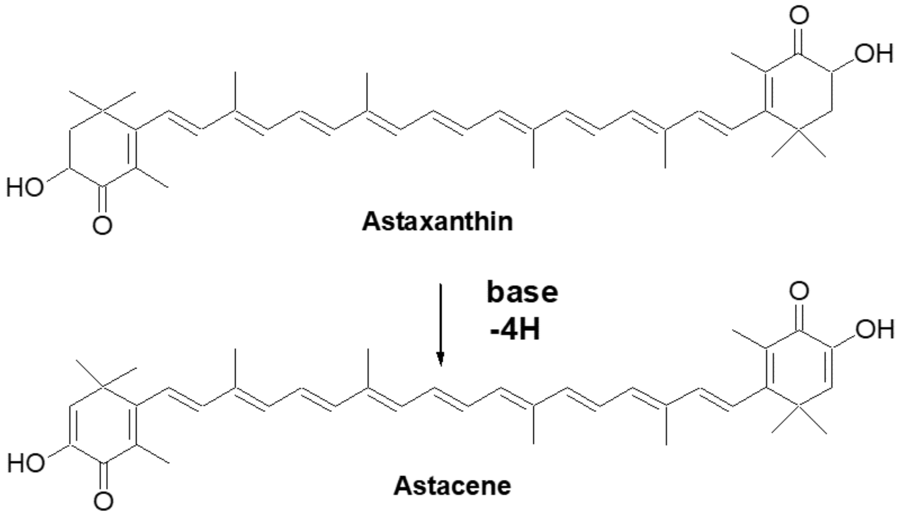

Supplementary Figure S1. Oxidation of astaxanthin to astacene in alkaline solution.

Supplementary Figure S2.

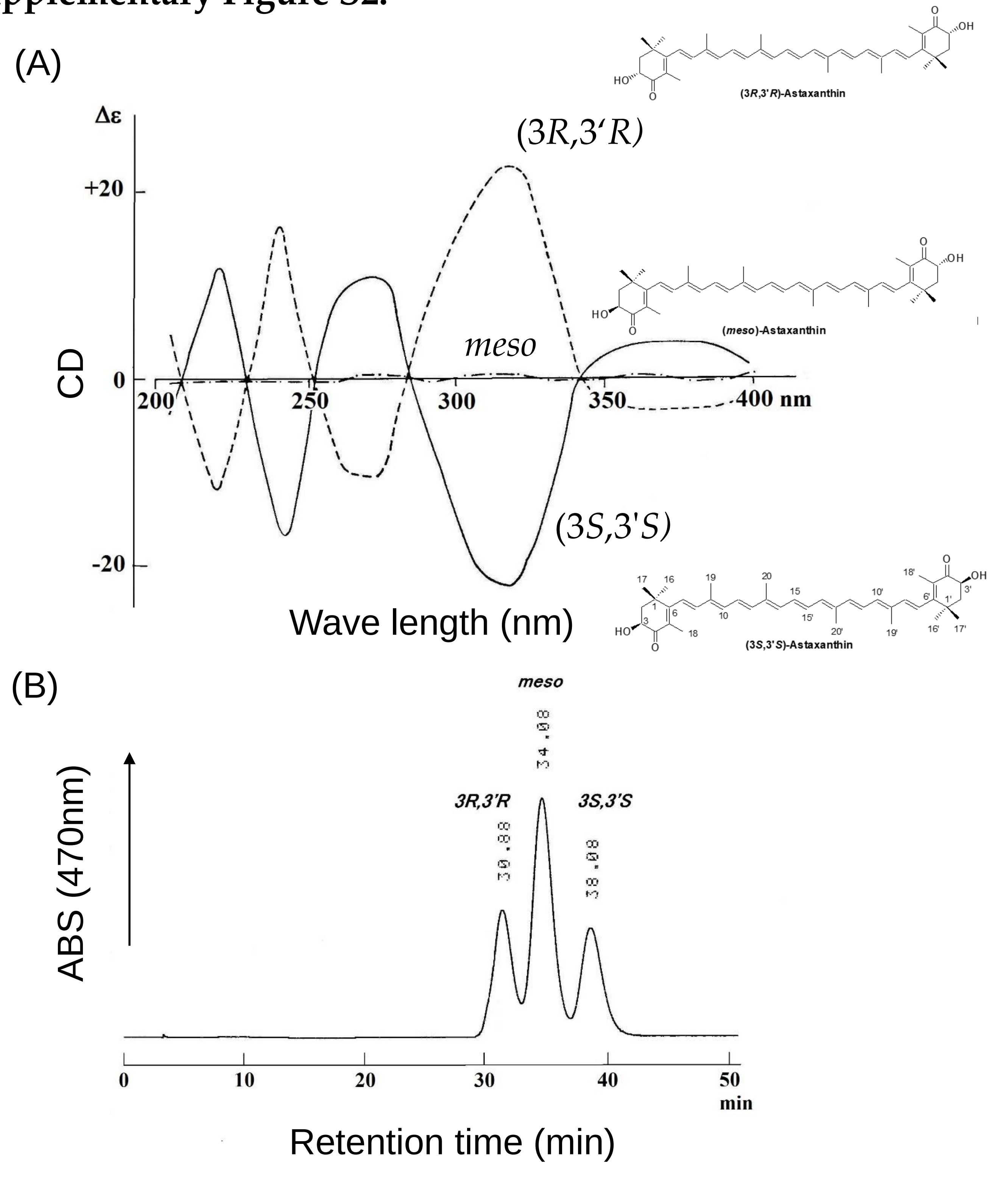

Supplementary Figure S2. Separation of optical isomers of astaxanthin (AX) . (A) (3*S*,3'*S*)-AX (—), (3*R*,3'*R*)-AX (- - -), and CD spectra of *meso*-AX (– · –) in ether (measured at room temperature). (B) Separation of AX optical isomers by HPLC using an optical isomer separation column Sumichiral OA-2000 (Sumitomo Chemical, Japan); mobile phase *n*-hexane: CH_3Cl; EtOH (48:16:0.1), flow rate 1.0 ml/min, detection by UV/VIS at 470 nm.

Supplementary Table S3.

Supplementary Table S3. Characteristics of chromatophores on the integument of poikilotherm animals*.

Chromatophore	Melanophore/ Melanocyte	Erythrophore**	Xanthophore**	Leucophore	Iridophore	Cyanophore
**Distribution**	Invertebrates to Vertebrates. *Avian and Mammals: Melanocytes.	Invertebrates to Heterothermic Vertebrates	Invertebrates to Heterothermic Vertebrates	Invertebrates to Heterothermic Vertebrates	Invertebrates to Heterothermic Vertebrates	Fishes very limited, only known 2 species of *Synchiropus*, family Callionymidae -
**Color**	Light Brown to Black	Orange to Red**	Yellow to Orange**	White	Iridescent/Silverly/ Blue/Green	Blue
**Chromatic Pattern/Chromophore**	**Pigmentary**<br><br>Melanins (eumelanin/pheomelanin)	**Pigmentary**<br><br>Carotenoids*** /Pteridines	**Pigmentary**<br><br>Carotenoids*** /Pteridines	**Structural**<br><br>Cytoplasm, Purine/Colorless Pteridines	**Structural**<br><br>Primary Guanine Platelet, Crystalline of Uric acid and Hypoxanthin	**?**<br><br>Unknown
**Spectral Properties**	Absorptive/Approximately even across the visible spectrum (300–700 nm)	Absorptive/Combination of three pigments with λmax at 440, 467 and 477 nm	Absorptive/Combination of three pigments with λmax at 440, 467 and 477 nm	Reflective, Scatter/300–700 nm (especially in the UV range)	Reflective/350–400 nm and 500–600 nm, Thin Film Interference	Unknown/Blue
**Pigment Granule (Organelle)**	Melanosome	Pterinosomes and Carotenoid Vesicles	Pterinosomes and Carotenoid Vesicles	Leucosome	Iridosome	Cyanosome
**Form of Pigment Granule**	Oval	dendritic cells	dendritic cells	dendritic cells		dendritic cells

* This table was prepared based on the ref. [1,2,3,4].
Note that information on dichromatic chromatophores was excluded from this table.
** The classification is based on their coloration, and the both are principally identical. The quantity and quality of carotenoids and pteridines present change the color of both.
***As carotenoids, xanthophores contain mainly xanthophylls such as lutein, zeaxanthin, and tunaxanthin, and red chromatophores contain mainly ketocarotenoids, including astaxanthin.

Supplementary Figure S3.

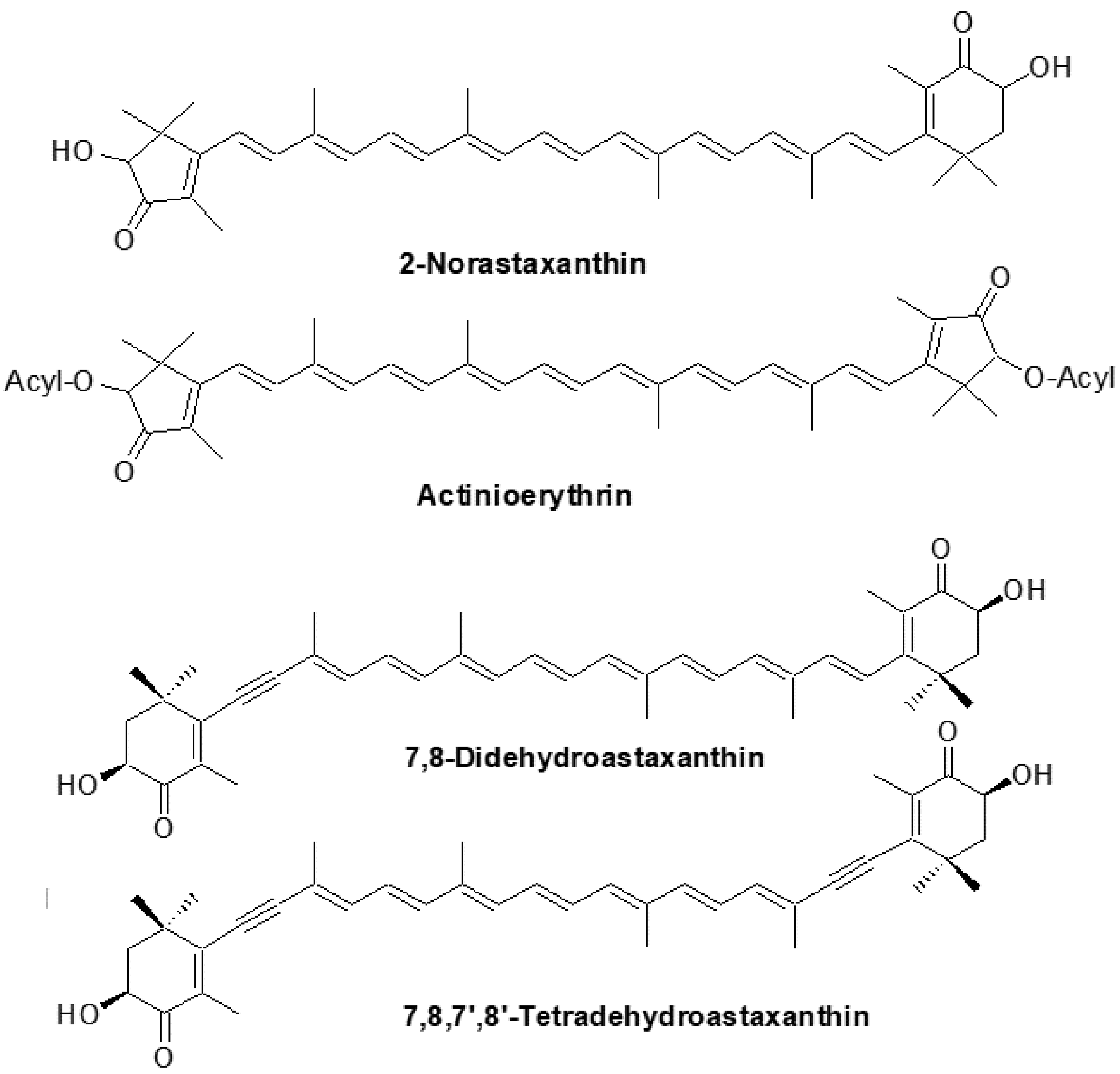

Supplementary Figure S3. Structure of astaxanthin analogues in marine invertebrates.

Supplementary Figure S4.

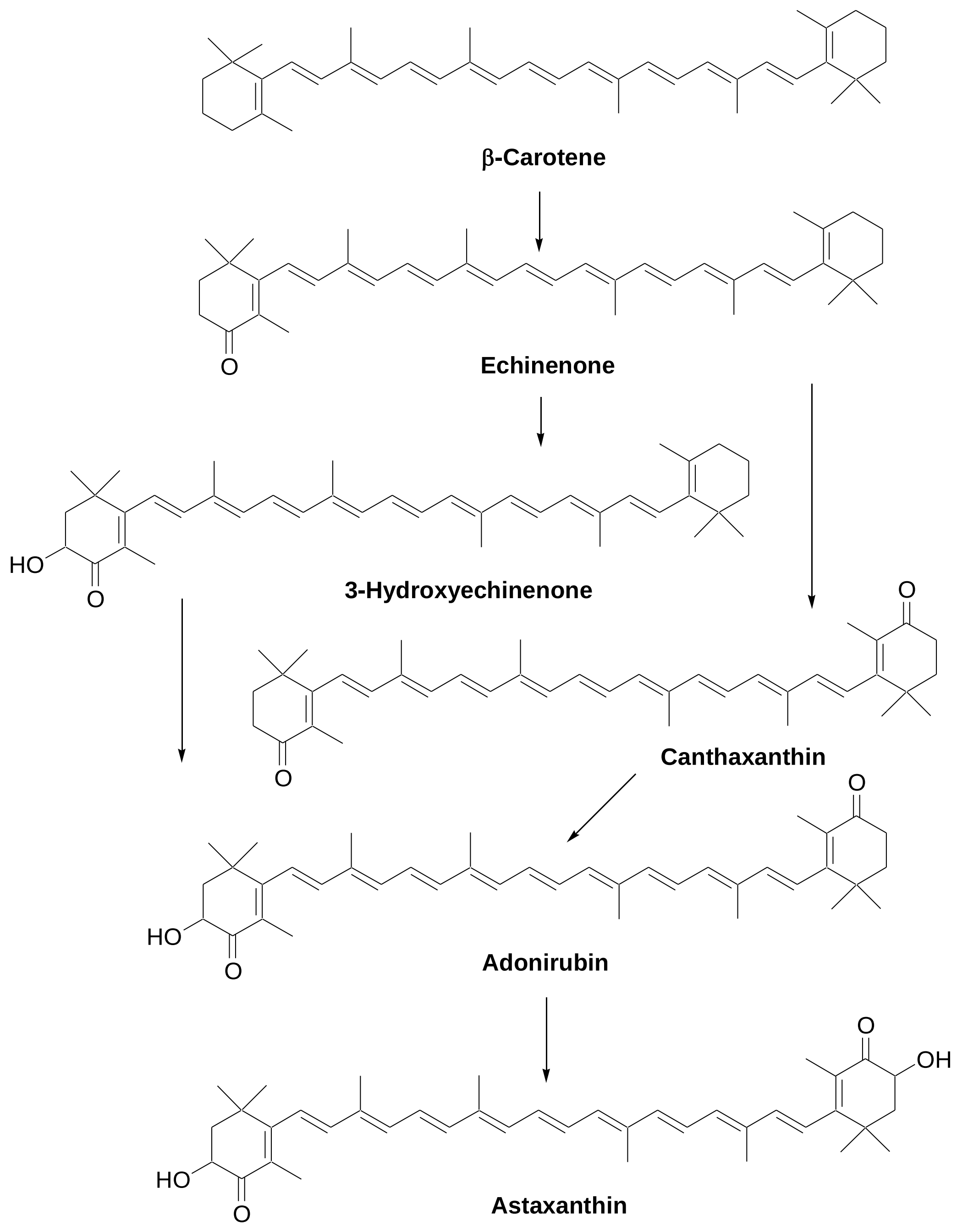

Supplementary Figure S4. oxidative bioconversion of β-carotene to astaxanthin in some animals.

Supplementary Figure S5.

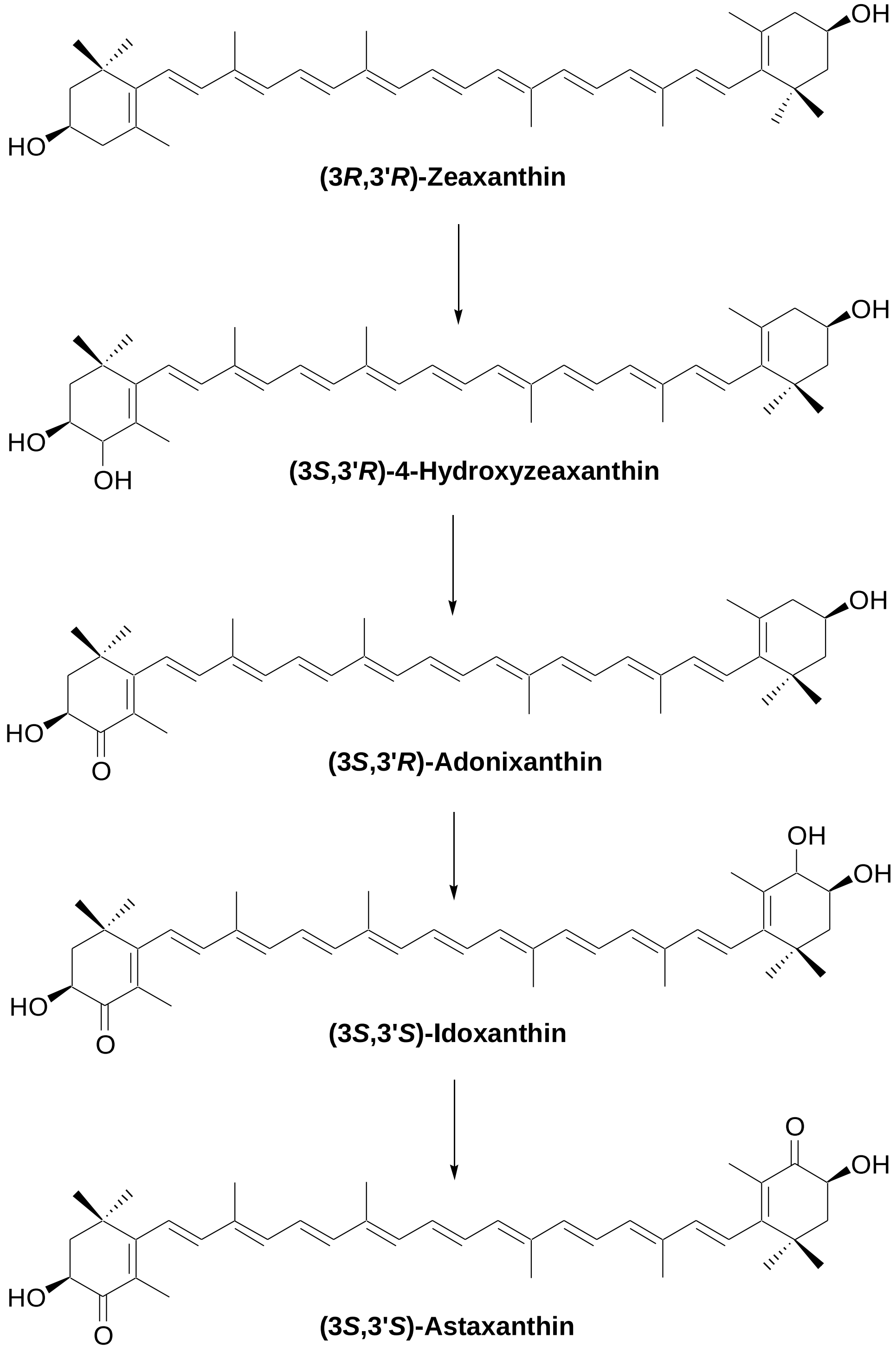

Supplementary Figure S5. Bioconversion of (3*R*,3′*R*)-zeaxanthin to astaxanthin in fishes of the family Cyprinidae.

Supplementary Figure S6.

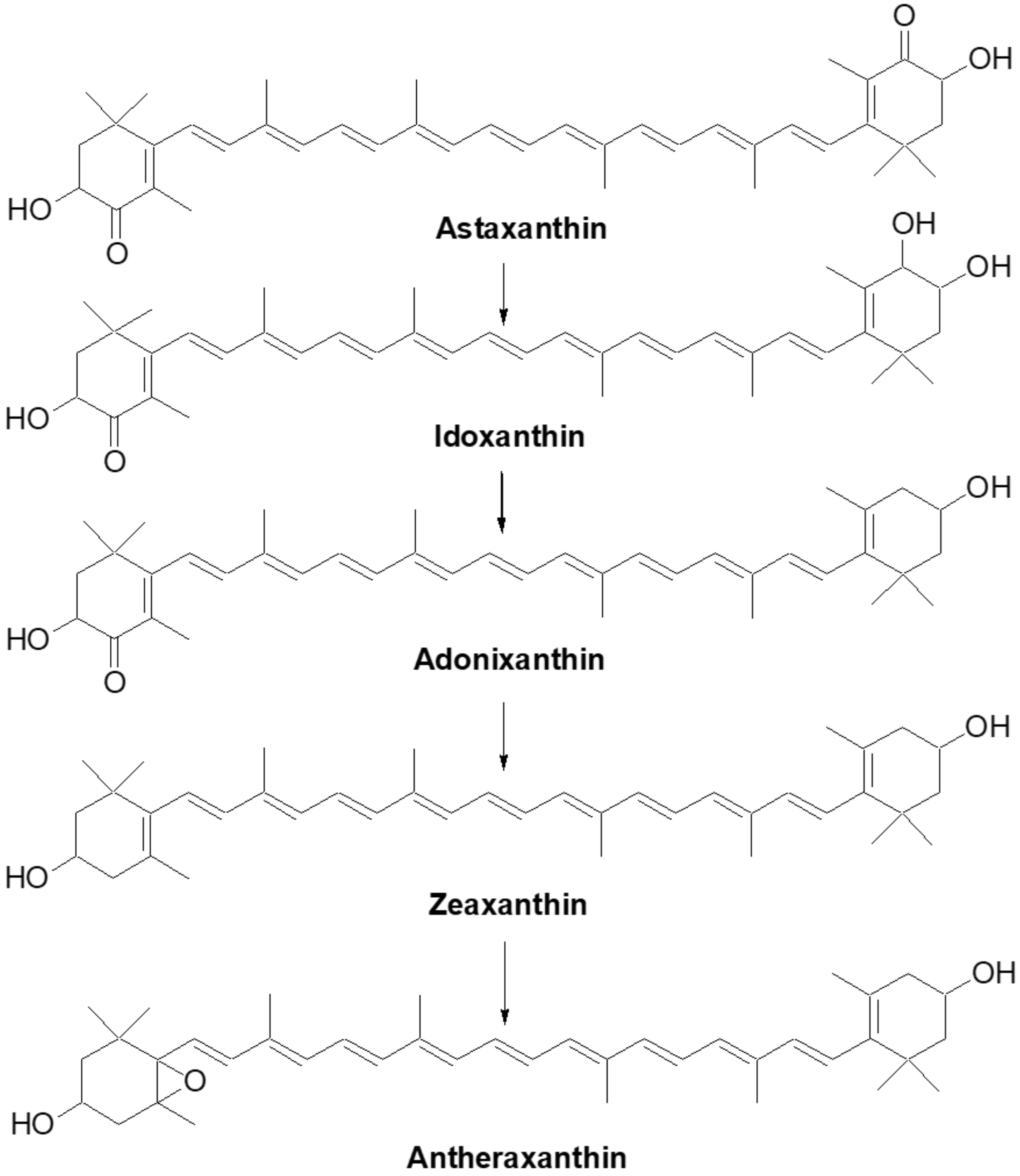

Supplementary Figure S6. Structure of astaxanthin analogues in marine invertebrates.

Supplementary Figure S7.

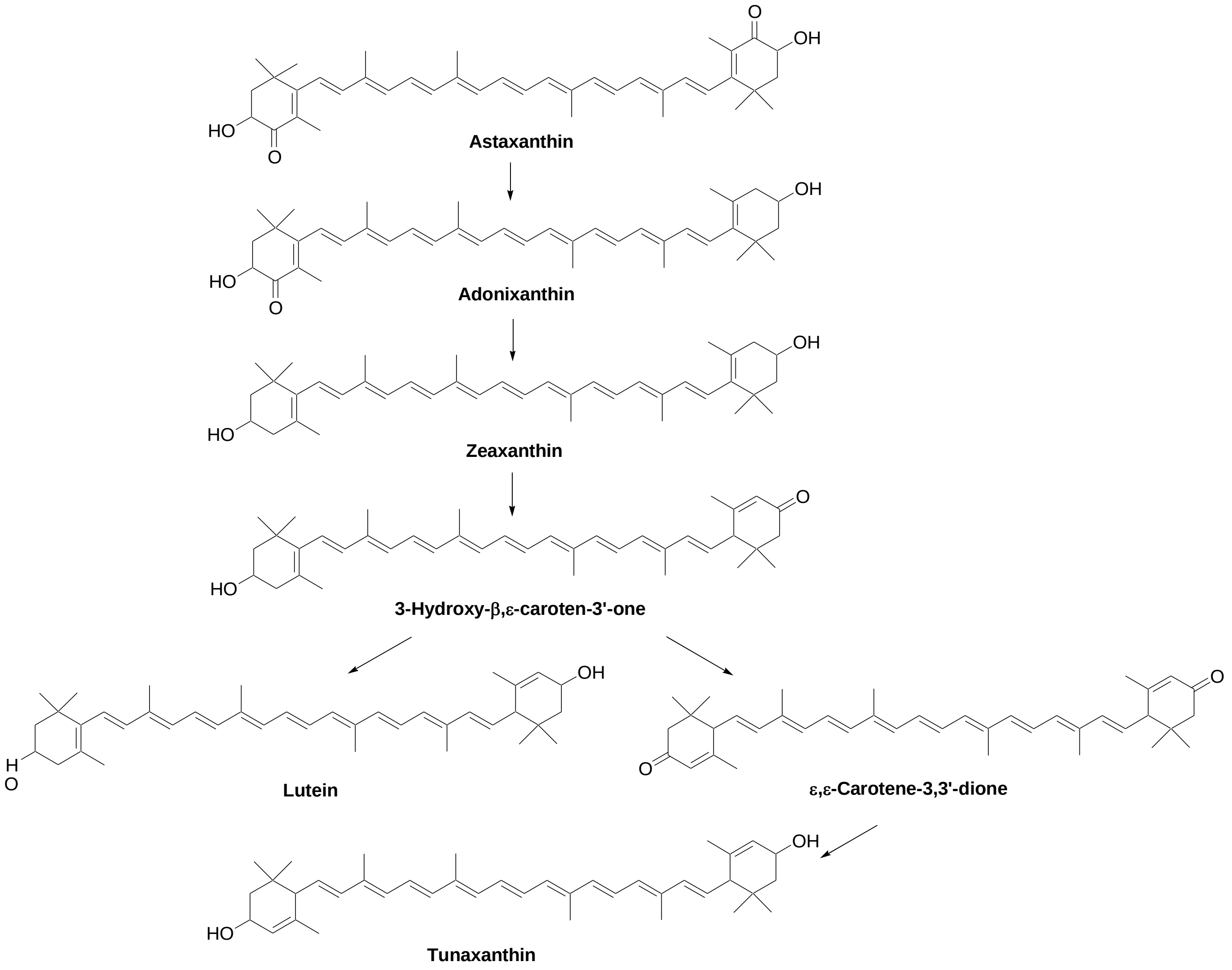

Supplementary Figure S7. Reductive metabolic pathway from astaxanthin to tunaxanthin in fishes.

References

1. Akiyama, T.; Sasaki, M.; Tkneka, Y. Body color and pattern formations in animals: pigment cell development, genes and a reaction-diffusion model. *Hiyoshi Review of Natural Science Keio University (in Japanse)* **2005**, *37*, 73-94.

2. Schartl, M.; Larue, L.; Goda, M.; Bosenberg, M.W.; Hashimoto, H.; Kelsh, R.N. What is a vertebrate pigment cell? *Pigment Cell Melanoma Res* **2016**, *29*, 8-14, doi:10.1111/pcmr.12409.

3. Kelley, J.L.; Davies, W.I.L. The Biological Mechanisms and Behavioral Functions of Opsin-Based Light Detection by the Skin. *Frontiers in Ecology and Evolution* **2016**, *4*, doi:10.3389/fevo.2016.00106.

4. Goda, M.; Fujii, R. Blue chromatophores in two species of callionymid fish. *Zoological science* **1995**, *12*, 811-813.
