# Supplementary material for: Astaxanthin: Past, Present, and Future
Source: Mar Drugs. 2023 Sep 28;21(10):514. doi: 10.3390/md21100514 (PMC10608541; doi:10.3390/md21100514)
Supplement: Supplementary file 1 [file marinedrugs-21-00514-s001.zip › marinedrugs-21-00514-s001/S2.pdf]

Supplementary Table S2. Singlet oxygen ($^1O_2$) quenching activity of common carotenoids [$k(10^9M^{-1}s^{-1})$]

**$^1O_2$ generator**			EDN			EDN		$NDPO_2$		Phenazine			EP
**Reference**			[1]			[2]		[3]	[4]	[5]		[6]	[7]
**Method**			TD*			TD*		TD*		Photosentizing/pulse laser			TD*
**Detection**			Luminescence			Luminescence		Luminescence		Luminescence			Absorbance of DPBF
**Solvent**			$CDCl_3$	$CDCl_3$/ CD_3OD (2:1)	DMF/ $CDCl_3$ (9:1)	$CDCl_3$	$CDCl_3$/ CD_3OD (2:1)	EtOH/$CHCl_3$ /D_2O (50:50:1)		Benzene	Toluene	Benzene	EtOH/$CHCl_3$/D_2O (50:50:1)
**Carotenoid**	*n**	-OH											
Dodecapreno-β-carotene	19	0	-	-	-	-	-	-	-	23	29	-	-
Decapreno-β-carotene	15	0	-	-	-	-	-	13.3	-	20	21.0	-	-
C50-Astaxanthin	15+2	2						13.0	-	-	-	-	-
**<u>Astaxanthin (AX)</u>**	**11+2**	**2**	**2.2**	**1.8**	**5.4**	**2.2**	**1.8**	**9.0**	**24**	**14**	**-**		**11.7**
**Adonirubin**	11+2	1	-	-	-	-	-	-	-	-	-	10.4	-
**Anonixanthin**	11+1	2	-	-	-	-	-	-	-	-	-	12.3	-
**Canthaxanthin**	11+2	0	2.2	1.3	2.0	-	1.2	10.2	21	-	13	-	-
**Zeaxanthin**	11	2	2.0	0.73	3.4	1.9	0.12	-	10	12	-	-	11.2
**β-Cryptoxanthin**	11	1	2.0	0.27	1.7	-	-	-	6	-	-	-	7.0

**all-*trans* β-Carotene**	11	0	2.2	0.28	1.1	2.2	0.049	8.4	14	13	14	-	10.8
**9-*cis* β-Carotene**	11	0	-	-	-	-	-	-	-	11	9	-	-
**15-*cis* β-Carotene**	11	0	-	-	-	-	-	-	-	11	12	-	-
**Lycopene**	11	0	3.0	1.4	3.4	-	-	8.8	31	17	-	-	14.0
**Capsanthin**	10+1	2						-		-	-	-	12.1
**Lutein**	10	2	0.61	0.26	2.1	0.8	-	-	8	16	-	6.64	8.1
***α*-Carotene**	10	0	0.66	0.23	0.93	-	-	-	19	-	8.2	-	10.0
**Capsorbin**	9+2	2	-	-	-	-	-	-	-	-	-	12	-
**Fucoxanthin**	9+1	2	0.29	0.075	0.97	-	0.005	-	-	-	-	-	-
**Tunaxanthin**	9	2	-	-	-	0.15	-	-	-	-	-	-	-
**Violaxanthin**	9	2	-	-	-	-	-	-	-	16	-	-	-
Septapreno-β-Carotene	9	0	-	-	-	-	-	-	-	-	-	1.38	-
Bixin	9(+2)	0	-	-	-	-	-	-	-	9.2	-	-	-
7,7′-Dihydro-β-Carotene	8	0	-	-	-	-	-	-	-	-	-	0.3	-
***α*-Tocopherol**	N/A	N/A	0.02	0.0039	0.049	-	-	-	0.28	-	-	-	0.13

*Numbers were given as number of conjugation length of C=C(+C=O) of each carotenoid.

EDN, 1,4-dimethylnapthalene endoperoxide; NDPO2, of 3,3'-(1,4-naphthylene)dipropionate endoperoxide; EP, 1-methylnaphthalene-4-

propionate endoperoxide; DPBF, 1,3-diphenylisobenzofuran; DMF, N,N-dimethylformamide.

References

1. Nishida, Y.; Yamashita, E.; Miki, W. Quenching activities of common hydrophilic and lipophilic antioxidants against singlet oxygen using chemiluminescence detection system. *Carotenoid Science* **2007**, *11*, 16-20.
2. Shimidzu, N.; Goto, M.; Miki, W. Carotenoids as Singlet Oxygen Quenchers in Marine Organisms. *Fisheries science* **1996**, *62*, 134-137, doi:10.2331/fishsci.62.134.
3. Baltschun, D.; Beutner, S.; Briviba, K.; Martin, H.-D.; Paust, J.; Peters, M.; Röver, S.; Sies, H.; Stahl, W.; Steigel, A.; et al. Singlet Oxygen Quenching Abilities of Carotenoids. *Liebigs Annalen* **1997**, *1997*, 1887-1893, doi:10.1002/jlac.199719970913.
4. Di Mascio, P.; Kaiser, S.; Sies, H. Lycopene as the most efficient biological carotenoid singlet oxygen quencher. *Arch Biochem Biophys* **1989**, *274*, 532-538, doi:10.1016/0003-9861(89)90467-0.
5. Conn, P.F.; Schalch, W.; Truscott, T.G. The singlet oxygen and carotenoid interaction. *J Photochem Photobiol B* **1991**, *11*, 41-47, doi:10.1016/1011-1344(91)80266-k.
6. Edge, R.; Truscott, T.G.; Landrum, J.T.e. *Carotenoids: Physical, Chemical, and Biological Functions and Properties*; Taylor & Francis: 2009.
7. Mukai, K. Antioxidant Activity of Foods: Development of Singlet Oxygen Absorption Capacity (SOAC) Assay Method. *J Nutr Sci Vitaminol (Tokyo)* **2019**, *65*, 285-302, doi:10.3177/jnsv.65.285.
