# Supplementary material for: Astaxanthin: Past, Present, and Future
Source: Mar Drugs. 2023 Sep 28;21(10):514. doi: 10.3390/md21100514 (PMC10608541; doi:10.3390/md21100514)
Supplement: Supplementary file 1 [file marinedrugs-21-00514-s001.zip › marinedrugs-21-00514-s001/S3.pdf]

Supplementary Table S4. Astaxanthin: Update on efficacy from human clinical studies.

Author/year/ reference	Study design	Subjects	Dose#,##	Duration	Outcome†	Description
Barker G.A. *et al.* 2023 [1]	Double-blind, placebo-controlled prospective study	19 Resistance-trained males	0, 12 mg/day	4 weeks	✓ Sports performance (Muscle damage/ recovery after eccentric exercise)	Significantly decreased in delayed onset muscle soreness (SORE score (*p*=0.02)) for the AX group post-supplementation compared to pre-supplementation test. No effect in Placebo. Significantly decreased in VAS (visual analog scale; *p*=0.01) for the AX group post-supplementation compared to pre-supplementation test, whereas these no effect in the Placebo group. No effect on performance. <u>*No safety concerns were identified in this study.*</u>
Ciaraldi T.P. *et al.* 2023 [2]	Randomized, double-blind, placebo-controlled prospective study	34 Obese subjects with prediabetes and dyslipidaemia	0, 12 mg/day	24 weeks	✓ Cardiovascular health ✓ Metabolic syndrome ✓ Oxidative stress	After 24 weeks, significantly decreased low-density lipoprotein (−0.33 ± 0.11 mM) and total cholesterol (Chol, −0.30 ± 0.14 mM) (both p<0.05) in AX group. Reduced levels of the cardiovascular disease (CVD) risk markers fibrinogen (−473 ± 210 ng/mL), L-selectin (−0.08 ± 0.03 ng/mL) and fetuin-A (−10.3 ± 3.6 ng/mL) (all *p*<0.05) in AX group. The trend of improvement in insulin action was also observed in insulin-stimulated whole-body glucose treatment (+0.52 ± 0.37 mg/m2/min, *p*=0.078), as well as in fasting [insulin] (-5.6 ± 8.4 pM, *p*=0.097) and HOMA2-IR (-0.31 ± 0.16, *p*=0.060). No consistent significant differences from baseline were observed in any of these results in the placebo group. <u>*No safety concerns were identified in this study.*</u>
Heidari M. *et al.* 2023 [3]	Randomized, double-blind, placebo-controlled prospective study	44 CAD patients (40–65 yrs.), angiographic evidence of 50% stenosis in at least one of the major coronary arteries, 25 < BMI <35	0, 12 mg/day	8 weeks	✓ Cardiovascular health ✓ Metabolic syndrome ✓ Oxidative stress	12 mg AX or placebo (microcrystalline cellulose) groups along with low calorie diet, for a period of 8 weeks. Significant reduced in total cholesterol (−14.95 ± 33.57 mg/dl, p<0.05) and LDL-C (−14.64 ± 28.27 mg/dl, p<0.05) in AX group with coronary artery disease (CAD). However, TG and HDL-C levels could not be affected through AX. Did not change "serum" levels of Sirtuin1 and TNF-*α*, Body composition, glycemic indices. (Comments: BMI 25-27 mild obesity, non-insulin resistant with normoglycemia, normal TC, slightly elevated TG, and very low HDL-Chol. The effect on body composition seems to be more influenced by the low calorie diet. The clinical significance of the serum free form of Sirt-1 remains unclear.) <u>*No safety concerns were identified in this study.*</u>
Jabarpour M. *et al.* 2023 [4]	Randomized, placebo-controlled prospective study	53 Patients with polycystic ovary syndrome (PCOS)	0, 6 mg/ twice a day (0,12 mg/day)	60 days	✓ Women's health ✓ Oxidative Stress (PCOS) ✓ ER stress ✓ Infertility ✓ Assisted reproductive technology (ART)	<u>ER stress (Granulosa cells)</u>: After the intervention, AX treatment reduced in the mRNA expression levels of 78-kDa glucose-regulated protein (GRP78), CCAAT/enhancer-binding protein homologous protein (CHOP), and X-box-binding protein 1 compared to the placebo group, but activating transcription factor 6 (ATF6) was not statistically significant. However, AX significantly increased the ATF4 expression level. GRP78 and CHOP protein levels represented a considerable decrease in the treatment group after the intervention. <u>Antioxidant markers</u>: Increased levels of TAC in follicular fluid. ART outocomes: higher rates of high-quality oocytes, high-quality embryo, and oocyte maturity in AX group. (the oocyte number, fertilization rate, and fertility rate; N.S.) <u>*No safety concerns were identified in this study.*</u>

Nieman, D.C. *et al.* 2023 [5]	Randomized, double-blind, placebo-controlled crossover study	18 healthy subjects (capable of running 2.25 h on laboratory treadmills at 70% maximal oxygen consumption rate (VO_{2max}))	0, 8 mg/day	4 weeks	✓ Sports performance<br>✓ Immunity	The running bout for 2.25 h induced significant muscle soreness, muscle damage, and inflammation; AX supplementation had no effect on exercise-induced muscle soreness, muscle damage, and increases in 6 plasma cytokines and 42 oxylipins. However AX supplementation inhibited exercise-induced decreases in 82 plasma proteins (at 24 hours post-recovery). Most of these proteins were involved in immune-related functions. Twenty plasma immunoglobulins were identified that differed significantly between the AX and placebo group; plasma levels of IgM were significantly reduced after exercise but recovered in the AX trial but not in the placebo trial after a 24-hour recovery period after exercise.<br>*No safety concerns were identified in this study.*
Rostami S. *et al.* 2023 [6]	Randomized, triple-blind, placebo-controlled prospective study	50 Patients of endometriosis (stage III/ IV)	0, 6 mg/day	12 weeks	✓ Women's health<br>✓ Oxidative Stress (endometriosis)<br>✓ Infertility<br>✓ Assisted reproductive technology (ART)	Antioxidant markers: Increased serum levels of total antioxidant capacity (TAC, p=0.004) and superoxide dismutase (SOD, 13.458 ± 7.276 vs. 9.040 ± 5.155; p=0.010) were observed in the AX intervention group after therapy. In addition, serum Malondialdehyde (MDA, p=0.031) decreased significantly after AX treatment. Inflammation markers: Serum IL-1β (p=0.000), IL-6 (p=0.024) and TNF-α (p=0.038) showed significantly lower levels after AX treatment. ART outcomes: AX supplementation resulted in a significantly improved number of oocytes retrieved (p=0.043), mature (MII) oocytes (p=0.041), and improved quality embryos (p = 0.024).<br>*No safety concerns were identified in this study.*
Saeidi A. *et al.* 2023 [7]	Randomized placebo-controlled prospective study	Obese subjects; 15 control group (CG), 15 supplement group (SG), 15 training group (TG), 15 training plus supplement group (TSG).<br><br>BMI: 33.6 ± 1.4	0, 20 mg/day	12 weeks	✓ Cardiovascular health<br>✓ Metabolic syndrome<br>✓ Energy metabolisms	Intervention: AX with/without high-intensity functional training.<br>BW,% of Fat, BMI, Fat-Free Mass: After 12 weeks, there was significant improvement in SG, TG, and TSG, but not in CG (p<0.05). $VO_{2\ peak}$: Increases after 12 weeks of exercise were significant in the TG (p = 0.0001) and TSG (p =0.0001) but not in the CG (p=0.32) and SG (p=0.21). Lipid profile (HDL, LDL, Total Cholesterol(Chol.), and Triglycerides(TG)): After 12 weeks, there was significant improvement in SG, TG, and TSG, but not in CG (p<0.05). Metabolic Factors (Insulin, glucose, HOMA-IR): Glucose and Insulin levels decreased significantly in the SG (p <0.001), TG (p <0.001), and TSG (p <0.001), but not significantly change in the CG (p>0.05), and decreased HOMA-IR following 12 weeks of training in the SG (p = 0.0001), TG (p= 0.0001), and TSG (p = 0.0001), while the difference in HOMA-IR in the CG was not significant (p=0.17). Adipokines and Growth Differentiation Factors: [Cq1/TNF-related protein 9 and 2 (CTRP9 and CTRP2) levels, and growth differentiation factors 8 and 15 (GDF8 and GDF15)] were measured. There were significant differences for all indicators between the groups (p<0.05).<br>*No safety concerns were identified in this study.*

Sekikawa T. *et al.* 2023 [8]	Randomized, double-blind, placebo-controlled prospective study	59 healthy subjects with VDT operation (Mean 39 yrs.)	0, 9 mg/day	6 weeks	✓ Eye health (eye fatigue)	<u>Visual acuity</u>: In participants ≥40 yrs, higher protective effect of AX on corrected visual acuity of the dominant eye after visual display terminal (VDT) work at 6 weeks after intake in the AX group vs the control group (p<0.05). In <40 yrs., no significant difference between the AX and control groups. <u>Functional visual acuity and pupil constriction rate</u>: No significant difference between the AX and control groups<br>*<u>No safety concerns were identified in this study.</u>*
Yoshida K. *et al.* 2023 [9]	Randomized, double-blind, placebo-controlled prospective study	57 healthy subjects	0, 6 mg/day	8 weeks	✓ Eye health (eye–hand coordination)	*Active group: 10 mg lutein, 2 mg zeaxanthin and 6 mg of AX.* Significantly improved eye–hand coordination after visual display terminal (VDT) operation at 8 weeks in the active group. No clear improvement in the effect of the supplementation on smooth-pursuit eye movements. The active group also showed a significant increase in macular pigment optical density (MPOD) levels.<br>*<u>No safety concerns were identified in this study.</u>*
Waldman H.S. *et al.* 2023 [10]	Randomized, double-blind, placebo-controlled crossover study	Resistance-trained males (Mean 23.4 yrs.)	0, 12 mg/day	4 weeks	✓ Sports performance<br>✓ Energy metabolisms	AX supplementation had no statistical effect on markers of substrate metabolism, Wingate variables, or markers of muscle damage, inflammation, or delayed-onset muscle soreness during the graded exercise test (GXT) compared to placebo (p>0.05). However, 4 weeks of AX supplementation significantly reduced oxygen consumption during the final phase of the GXT compared to placebo (12%, p = 0.02), reduced systolic blood pressure (approximately 7%, p=0.04), and significantly reduced baseline insulin levels (*c.a.* 24%, p=0.05).<br>*<u>No safety concerns were identified in this study.</u>*
Wika A.A. *et al.* 2023 [11]	Randomized, double-blind, placebo-controlled prospective study	19 obese subjects (Mean, age: 27.5 yrs; BF%: 37.9; BMI: 33.4 kg/m2; VO_{2peak}: 25.9 ml·kg−1·min^{-1})	0, 12 mg/day	4 weeks	✓ Cardiovascular health<br>✓ Metabolic syndrome<br>✓ Energy metabolisms	Subjects performed a graded exercise test on a cycling ergometer and were measured for changes in glucose and lactate levels, fat and carbohydrate (CHO) oxidation rates, heart rate, and rating of perceived exertion (RPE).<br>Although there were no changes in fat oxidation rate, blood lactate or glucose, or RPE (all p>0.05), a significant decrease in CHO oxidation rate was observed in the AX group only, before and after supplementation. In addition, in the AX group, decreased heart rate by 7% during the graded exercise stress test.<br>*<u>No safety concerns were identified in this study.</u>*
D'Aloisio R. *et al.*, 2022 [12]	Retrospective study	15 AMD patients treated with daily oral nutritional supplement with AX.<br>13 AMD patients treated w/o. daily oral nutritional supplement (control)	0, 12 mg/day*	6 months	✓ Eye health (AMD) (Microcirculatory flow)	*Nutritional supplement: 100mg lutein, 80 mg bromelain, 120 mg VC, 30 mg VE, 400 µg folic acid, Zn, Cu, 1000 IU D3 vitamin and 12 mg AX (Astazin 10).*<br>Statistically significant difference in choriocapillary vessel density (CCVD) values between cases and controls at baseline (p < 0.001) and at follow-up (p < 0.001); choroidal thickness measurements were statistically significant between cases and controls (p = 0.002) and in cases at follow-up (p < 0.001).<br>*<u>No safety concerns were identified in this study.</u>*

Dede G *et al.* 2022 [13]	N/A	30 Semen samples from normozoospermic individuals	0, 50, 100, 500 μM	N/A	✓ Infertility<br>✓ Assisted reproductive technology (ART)	Purpose: the protective efficacy of AX against the damage that occurs during sperm cryopreservation. The loss of motility due to cryopreservation of sperm was highest in the control group, and the least loss of motility was seen in the 100 μM AX group. Chromatin condensation of spermatozoa showed that the number of condensed spermatozoa was higher in the 100 μM AX group than in the other groups.<br>(*in vitro* study)
Ghantabpour T. *et al.* 2022 [14]	N/A	The first phase; 10 semen samples from healthy men,<br>the second phase; 25 semen samples from healthy men	0, 0.5, 1, 2 μM	N/A	✓ Infertility<br>✓ Assisted reproductive technology (ART)	Supplementation of sperm freezing medium with 1 μM AX was found to improve all parameters of sperm motility and viability ($p \leq 0.05$). In addition, reduced levels of ROS parameters (intracellular hydrogen peroxide and superoxide) compared to the control group ($p \leq 0.05$). AX also significantly reduced phosphatidylserine exogenous levels ($p \leq 0.05$) and lipid peroxidation ($p \leq 0.05$) after the freeze-thaw process.<br>(*in vitro* study)
Gharaei R. *et al.* 2022 [15]	Randomized, double-blind, placebo-controlled prospective study	40 Patients with polycystic ovary syndrome (PCOS)	0, 8 mg/day	40 days	✓ Oxidative Stress,(PCOS)<br>✓ Infertility<br>✓ Assisted reproductive technology (ART)	AX supplementation resulted in significantly higher serum Catalase and TAC levels in the AX group compared to the placebo group. However, there were no significant differences in serum MDA and SOD levels between groups. The expression of antioxidant genes such as Nrf2, HO-1, and NQ-1 was significantly increased in the granulosa cells (GC) of the AX group..ART: MII oocyte and high quality embryo rates were significantly increased in the AX group compared to the placebo group. There were no significant differences in chemical and clinical pregnancy rates between groups.<br>*No safety concerns were identified in this study.*
Rad NR. *et al.* 2022 [16]	Randomized, double-blind, placebo-controlled prospective study	50 Type 2 Diabetes Mellitus (T2DM) patients receiving metformin	0, 10 mg/day	12 weeks	✓ Cardiovascular health<br>✓ Metabolic syndrome<br>✓ Oxidative stress (T2DM)	*Investigation of additive synergistic effects on metformin (1000-2000 mg/day).*<br>T2DM: After the intervention, while FBS, HbA1c (probably maintained in the high-normal range by metformin even at baseline) and Systolic blood pressure (normal range) tended to decrease in both groups, blood lipids (within normal range) remained unchanged. Significantly reduced FBS in the AX group rather than the placebo group.<br>Oxidative stress: Significantly increased blood TAC levels at the end of the intervention only in the AX-treated group, while MDA remained unchanged. Similarly, increased SOD and catalase activity in blood and increased Nrf2 protein in PBMCs were observed at the end of the intervention only in the AX-treated group.<br>*No safety concerns were identified in this study.*

Stonehouse W *et al.* 2022 [17]	Randomized, double-blind, placebo-controlled prospective study	235 Healthy adults (40–65 yrs, BMI >18.5 to <35) w/.clinically diagnosed with mild to moderate knee OA	0, 0.35 mg/day (in krill oil)	6 months	✓	Joint health	Knee pain scores (Western Ontario and McMaster Universities Osteoarthritis Index: WOMAC, numeric scale) improved in both groups, with greater improvement with krill oil than placebo. Knee stiffness and physical function also showed greater improvement with krill oil than placebo. NSAID usage, serum lipids profile, inflammatory markers, and safety markers did not differ between groups. *No safety concerns were identified in this study.*
McAllister MJ. *et al.* 2022 [18]	Randomized, double-blind, placebo-controlled crossover study	14 healthy subjects (23 ± 2 yrs)	0, 6 mg/day	4 weeks	✓<br>✓<br>✓	Oxidative Stress, (exercise)<br>Sports performance<br>Energy metabolisms	A graded exercise test was performed after each treatment to measure substrate utilization during exercise at increased intensity. Glutathione was approximately 7% higher after AX treatment compared to placebo (p=0.02, d=0.48). Plasma hydrogen peroxide and malondialdehyde (MDA) did not differ between treatments (p>0.05). Although not statistically significant (p=0.45), highly oxidized protein products were reduced by approximately 28%. In the graded exercise stress test, mean fat oxidation rates did not differ between treatments (p>0.05); however, fat oxidation was reduced from 50 to 120 W (p<0.001) and from 85 to 120 W (p=0.004) in both conditions. *No safety concerns were identified in this study.*
Tian L. *et al.* 2022 [19]	Open-labeled, prospective study	60 middle-aged and elderly patients with mild-to-moderate dry eye disease (DED)	6 mg/ twice a day (12mg/day)	30 days	✓	Eye health (DED)	Significantly improved (p< 0.05) to varying degrees after treatment compared to pre-treatment for the ocular surface disease index (OSDI) score, non-invasive tear break-up time (NIBUT), fluorescein break-up time (BUT), corneal fluorescein staining (CFS) score, eyelid margin signs, MG expressivity, mibum quality, and blink frequency, but no differences were found for tear meniscus height, Schirmer I test, conjunctival hyperemia, tear fluid lipid layer thickness, meibum quality, meibomian gland dropout (MGDR), incomplete blink rate, Visual acuity (VA), intraocular pressure (IOP) *No safety concerns were identified in this study.*
Yamazaki I. *et al,* 2022 [20]	Randomized, double-blind, placebo-controlled prospective study	257 subjects feeling fatigue with aging and on daily (Av. 44-45 yrs.)	0, 6 mg/day*	8 weeks	✓	Fatigues	*Combination with sesamins (sesame lignans).* Visual Analogue Scale (VAS) scores of the fatigue feeling at 4 weeks in the test food groups compared to the placebo food groups (p < 0.05), and these scores tended to be lower in the test food groups than the placebo food groups at 8 weeks (p < 0.1) Subgroup analysis: (A) There was a trend toward greater improvement in the group with a greater VAS value at screening test than in the group with a smaller VAS value for fatigue at screening test. (B)The frequency of exercise at screening was divided into two groups: "most/several times a month" and "at least once a week/every day." The "at least once a week/every day" group, which exercised more frequently, showed more improvement in the VAS. *No safety concerns were identified in this study.* [Article in Japanese]

Brown, R.D. *et al.* 2021 [21]	Randomized, double-blind, placebo-controlled crossover study	12 recreationally trained male cyclists (27.5 ± 5.7 years, VO_{2peak}: 56.5 ± 5.5 $mL \cdot kg^{-1} \cdot min^{-1}$, W_{max}: 346.8 ± 38.4 W)	0, 12 mg/day	7 days	✓ Sports performance<br>✓ Energy metabolisms	Completion time of the 40-km cycling time trial improved by 1.2 ± 1.7% with AX supplementation, from 70.76 ± 3.93 min in the placebo condition to 69.90 ± 3.78 min in the AX condition (mean improvement time = 51 ± 71 s, *p*=0.029, g=0.21). Whole body fat oxidation rate was also greater in the AX group between 39-40 km (+0.09 ± 0.13 g · min-1, *p*=0.044, g=0.52) and respiratory exchange ratio was lower (-0.03 ± 0.04, *p*=0.024, g=0.60).<br>*No safety concerns were identified in this study.*
Hashimoto H. *et al.* 2021 [22]	Open-labeled, prospective study	35 subjects who underwent bilateral cataract surgery (intraocular lens implantation) (Mean *c.a* 71 yrs.)	6 mg/day	2 weeks	✓ Eye health<br>✓ Oxidative stress (Cataract surgery)	Analyzed the antioxidant effect of AX in relationship to age. None of the parameters were correlated with age before AX intake, but only total hydroperoxide values were significantly correlated after AX intake (r = 0.4, p<0.05). Total hydroperoxide levels were similar in younger and older age groups (<70 vs ≥70 years) before AX intake, but significantly decreased in younger age groups after intake (-0.21 ± 0.18 vs -0.05 ± 0.31, p<0.05), resulting in a significant difference (p < 0.05). Thus, the previously observed decrease in mean total hydroperoxide levels after AX intake was likely due to a greater response in the younger age group.<br>Analysis associated with the study [23].<br>*No safety concerns were identified in this study.*
Ishiwata S. *et al.* 2021 [24]	Open-labeled, prospective study	17 patients with systolic heart failure	12 mg/day*	3 months	✓ Cardiovascular health<br>✓ Oxidative stress (Heart failure)	After 3 months of AX supplementation, increased the "Specific Activity Scale" score from a median of 4.5 (interquartile range, 2.0) to 6.5 (interquartile range, 1.1) metabolic equivalents (*p*=0.001), and the physical and mental component summary scores increased from 46.1±9.2 to 50.8±6.8 (*p*=0.015) and 48.9±9.1 to 53.8±4.8 (*p*=0.022), respectively. There was a linear relationship between baseline heart rate or mental component summary score and rate of change in the "Specific Activity Scale" score (*r*=0.523, *p*=0.031, *r*=-0.505, *p*=0.039, respectively). Furthermore, there was a direct relationship between ischemic etiology and the rate of change in the physical component summary score (r=0.483, P=0.049, respectively). There was also a linear relationship between the rate of change in the "Specific Activity Scale" score and the rate of change in the mental component summary score (*r*=0.595, *p*=0.012).<br>*No safety concerns were identified in this study.*
Kawamura A. *et al.* 2021 [25]	Randomized open-labeled, prospective study	26 healthy male subject (22.3 ± 0.3 yrs.)	N/A (1mg AX/100g salmon)*	10 weeks	✓ Oxidative Stress, (exercise)<br>✓ Sports performance<br>✓ Musculoskeletal function	The skeletal muscle mass was higher after training than before training in both control and intervention groups (*p*<0.05). Increased maximal voluntary contraction after training in the intervention group (*p*<0.05), but not significantly increased in the control group. Higher resting oxygen consumption after training in the intervention group only (*p*<0.05). Serum carbonylated protein level as an oxidative stress marker tended to be lower immediately after exercise than before exercise in the intervention group only (*p*=0.056).<br>*No safety concerns were identified in this study.*

Kizawa Y. *et al.* 2021 [26]	Randomized double-blind, placebo-controlled, prospective study	40 healthy subjects with VDT operation	0, 6 mg/day*	6 weeks	✓ Eye health (eyestrain)	*Intervention (active group): 72 mg anthocyanin from blueberry (bilberry) extract, 10 mg lutein and 6 mg AX.* After 6 weeks, significant improved in the active group compared to the placebo group in the average percentage of pupillary response in both eyes and in the dominant eye before and after operating the visual display terminal. In addition, the scores for "A sensation of trouble in focusing the eyes" and "Difficulty in seeing objects in one's hand and nearby, or fine print" were significantly improved in the active group compared to the placebo group before and after ingestion. . No statistically significant improvements were observed in tear degradation time, visual acuity, Schirmer test value, macular pigment optical density level, or muscle hardness. *No safety concerns were identified in this study.*
Liu S.Z. *et al.* 2021 [27]	Randomized, double-blind, placebo-controlled, prospective study	42 elderly subjects (65-82 yrs.)	0, 12 mg/day*	12 weeks	✓ Oxidative Stress, (elderly subjects) ✓ Musculoskeletal function ✓ Energy metabolisms	*Intervention: 6mg Zn, 10mg tocotrienol and 12mg AX.* In endurance training (ET), specific muscular endurance was improved only in the AX group (Pre 353 ± 26 vs. Post 472 ± 41) and submaximal graded exercise test duration was improved in both groups (Placebo 40.8 ± 9.1% vs. AX 41.1 ± 6.3%). The increase in fat oxidation at low intensity after ET was greater in AX (Placebo 0.23±0.15g vs AX 0.76±0.18g), and was associated with reduced carbohydrate oxidation and improved exercise efficiency in men but not in women. Analysis associated with the study [28]. *No safety concerns were identified in this study.*
McAllister M.J. *et al.* 2021 [29]	Randomized, double-blind, placebo-controlled, crossover study	14 healthy young subjects, (23 ± 2 yrs.)	0, 6 mg/day	4 weeks	✓ Oxidative Stress, (exercise) ✓ Sports performance	Glutathione was ~7% higher following AX compared with placebo ($p<0.05$). No effect on plasma hydrogen peroxide or MDA ($p>0.05$). Advanced oxidation protein products (AOPP) reduced by ~28% (N.S.; $p = 0.45$). Not affect substrate utilization during exercise. *No safety concerns were identified in this study.*
Nakanishi R. *et al.* 2021 [30]	Randomized, double-blind, placebo-controlled prospective study	29 nursing home resident's healthy elderly subjects (80.9 ± 1.5 yrs.)	0, 12 mg/ twice a day* (0, 24 mg/day)	16 weeks	✓ Oxidative Stress, (Elderly subjects) ✓ Musculoskeletal function	Decrease in d-ROM values with AX group ($p<0.01$), but not placebo group; the AX group had a therapeutic effect on 6-min walking distance compared with the placebo group ($p<0.05$). AX group had an increase in distance and number of steps in the 6-min walking test compared to the placebo group. Furthermore, the rate of increase in blood lactate levels after walking was lower in the AX group than in the placebo group ($p<0.01$). *No safety concerns were identified in this study.*
Shokri-Mashhadi, N. *et al.* 2021 [31]	Randomized, double-blind, placebo-controlled prospective study	44 patients with type 2 diabetes	0, 8 mg/day	8 weeks	✓ Oxidative Stress, (Type 2 diabetes) ✓ Type 2 diabetes	Decrease plasma levels of MDA and IL-6 ($p<0.05$) and decrease the expression level of miR-146a, associated with inflammatory markers (fold change: -1/388) ($p<0.05$). *No safety concerns were identified in this study.*

Urakaze M *et al.* 2021 [32]	Randomized, double-blind, placebo-controlled prospective study	44 subjects Including Prediabetes (Av. 46-48 yrs.)	0, 12 mg/day	12 weeks	✓ Type 2 diabetes<br>✓ Metabolic syndrome<br>✓ Bioavailability, ADME	Glucose levels at 120 minutes after the 75 g oral glucose tolerance test (OGTT) were significantly lower than before supplementation. HbA1c (p<0.05), apo E (p<0.05) and MDA modified LDL (p<0. 05) also decreased, while total cholesterol, triglycerides, and HDL-C levels were unchanged. Matuda index, a measure of insulin resistance, was improved in AX-treated subjects compared to pre-treatment. Plasma AX levels were undetectable at baseline and increased to 122.69 ng/mL (*c.a.* 205 nM) after 4 weeks in the intervention group, and this level was maintained until 12 weeks.<br>*No safety concerns were identified in this study.*
Birudaraju D. *et al.* 2020 [33]	Randomized, double-blind, placebo-controlled prospective study	22 healthy subjects (48.8 ± 16.0 yrs.)	0, 6 mg/ twice a day* (0,12 mg/day)	4 weeks	✓ Cardiovascular health	*Combination with Cavacurcumin, Eicosapentaenoic acid (Omega-3s), AX and γ-linoleic acid (Omega-6) (CEAG)*<br>The CEAG group had significantly lower mean systolic blood pressure at 4 weeks [4.7 ± 6.8 (p = 0.002)] compared to the placebo group. A significant decrease in high-sensitivity C-reactive protein (hsCRP) (-0.49 ± 1.9 vs + 0.51 ± 2.5, p = 0.059) and a blunt increase in IL-6 (+0.2 vs + 0.4, placebo = 0.60) were observed compared to placebo.<br>*No safety concerns were identified in this study.*
Hayashi M. *et al.* 2020 [34]	Randomized, double-blind, placebo-controlled prospective study	54 healthy subjects (Av. 45-46 yrs.)	0, 12 mg/day from *Paracoccus*	8 weeks	✓ Stress/Mood<br>✓ Sleep Quality<br>✓ Bioavailability, ADME	After 8 weeks of AX administration, the serum AX concentration increased to 0.171 ± 0.082 μg/mL (0.29 μM). Not detected in the placebo group.<br>Evaluated with Profile of Mood States 2nd Edition for stress (POMS2) and Oguri–Shirakawa–Azumi Sleep Inventory. Did not observe any significant intergroup differences in the stress and sleep. Subgroup analysis (>65 and ≤65 in the "Depression–Dejection"in POMS): sleep of subjects who scored >65 ("Depression–Dejection") showed significant improvement in the AX group compared with the placebo group, but no significant improvement was observed in stress and the other subjects.<br>*No safety concerns were identified in this study.*
Kato T. *et al.* 2020 [35]	Open-labeled, prospective study	16 patients with systolic heart failure	12 mg/day*	3 months	✓ Cardiovascular health<br>✓ Musculoskeletal function	Increased left ventricular ejection fraction (LVEF) from 34.1 ± 8.6% to 38.0 ± 10.0% (p = 0.031) and 6-min walk distance increased from 393.4 ± 95.9 m to 432.8 ± 93.3 m (p = 0.023). Significant relationships were observed between percent changes in dROM level and those in LVEF.<br>*No safety concerns were identified in this study.*
Kumalic SI. *et al.* 2020 [36]	Randomized, double-blind, placebo-controlled prospective study	72 patients with oligo-astheno-teratozoospermia (Av. 35-36 yrs)	0, 16 mg/day	3 months	✓ Infertility	AX group no improvements in the total number of spermatozoa, concentration of spermatozoa, total motility of spermatozoa, morphology of spermatozoa, DNA fragmentation and mitochondrial membrane potential of spermatozoa or serum follicle-stimulating hormone were determined in patients with oligo-astheno-teratozoospermia.<br>*No safety concerns were identified in this study.*

Sudo A et al. 2020 [37]	Placebo-controlled prospective study	11 healthy subject (College floorball athletes for women in physical education	0.3 mg/day* in V7	30 days	✓ Fatigues<br>✓ Sports performance<br>✓ Eye health<br>✓ Skin health	*Test supplement (V7; astaxanthin, reduced coenzyme Q10, leucine, arginine, citrulline, DHA, Krill oil).* Studied the efficacy of V7 on subjective fatigue, sports performance and skin conditions in floorball athletes. Subjective symptoms were evaluated with the VAS. The change of VAS in PRE and POST in the V7 group showed that fatigue was significantly alleviated overall and in the torso. Significantly improved on a seated toe touch, increasing from 48.6 cm pre-intake to 51.8 cm post-intake. *No safety concerns were identified in this study.* [Article in Japanese]
Chan K. *et al.* 2019 [38]	Randomized, double-blind, placebo-controlled prospective study	54 patients With type 2 diabetes	0, 6, 12 mg/day	8 weeks	✓ Type 2 diabetes<br>✓ Cardiovascular health<br>✓ Metabolic syndrome	Increased plasma AX levels and decreased fasting plasma glucose and HbA1c levels. In 12mg AX group, reduced in plasma triglyceride, total cholesterol and LDL levels. Lowered changes in plasma IL-6 and TNF-α levels and plasma vWF level and higher changes in AT-III level. In 12mg AX group, decreased changes in plasma FVII and PAI-1levels. *No safety concerns were identified in this study.*
Fleischmann C. *et al.* 2019 [39]	Randomized, double-blind, placebo-controlled prospective study	22 healthy subjects (23.1 ± 3.5 yrs.)	0, 12 mg/day	30 days	✓ Sports performance<br>✓ Energy metabolisms<br>✓ Heat strain/stress	Decreased raised in blood lactate caused by the $VO_{2\ Max}$ test; AX group (9.4±3.1 and 13.0±3.1 mmole·l^{-1} in the AX and Placebo groups, respectively $p<0.02$). Change in oxygen uptake during recovery (-2.02±0.64 and 0.83±0.79% of $VO_{2\ Max}$ in the AX and Placebo group, respectively, $p=0.001$). *N.S*; anaerobic threshold or VO_2 Max. physiological or biochemical differences in the heat tolerance test (HTT) (2 h walk at 40°C, 40% relative humidity. *No safety concerns were identified in this study.*
Hashimoto H. *et al.* 2019 [40]	Open-labeled, prospective study	35 subjects who underwent bilateral cataract surgery (intraocular lens implantation) (Mean *c.a* 71 yrs.)	6 mg/day	2 weeks	✓ Eye health<br>✓ Oxidative stress (Cataract surgery)	In this analysis, the effect of AX intake on the relationship between VEGF levels and ROS-related parameters before and after bilateral cataract surgery was analyzed by gender. VEGF, hydrogen peroxide, and total hydroperoxide levels in the aqueous humor, as well as O2 scavenging activity, were measured. For women only, VEGF levels and O2 scavenging activity before AX intake were negatively correlated (r = -0.6, p<0.01) and positively correlated with total hydrogen peroxide levels before and after AX intake (r = 0.7, 0.8, p<0.01, respectively).<br>Analysis associated with the study [23]<br>*No safety concerns were identified in this study.*
Landi F *et al.* 2019 [41]	Randomized open-labeled, prospective study	47 subjects with higher level of serum Chol. (not needing statins or statin intolerant) (58.7± 8.7 yrs.)	0.5 mg/day* in Nutraceutical B	6 weeks	✓ Cardiovascular health<br>✓ Metabolic syndrome	*Nutraceutical B: policosanol (10 mg), red yeast rice (200 mg; 3 mg monacolin K), Berberine (500 mg), Astaxanthin (0.5 mg), folic acid (200 mcg), and Coenzyme Q10 (2 mg)*<br>Both nutraceutical combinations improved the lipid profile including total Chol., HDL-Chol., LDL-Chol., and TG.<br>*No safety concerns were identified in this study.*

Sudo A *et al.* 2019 [42]	Open-labeled, prospective study	19 healthy females (Mean 47.3 yrs.)	0.3 mg/day* in V7	30 days	✓ Fatigues<br>✓ Skin health<br>✓ (Eye health)<br>✓ (Cognitive function)	*Test supplement (V7; astaxanthin, reduced coenzyme Q10, leucine, arginine, citrulline, DHA, Krill oil).* Studied the efficacy of V7 on subjective fatigue and skin conditions in typical middle aged females. Subjective symptoms were evaluated with the VAS. The change of VAS in PRE and POST in the V7 group showed statistically significantly improved general fatigue, leg fatigue and the state of the lower back (p<0.05, respectively); significantly improved dark spots, blotches, and the elasticity and appearance of the skin; no significant change in eye strain and memory loss.<br>*No safety concerns were identified in this study.* [Article in Japanese]
Sudo A *et al.* 2019 [43]	Placebo-controlled prospective study	19 healthy subject (College softball player for women in physical education	0.3 mg/day* in V7	30 days	✓ Fatigues<br>✓ Sports performance<br>✓ Eye health<br>✓ Skin health	*Test supplement (V7; astaxanthin, reduced coenzyme Q10, leucine, arginine, citrulline, DHA, Krill oil).* Studied the efficacy of V7 on subjective fatigue, sports performance and skin conditions in college softball players. Subjective symptoms were evaluated with the VAS. The change of VAS in PRE and POST in the V7 group showed statistically significant improved fatigue, shoulder pain, skin blemishes, and 50 m running performance; a comparison of V7 and placebo POST showed statistically significant increases in leg fatigue, knee and hip pain, skin elasticity and whitening, memory loss, and eye strain. The percent change between PRE and POST in V7 was statistically significantly higher in leg fatigue, hip and back pain, dull skin, eye strain, and total score.<br>*No safety concerns were identified in this study.* [Article in Japanese]
Takami M. *et al.* 2019 [44]	Open-labeled, prospective study	20 healthy young male subjects	*c.a*, 4.5 mg/day* from salmon	4 weeks	✓ Sports performance<br>✓ Energy metabolisms	Increased maximum work load by training in both groups (p = 0.009), while increased oxygen consumption during exercise in the antioxidant group only (p=0.014). There were positive correlations between maximum work load and fat (r=0.575, p=0.042) and carbohydrate oxidations (r =0.520, p=0.059) in the antioxidant group. Higher carbohydrate oxidation during rest in the post-training than that in the pre-training only in the antioxidant group. More decreased levels of serum insulin and HOMA-IR after training were observed in the antioxidant group than in the control group.<br>*No safety concerns were identified in this study.*
Terai K. *et al.* 2019 [45]	Randomized, two-arm, open-labeled, prospective study	31 males with oligozoospermia and/or asthenozoospermia	0 (HE), 16mg/day*	12 weeks	✓ Infertility	*Intervention; Combination of antioxidant supplements (L-carnitine, Zn, CoQ10, vitamin C, vitamin B12, vitamin E and AX) and a Chinese herbal medicine, hochu-ekki-to (HE).* No significant improvements in endocrinological findings in the supplement group. Although no statistically significant improvement was observed in semen volume, sperm concentration, and sperm motility semen findings, whereas total motile sperm counts were significantly improved. On the other hand, HE group tended to increase semen concentration, semen motility, and total motile sperm count, but not significantly improve any endocrinological factors or semen findings.<br>*No safety concerns were identified in this study.*

Hayashi M. *et al.* 2018 [46]	Randomized, double-blind, placebo-controlled prospective study	54 healthy subjects (45–64 yrs.)	0, 8 mg/day from *Paracoccus*	8 weeks	✓ Cognitive function<br>✓ Bioavailability, ADME	After 8 weeks of AX administration, the serum AX concentration increased to 0.173 ± 0.058 μg/mL (0.29 μM). Not detected in the placebo group.<br>Evaluation: word memory test, verbal fluency test, and Stroop test.<br>AX group significantly larger increase in blood AX level.<br>No significant intergroup differences in the results of the evaluations.<br>Subgroup analysis(<55 yrs. old and ≥55 yrs. Old): "words recalled after 5 minutes" in word memory test in <55 years old subjects showed significant improvement in the AX group than in the placebo group, which was not found in ≥55 years old subjects.<br>*No safety concerns were identified in this study.*
Hongo N. *et al.* 2018 [47]	Randomized, double-blind, placebo-controlled prospective study	40 Healthy subjects aged 60-79 years reporting awareness of cognitive and/or physical decline (Mean 65.8 yrs.)	0, 12 mg/day*	12 weeks	✓ Cognitive function<br>✓ Fatigues<br>✓ Mood/Stress	*AX group (12 mg AX, 10 mg tocotrienols, 6 mg zinc and 600 IU vitamin D) and control group (same formulation w/o. AX).* Cognitive functions/Fatigues: AX intake shortened the reaction time in working-memory tasks (particularly improved the speed of processing newly-provided visual information by comparing it with previously-provided information while appropriately retaining the previous information in the memory for several seconds). In addition, AX promoted efficacy in the improvement of self-assessed memory ability. Furthermore, in the subjects with improved endurance, AX enhanced the recognition memory function on nonverbal information, particularly it improved the accuracy of memorizing faces and distinguish them from newly-presented faces. Moods/Stress: Improved the scores of "Anger-Hostility," "Confusion-Bewilderment," "Fatigue-Inertia," and TMD in the POMS survey and the decrease in the score of "irritated" in the VAS survey in the AX group<br>*No safety concerns were identified in this study.* [Article in Japanese]
Imai A. *et al.* 2018 [48]	Randomized, double-blind, placebo-controlled crossover study	42 healthy subjects	0, 6 mg/day*	4 weeks	✓ Oxidative stress (during mental and physical tasks)<br>✓ Fatigues<br>✓ Mood/Stress	Elevated PCOOH levels during mental and physical tasks were attenuated by AX supplementation. Improved recovery from mental fatigue compared to placebo. No differences were found between AX and placebo in other secondary outcomes such as subjective feelings, work efficiency, and autonomic activity. The treatment group showed a significant reduction ($p<0.05$) in fatigue during recovery after the mental task compared to the placebo group. (Evaluated by VAS)<br>*No safety concerns were identified in this study.*
Ito N. *et al.* 2018 [49]	Randomized, double-blind, placebo-controlled, prospective study	22 healthy subjects with skin phototype was type II or III (30-56 yrs.)	0, 6 mg/day	10 weeks	✓ Skin health<br>✓ Oxidative stress (UV irradiation)	Subjective skin condition was assessed on a visual analog scale; the AX group showed an increase in minimum erythema dose (MED) compared to placebo. AX group had a reduced loss of skin moisture in the irradiated area compared with placebo. Subjective skin conditions for "improvement of rough skin" and "texture" in non-irradiated areas were significantly improved by AX.<br>*No safety concerns were identified in this study.*

Ito N. *et al.* 2018 [50]	Randomized, double-blind, placebo-controlled, prospective study	14 patients diagnosed with MCI (57-78 yrs.; MMSE scores 24-27).	0, 6 mg/day*	12 weeks	✓ Cognitive function	Evaluation of cognitive improvement in patients with mild cognitive impairment (MCI). The Central Nervous System Vital Signs (CNSVS, also known as 'Cognitrax') test: significantly improved "psychomotor speed" and "processing speed" in the AX group compared to the placebo group.<br>*No safety concerns were identified in this study.*
Liu S.Z. *et al.* 2018 [28]	Randomized, double-blind, placebo-controlled, prospective study	42 elderly subjects (65-82 yrs.)	0, 12 mg/day*	12 weeks	✓ Musculoskeletal function<br>✓ Energy metabolisms	Administration of AX increased maximal voluntary force (MVC) by 14.4% (± 6.2%, $p<0.02$), tibialis anterior muscle size (cross-sectional area, CSA) by 2.7% (± 1.0%, $p<0.~.01$), and specific impulse increased by 11.6% (MVC/CSA, ± 6.0%, $p = 0.05$), respectively, whereas placebo treatment did not alter these characteristics (MVC, 2.9% ± 5.6%; CSA, 0.6% ± 1.2%; MVC/CSA, 2.4 ± 5.7%; all $p>0.6$).<br>*No safety concerns were identified in this study.*
Madhavi D. *et al.* 2018 [51]	Open-label, crossover study	6 healthy subjects	60 mg	Single dosing	✓ Bioavailability, ADME	*Test articles AX-SR; 2.5% AX in a sustained-release matrix, AX (control); 10% AX (unformulated AX oil)* Dissolution study: AX-SR formulation formed a stable dispersion in simulated gastric and intestinal fluids. Single-dose pharmacokinetic study (clinical study); AX-SR formulation (AUC_{0-24h}: 4393 ± 869 ng/mL-h) showed 3.6 times higher bioavailability than AX oil (AUC_{0-24h}: 1227 ± 1328 ng/mL-h) ($p<0.0005$).<br>AX oil showed very low absorption in 3 of 6 subjects, whereas AX-SR formulation appeared in the blood of all subjects.<br>*No safety concerns were identified in this study.*
Mashhadi N.S. *et al.* 2018 [52]	Randomized, double-blind, placebo-controlled, prospective study	44 participants with type 2 diabetes	0, 8 mg/day	8 weeks	✓ Type 2 diabetes<br>✓ Cardiovascular health<br>✓ Metabolic syndrome	Increased the serum adiponectin concentration, reduced visceral body fat mass ($p<0.01$), serum triglyceride and very-low-density lipoprotein (VLDL) cholesterol concentrations, systolic blood pressure, fructosamine concentration ($p<0.05$) and marginally reduced the plasma glucose concentration (p=0.057).<br>*No safety concerns were identified in this study.*
Petyaev I.M., *et al.* 2018 [53]	Randomized, blinded, four-arm, prospective study	32 subjects with oxidative stress, 8 subjects taking AX only, (60-70 yrs)	0, 7 mg/day* with DC	4 weeks	✓ Oxidative Stress (elderly subjects)<br>✓ Bioavailability, ADME	Reduced serum oxidized LDL by 55.4% after 4 weeks ($p<0.05$). Reduced MDA by 52.7% after 4 weeks ($p<0.05$). Increase in serum nitric oxide (NO) levels ($p = 0.054$), plasma oxygen transport, tissue oxygen saturation and plasma AX concertation seen in the volunteers supplemented with lycosomal formulation of dark chocolate (DC) containing 7 mg of co-crystalized AX (L-DC-ASTX) formulation. Increase in plasma AX concertation. Plasma AX concentrations after 4 weeks were higher in the L-DC-ASTX formulation than other groups (5.22-7.31nmol/L vs. 17.34 nmol/L)<br>*No safety concerns were identified in this study.*

Sarkkinen ES., *et al.* 2018 [54]	Randomized, double-blind, placebo-controlled, prospective study	35 overweight subjects with mildly or moderately elevated blood pressure	N/A (0, 4g of kirill oil powder /day)	56 days	✓ Safety<br>✓ Cardiovascular health<br>✓ Metabolic syndrome	Average values of haematological measurements were within the reference range for all subjects, and no significant changes were observed in blood pressure or lipid levels.<br><br>*No serious adverse events were reported.*
Canas J. A. *et al.* 2017 [55]	Randomized, double-blind, placebo-controlled, prospective study	20 children with simple obesity (BMI > 90%)	500 µg/day* (MCS)	6 months	✓ Obesity<br>✓ Metabolic syndrome	Mixed-carotenoid supplementation (MCS) increased β–carotene, total adiponectin, and high-molecular-weight adiponectin in plasma compared to placebo; MCS decreased BMI z-score, waist-to-height ratio, and subcutaneous adipose tissue compared to placebo. AX was used as a part of MCS.<br>*No safety concerns were identified in this study.*
Carrascosa JM *et al.* 2017 [56]	Randomized, double-blind, placebo-controlled, prospective study	31 healthy subjects with skin phototypes II and III	4 mg/day*	56 days	✓ Skin health	*Intervention: Genosun oral® a combinationof AX (4 mg), β-carotene(4.8 mg), vitamin E (6 mg), vitamin C(40 mg), lutein (2.4 mg) and lycopene (2.4 mg).* MED at Day1, 29 and 57 was evaluated. Intervention group was significant increase over placebo in the tolerance to an erythemal dose of UVR. Increased UVR tolerance was reflected in an increase in MED of 12.4% and 20.51% over baseline after 29 and 57 days, respectively, with and a significant difference between treatment and control groups at the end of the study.<br>*No safety concerns were identified in this study.*
Chalyk, N. *et al.* 2017 [57]	Open-label, prospective study	31 subjects; 18 obese, 8 overweight, 5 healthy, over the age of 40	4 mg/day	92 days	✓ Oxidative Stress (middle age, obesity)<br>✓ Skin health	Plasma MDA decreased with AX by 11.2% on day 15 and by 21.7% on day 29 (*N.S.*) Morphological analysis of the residual skin surface components (RSSC; age-related changes in corneocyte desquamation, microbial presence, and lipid droplet size): decreased levels of corneocyte desquamation (p=0.0075) and microbial presence (p=0.0367), increase in lipid droplet size among obese (body mass index >30 kg/m2) subjects (p=0.0214).<br>*No safety concerns were identified in this study.*
Chen JT, Kotani K. 2017 [58]	Randomized, double-blind, placebo-controlled, prospective study	29 healthy females	0, 12 mg/day	3 months	✓ Liver protection<br>✓ Immunity	Significant decrease in the levels of serum liver enzymes, such as aspartate aminotransferase (AST) and alanine aminotransaminase (ALT), in AX treated groups. No significant changes in the levels of serum d-ROMs, urinary 8-OHdG and BAP following AX treatment. Significant increase in blood leukocytes was also found in AX-treated group.<br>*No safety concerns were identified in this study.*
Hongo N. *et al.* 2017 [59]	Randomized, double-blind, placebo-controlled, prospective study	39 healthy subjects	0, 12 mg/day*	12 weeks	✓ Oxidative stress (during mental and physical tasks)<br>✓ Fatigues<br>✓ Mood/Stress	Intent-to-treat (ITT) analysis; fatigue after physical and mental stress was significantly lower in the AX group than in the placebo at week 8; the change in POMS Friendliness was significantly higher in the AX group than in the control group at week 8; the rate of change in BAP values at week 12 was not significantly different between the AX and control groups. The rate of change in BAP values at week 12 was not significantly different between the AX group and the control group.<br>*No safety concerns were identified in this study.* [Article in Japanese]

Ledda A. *et al.* 2017 [60]	Open-labeled, two-arm prospective study	59 patients with genitourinary cancers (prostate or bladder malignancies) who had undergone and completed cancer treatments (radiotherapy, chemotherapy or intravesical immunotherapy with increased oxidative stress and residual symptoms)	0, 8 mg/day*	6 weeks	✓<br>✓	Improving QOL in Cancer Therapy<br>Oxidative stress (Cancer Therapy)	*Oncotris: containing 264mg/day curcumin, 500mg/day extract of cordyceps, and 8mg/day AX (from EP217785227).* Signs and symptoms (treatment-related) and the intensity of residual side effects were significantly reduced after 6 weeks in the supplemented group: minimal changes were seen in the control group.<br>Oncotris supplementation was associated with significant improvements in blood cell counts and reductions in levels of plasma PSA and oxidative stress.<br>*No safety concerns were identified in this study.*
Kaneko M *et al.* 2017 [61]	Open-label, prospective study	10 male subjects	24 mg/day	28 days	✓	Vocal fold health	Aerodynamic assessment, acoustic analysis, and GRBAS scale (grade, roughness, breathiness, asthenia, and strain) were significantly worse in the AX(-) status (day 0) immediately after vocal loading, but improved by 30 minutes after loading. On the other hand, in AST(+) (day 35), no statistical worsening of any of the phonatory parameters was observed when measured immediately after the vocal load.<br>*No safety concerns were identified in this study.*
Marazzi G *et al.* 2017 [62]	Randomized, single-blind, placebo-controlled, prospective study	100 patients with coronary artery disease, percutaneous coronary intervention in the past 12 months, high-dose statin intolerance, and LDS treatment alone did not reduce LDL-C by more than 50%.	0, 0.5 mg/day* (Armolipid Plus)	12 months	✓<br>✓	Cardiovascular health<br>Metabolic syndrome	Aim was to compare the efficacy and tolerability of low-dose statin (LDS) therapy versus combined therapy of LDS plus a nutraceutical combination (Armolipid Plus : red yeast rice (200 mg), policosanol (10 mg), berberine (500 mg), folic acid (0.2 mg), AX (0.5 mg), and coenzyme Q10 (2 mg))<br>After 3 months, LDL-C and total cholesterol were significantly lowered in the LDS + Almolypid Plus (n=50) group (p<0.0001), and 70% of this group achieved the treatment goal (LDL-C <70 mg/dl), while patients in the LDS group did not.<br>*No safety concerns were identified in this study.*
Saito H *et al.* 2017 [63]	Randomized, double-blind, placebo-controlled prospective study	120 healthy subjects	0, 1.5, 3 mg/day* (1.5 mg from krill, 3.0 mg from *Haematococcus* algae)	12 weeks	✓	Sleep Quality	Subjects were divided into four groups: (A) placebo, (B) zinc-rich food, (C) zinc-, and AX-rich food (krill), and (D) placebo supplemented with zinc-enriched yeast and AX oil (from *Haematococcus* algae).<br>Pittsburgh sleep quality index (PSQI): improved significantly within all groups at endpoint.<br>Total sleep time (TST): No significant difference was noted in any groups in comparison to the group A.<br>Sleep onset latency (SOL): significantly improved in the group B and the group D in comparison to the placebo group.<br>Body positional changes: Although the number increased in the group A (3.74 ± 4.38), this increase was suppressed in the group B(0.71 ± 5.01), group C (0.58 ± 5.75) and the group D (0.95 ± 4.33) (*N.S.*).

						No safety concerns were identified in this study.
Tominaga K. *et al.* 2017 [64]	Randomized, double-blind, placebo-controlled prospective study	65 healthy female (age, 35–60 years) with a wrinkle grade of 2.5 to 5.0	0, 6, 12 mg/day	16 weeks	✓ Skin health	Water content (cheeks): no significant difference from the placebo group regarding improvement. However, significantly worse in the placebo group over the period, but unchanged in the low and high AX-treated groups. TEWL (cheek): no significant change during the study.<br>Wrinkle depth (eye rims): No significant difference. However, it worsened during the study period in the placebo group, but did not change in the low and high AX-treated groups.<br>Elasticity (cheeks): significantly improved in the two AX groups<br>IL-1α: increased during the study, but not in the high AX dose group (p<0.05)<br>*No safety concerns were identified in this study.*
Coombes J.S *et al.* 2016 [65]<br>Fassett, R.G. *et al.* 2008, [66]	Randomized, double-blind, placebo-controlled prospective study	58 renal transplant recipients	0, 12 mg/day	12 months	✓ Oxidative stress<br>✓ Vascular health<br>✓ Bioavailability, ADME<br>(in renal transplantation)	Plasma AX concentrations were 0.29 ± 0.18 μmol/L at 6 months and 0.28 ± 0.17 μmol/L at 12 months (Mean ± SD). No effect on arterial stiffness, oxidative stress, or inflammation in renal transplant recipients.<br>(The XANTHIN trial)<br>*No safety concerns were identified in this study.*
Hashimoto H. *et al.* 2016 [67]	Open-labeled, prospective study	35 subjects who underwent bilateral cataract surgery (intraocular lens implantation) (Mean *c.a* 71 yrs.)	6 mg/day	2 weeks	✓ Oxidative Stress (during surgery)<br>✓ Eye health	Superoxide anion scavenging activity (U/ml) 18.2 ± 4.1 at 0 weeks reduced to 19.9 ± 3.6 after 2 weeks of supplementation compared to baseline, p<0.05 Total hydroperoxides (U CARR) from 1.16 ± 0.18 at 0 weeks reduced to 1.04 ± 0.31 after 2 weeks of supplementation compared to baseline, p<0.05<br>Analysis associated with the study [23].<br>*No safety concerns were identified in this study.*
Huang J.Y. *et al.* 2016 [68]	Randomized, double-blind, placebo-controlled prospective study	43 patients with dry eye disease (DED)	0, 2 mg/day*	16 weeks	✓ Eye health (DED)	*Supplement: Commercially available antioxidant supplement containg anthocyanosides, vitamins A, C, and E, and crude extracted additives from several Chinese herbal extracts and AX.* Lower diastolic blood pressure in the treated group. No statistically significant differences in systolic blood pressure, dry eye symptoms, serum anti-SSA and anti-SSB, visual acuity, intraocular pressure, or fluorescein corneal staining between the two groups. Significantly improved tear film break time scores and Schirmer test without local anesthesia in the treatment group. Tear ROS levels differed between groups and decreased after treatment. Overall subjective impression was significantly improved by treatment compared to placebo.<br>*No safety concerns were identified in this study.*
Talbott I. *et al.* 2016 [69]	Randomized, double-blind, placebo-controlled prospective study	28 recreational runners (42±8 yerars)	0, 12 mg/day	8 weeks	✓ Sports performance	Reduced average heart rate at submaximal endurance intensities (aerobic threshold, AeT and anaerobic threshold, AT), but not at higher "peak" intensities.<br>*No safety concerns were identified in this study.*

Tsukahara H. *et al*, 2016 [70]	Randomized, double-blind, placebo-controlled prospective study	40 healthy subjects, those concerned about skin dullness or age-related skin deterioration.	0, 3 mg/day	8 weeks	✓ Skin health	TEWL (left cheek): significantly improved. Moisture content (left cheek): Significant improvement. Melanin and color difference (left cheek): Melanin improved significantly. Elasticity (left cheek): Significant improvement in partial (R6). Facial image analysis (left part): "texture" significantly improved. *No safety concerns were identified in this study.* [Article in Japanese]
Andrisani A. *et al*, 2015 [71]	N/A	24 Semen samples from healthy male donors of proven fertility, 27 Semen samples from patients who had failed to conceive after at least one year of regular unprotected intercourse	0, 2 μM	N/A	✓ Infertility<br>✓ Assisted reproductive technology (ART)	Evaluation of sperm capacitation, which involves a series of modifications, including regulation of reactive oxygen species, depletion of cholesterol in the sperm outer membrane, and protein tyrosine phosphorylation (Tyr-P) processes in the head region, that acquire sperm to the essential functions for fertilization of oocytes. AX successfully triggered Lyn translocation in patient-derived sperm (PG), bypassing the impaired ROS-related mechanisms for raft and Lyn translocation. In this study, ROS generation and lipid raft and Lyn relocation are interdependent, leading to a continuous cellular acrosome reaction (AR). AX could be potentially used to ameliorate male idiopathic infertility by improving PG sperm function.<br>A study related to the ref. [72].<br>(*in vitro* study)
Baralic, I. *et al.* 2015 [73]	Randomized, double-blind, placebo-controlled, prospective study	40 healthy subjects (young soccer players)	0, 4 mg/day	90 days	✓ Oxidative Stress (during exercise)<br>✓ Immunity	The increase in neutrophil count and hs-CRP level was found only in placebo group, indicating a significant blunting of the systemic inflammatory response in the subjects taking AX.<br>Improved prooxidant-antioxidant balance (PAB; p<0.05)<br>AX supplementation improves sIgA response and attenuates muscle damage<br>*No safety concerns were identified in this study.*
Maki KC. *et al.* 2015 [74]	Randomized, double-blind, placebo-controlled, prospective study	102 subjects with TAG 150-499 mg/dL and LDL cholesterol (LDL-C) ≥70 mg/dL	0, 12 mg/day*	8 weeks	✓ Cardiovascular health<br>✓ Metabolic syndrome	*Test food (PDL-0101): 1.8 g/day eicosapentaenoic acid, 100 mg/day tocopherol-free γ/δ tocotrienols enriched with geranylgeraniol, extracted from annatto and 12 mg/day AX.*<br>After 8 weeks of treatment, PDL-0101 significantly reduced median TAG compared to placebo (-9.5% vs. 10.6%, p<0.001), but there was no significant change in mean LDL-C (-3.0% vs. -8.0% for PDL-0101 and placebo, respectively, p=0.071), no significant change in mean high-density lipoprotein cholesterol (approximately 3% reduction in both groups, p=0.732), or median oxidized LDL concentration (5% vs. -5% for PDL-0101 and placebo, respectively, p=0.112).<br>*No safety concerns were identified in this study.*

Nakayama T. *et al.* 2015 [75]	Open-labeled, prospective study	18 subjects with normal menstrual cycles who have severe menstrual cramps or hypermenorrhea	12 mg/day	12 weeks	✓<br>✓	Women's health<br>Dysmenorrhea	<u>VAS values for menstrual cramps</u>: After 12 weeks of AX supplementation, decreased significantly with duration of supplementation (*p*<0.05 vs. pre, pre; 60.2±16.3, 4 weeks; 45.3±17.0, 12 weeks; 38.5±19.0). After12 weeks washout, increased to 55.0±18.3 (*p*<0.05 vs. 12 weeks). <u>VAS values for menstrual flow</u>: Significantly decreased at 12 weeks (*p*<0.05, pre; 67.0±15.7 vs. 12 weeks; 45.1±20.3), but increased again after 12 weeks washout (55.6±16.6, *p*<0.05 vs 12 weeks). <u>Day-dysmenorrhea</u>: Significantly decreased at 12 weeks (*p*<0.05, pre; 2.5±1.8 vs. 12 weeks; 1.3±0.8), but increased again after 12 weeks washout (2.5±2.0, *p*<0.05 vs 12 weeks). <u>Others</u>: No significant changes in the frequency of NSAIDs dosing and the blood levels of CA125 and Hb over the study period.<br>*<u>No safety concerns were identified in this study.</u>* [Article in Japanese]
Phetcharat L. *et al.* 2015 [76]	Randomized, double-blind, placebo-controlled, prospective study	34 healthy subjects with wrinkles on the face (crow's-feet) (35–65 yrs.)	4 mg/day	8 weeks	✓	Skin health	*The comparative control was rose hip powder. Therefore, the results of the evaluation before and after the intervention in the AX group are shown*. <u>Moisture content</u> (forehead): improved from the pre-treatment level at week 8 (*p*<0.001) in the AX group. <u>Elasticity</u> (cheeks): improved from the pre-treatment level in the AX group at week 8 (*p*<0.05). <u>Crow's-feet wrinkle depth</u> (buttocks of the eyes): improved from pre-treatment levels at Weeks 4 and 8 in the AX group (*p*<0.05).<br>*<u>No safety concerns were identified in this study.</u>*
Takemoto M. *et al.* 2015 [77]	Case report	1 Werner syndrome patient	12 mg/day*	6 months	✓	Werner syndrome (Metabolic syndrome)	Improved blood transaminase concentrations; before AX intervention and 3 and 6 months after initiation were AST 40 IU/L, 41 IU/L, and 20 IU/L; ALT 69 IU/L, 62 IU/L, and 34 IU/L; GGT 38 IU/L, 41 IU/L, and 35 IU/L; and cholinesterase 360 IU/L, 366 IU/L, and 331 IU/L, respectively. Liver-to-spleen Hounsfield units on CT were 0.41 before AX initiation, 0.71 at 3 months, and 0.94 at 6 months. No significant changes after AX intervention in hyaluronic acid, a marker of liver fibrosis; high-sensitivity C-reactive protein, a marker of inflammation; and MDA-modified LDL.<br>*<u>No safety concerns were identified in this study.</u>*
Ni Y. *et al.* 2015 [78]	Randomized, single-blind, placebo-controlled, prospective study	12 NASH patients	0, 12 mg/day	24 weeks	✓	Metabolic syndrome (NASH)	Improved steatosis (*p*<0.05), marginally improved lobular inflammation (*p*=0.15) and NAFLD activity score (*p*=0.08)<br>*<u>No safety concerns were identified in this study.</u>*

Balcerczyk A. *et al.* 2014 [79]	Randomized, double-blind, placebo-controlled prospective study	66 healthy females, (35–55 yrs.)	0, 15 mg/day*	12 weeks	✓ Oxidative Stress<br>✓ Antiaging	*Test supplement (NucleVital Q10); omega-3 acids (1350 mg/day), ubiquinone (300 mg/day), lycopene (45 mg/day), lutein palmitate (30 mg/day), zeaxanthin palmitate (6 mg/day), L-selenomethionine (330 mg/day), cholecalciferol (30 μg/day), α-tocopherol (45 mg/day) and AX (15 mg/day).* Oxidative stress: significantly increased TAC of plasma and activity of erythrocyte SOD, with slight effects on oxidative stress biomarkers in erythrocytes; MDA and 4-hydroxyalkene levels. Antiaging effect: significant changes in mRNA expression of SIRT1 and 2 in PBMCs. The level of telomerase was also increased by more than 25%, although the length of lymphocyte telomeres.<br>*No safety concerns were identified in this study.*
Kono K. *et al.* 2014 [80]	Randomized, double-blind, placebo-controlled prospective study	48 healthy subjects who complained of eye strain	0, 4 mg/day*	4 weeks	✓ Eye health<br>✓ Fatigue (Shoulder Stiffness)	*Test supplement (Enkin); lutein (10mg), 20 mg of bilberry extract and 26.5 mg of black soybean hull extract (a total of 2.3 mg of cyanidin-3-glucoside in both extracts), DHA (50mg) and AX (4mg)*<br>The variation of the "near-point accommodation" of both eyes from baseline to 4 weeks after-intervention in the test supplement group (TS) was significantly higher than in the placebo (P) group (1.321±0.394 diopter (D) in the TS group and 0.108±0.336 D in the P group, *p*=0.023, respectively). Regarding subjective symptoms, significant improved on "stiff shoulders or neck" and "blurred vision" in TS group compared to the P group ($p<0.05$).<br>*No safety concerns were identified in this study.*
Yoon HS. *et al.* 2014 [81]	Randomized, double-blind, placebo-controlled prospective study	44 healthy females with wrinkles grade ≥2 (≥40 yrs.)	0, 2 mg/day*	12 weeks	✓ Skin health<br>✓ Oxidative Stress (UV irradiation)	*AX (2 mg/day) combined with collagen hydrolysate (3 g/day).* Skin condition of non-UV irradiated skin: significantly improved TEWL (cheek) at week 12 (*p*=0.045), tended to improve water content (cheek), tended to improve elasticity (cheek) at week 4, and significantly improved at week 12. Skin biopsy after UV irradiation: no difference in oxidative markers (Thymine dimers, 8-OHdG) between the two groups in histological evaluation. Regarding mRNA expression, significantly upregulated expression of procollagen type I, tended to upregulate fibrillin-1, while significantly downregulated MMP1, MMP12 in the AX group compared to placebo.<br>*No safety concerns were identified in this study.*
Zanotta D. *et al.* 2014 [82]	Open-labeled, prospective study	104 subjects diagnosed with mild cognitive impairment (Mean 71.2 yrs.)	*c.a.* 4.2 mg/day*	60 days	✓ Brain health (Cognitive functions)	*Test supplement (Illumina); 20mg Bacosides from* Bacopa monnieri, *30 mg Phosphatidylserine, 30 mg V.E and 2 mg AX.* Significantly improved in the Alzheimer's Disease Assessment Scale-cognitive subscale (ADAS-cog) total score from 13.7±5.8 at baseline to 9.7±4.9 at 60 days and in the clock drawing test from 8.5±2.3 to 9.1±1.9. The greatest improvement in each component of the ADAS-cog was in the memory task. The largest improvement in each component of the ADAS-cog were in the memory tasks. In multivariate analysis, larger improvements in ADAS-cog scores were associated with less deterioration in baseline Mini-Mental State Examination scores. Efficacy was rated "excellent" or "good" by 62% of subjects. The study compounds were well tolerated, with one non-serious adverse event (gastric disturbances in a subject who was taking concomitant oral

						corticosteroids) reported in the entire study population and tolerability rated as "excellent" or "good" by 99% of subjects
Baralic I. *et al.* 2013 [83]	Randomized, double-blind, placebo-controlled prospective study	40 healthy subjects (soccer players)	0, 4 mg/day	90 days	✓ Oxidative Stress (exercise)	Protected thiol groups against oxidative modification (increase in -SH groups, $p<0.05$; improved PON1 activity towards paraoxon and diazoxon, $p<0.05$ and $p<0.01$, respectively)<br>*<u>No safety concerns were identified in this study.</u>*
Dona G. *et al.* 2013 [72]	N/A	24 Semen samples from healthy male donors	0 to 2 μM	N/A	✓ Infertility<br>✓ Assisted reproductive technology (ART)	The AX improved Tyr-P and acrosome reaction cells (ARC) values in sperm heads without affecting the ROS generation curve, while diamide successfully increased Tyr-P levels in flagella but did not increase ARC values. This suggests that AX, when inserted into the membrane, causes membrane changes, such as capsulation, that allow for Tyr-P conversion of the head. In other words, an acrosome reaction can occur and more cells can be involved.<br>A study related to the ref.[71].<br>(*in vitro* study)
Djordjevic B. *et al.* 2013 [84]	Randomized, double-blind, placebo-controlled prospective study	32 healthy subjects (soccer players)	0, 4 mg/day	90 days	✓ Oxidative Stress (exercise)	Regular training significantly increased $O2^{\bullet -}$ levels (main training effect, $p<0.01$). $O2^{\bullet -}$ concentrations. Not change TBARS and AOPP levels throughout the study. Decreased TAS levels post- exercise only in Placebo group ($p<0.01$). Increased total SH groups content both in AX and in Placebo groups (by 21% and 9%, respectively) and supplementation effect was marginally significant ($p=0.08$). Decreased basal SOD activity both in Placebo and in AX group by the end of the study (main training effect, $p<0.01$). Significant decrease in basal CK and AST activities after 90 days (main training effect, $p<0.01$ and $p<0.001$, respectively). Postexercise CK and AST levels were significantly lower in AX group compared to Placebo group ($p<0.05$)<br>*<u>No safety concerns were identified in this study.</u>*

Hashimoto, H. *et al*. 2013 [85]	Open-labeled, prospective study	35 subjects who underwent bilateral cataract surgery (intraocular lens implantation) (Mean *c.a* 71 yrs.)	6 mg/day	2 weeks	✓ Oxidative Stress<br>✓ Eye health<br>(in aqueous humor of cataract patients)	Reduced total hydroperoxides (hydrogen peroxides, lipid peroxides, and peroxides of protein in aqueous humor; $p<0.05$) increased superoxide scavenging activity ($p< 0.05$)<br>Analysis associated with the study [23]<br>*No safety concerns were identified in this study.*
Klinkenberg L.J. *et al.* 2013 [86]	Randomized, double-blind, placebo-controlled, prospective study	32 well-trained male cyclists (25 ± 5 years, $V^{\cdot}O_{2peak}$ = 60 ± 5 mL·kg⁻¹·min⁻¹, W_{max} =5.4 ± 0.5 W·kg⁻¹)	0, 20 mg/day*	4 weeks	✓ Oxidative Stress (exercise)<br>✓ Sports performance	*N.S;* effect on exercise-induced cardiac troponin T release (p=0.24), changes in antioxidant capacity markers (trolox equivalent antioxidant capacity, uric acid, and malondialdehyde). Markers of inflammation (high-sensitivity C-reactive protein) and exercise-induced skeletal muscle damage (creatine kinase).<br>*No safety concerns were identified in this study.*
Res T. *et al.* 2013 [87]	Randomized, double-blind, placebo-controlled, prospective study	32 trained male cyclists or triathletes (25 ± 1 years, $V^{\cdot}O_{2peak}$ = 60 ± 1 mL·kg⁻¹·min⁻¹, W_{max} = 395 ± 7 W)	0, 20 mg/day	4 weeks	✓ Oxidative Stress (exercise)<br>✓ Sports performance	*N.S*; total plasma antioxidant capacity (p=0.90) or attenuated malondialdehyde levels (p=0.63). Whole-body fat oxidation rates during submaximal exercise (from 0.71 +/- 0.04 to 0.68 ± 0.03 g.min and from 0.66 ± 0.04 to 0.61 ± 0.05 g.min in the Placebo and AX groups, respectively; p=0.73), time trial performance (from 236 ± 9 to 239 ± 7 and from 238 ± 6 to 244 ± 6 W in the Placebo and AX groups, respectively; p = 0.63).<br>*No safety concerns were identified in this study.*
Yagi H. *et al.* 2013 [88]	Case reports	34 OAB patients with anticholinergic agent-resistant (75.5 ± 8.0 years)	0, 12 mg/day*	8 weeks	✓ Overactive bladder (OAB)	Significantly improved on international prostate symptom score (IPSS), QOL scores, benign prostatic hyperplasia impact index (BII) scores and urinary 8-OHdG in patients AX could improve both urinary symptoms and QOL for anticholinergic agent-resistant OAB.<br>*No safety concerns were identified in this study.* [Article in Japanese]
Katagiri M . *et al.* 2012 [89]	Randomized, double-blind, placebo-controlled, prospective study	89 healthy middle-aged and elderly subjects who complained of age-related forgetfulness.	0, 6, 12 mg/day	12 weeks	✓ Brain health (Cognitive functions)	After 12 weeks, CogHealth battery scores improved in the high-dose group (12 mg AX/day). Improved Groton Maze Learning Test scores were seen earlier in the low-dose group (6 mg AX/day) and in the high-dose group than in the placebo group. However, the sample size was too small to show significant differences in cognitive function between the AX and placebo groups.<br>*No safety concerns were identified in this study.*

Kiko T. *et al.* 2012 [90]	Randomized, double-blind, placebo-controlled prospective study	30 healthy subjects (56.3 ± 1.0 yrs.)	0, 6, 12 mg/day	12 weeks	✓ Alzheimer's disease (AD)<br>✓ Oxidative stress (PLOOH in erythrocytes)	Amyloid β (Aβ) 40 and Aβ42 concentrations were much higher in erythrocytes (RBC) than in plasma. RBC Aβ levels increased with aging. After AX supplementation decreased in RBC Aβ concentrations. The RBC Aβ levels were positively correlated with RBC PLOOH, and inversely correlated with AX concentration in RBC.<br>A study related to the ref. [91]<br>*No safety concerns were identified in this study.*
Piermarocchi S. *et al.* 2012 [92]	Randomized, two-arm, prospective study	145 patients with nonexudative (dry) age-related macular degeneration (AMD) (72.5 ± 7 yrs.)	0, 4 mg/days*	24 months	✓ Eye health (AMD)	Two-year results of CARMIS study; *Interventions were vitamin C (180 mg), vitamin E (30 mg), zinc (22.5 mg), copper (1 mg), lutein (10 mg), zeaxanthin (1 mg), and AX (4 mg)* .<br>In the treated group showed stabilization of visual acuity (VA) with significantly (*p*=0.003) better VA scores compared to the non-treated group at 24-month follow-up. An improvement in contrast sensitivity (CS, *p*=0.001) and final mean National Eye Institute visual function questionnaire (NEI VFQ-25) composite scores at 12 and 24 months higher in treated group compared to non-treated group (*p*<0.001).<br>*No safety concerns were identified in this study.*
Macdermid J.C. *et al.* 2012 [93]	Randomized, triple-blind, placebo-controlled prospective study	63 patients with carpal tunnel syndrome	0, 4 mg/days with splinting	9 weeks	✓ Joint health (Carpal tunnel syndrome)	The Symptom Severity Scale (SSS) over the course of treatment in both AX treated and placebo groups (*p*=0.002), but no differences between the groups (*p*=0.18). The Disability of Arm, Shoulder and Hand questionnaire and the Short Form 36-item Health Survey showed no effects over time or between treatment groups.<br>*No safety concerns were identified in this study.*
Saito M. *et al.* 2012 [94]	Randomized, double-blind, placebo-controlled, prospective study	20 healthy subjects	0, 12 mg/day	4 weeks	✓ Eye Health<br>✓ Cardiovascular health (Microcirculatory flow)	Significant increase of the macular square blur rate (SBR) after 4 weeks after AX (*p*=0.018). No statistical difference in the macular SBR was detected in the placebo group (*p*=0.598).<br>*No safety concerns were identified in this study.*
Suganuma K. *et al.* 2012 [95]	Randomized, double-blind, placebo-controlled, prospective study	44 female subjects (Mean 37.26 yrs.)	0, 6 mg/day*	20 weeks	✓ Skin health	Water content (cheeks): increasing trend over the study period. The AX+VC+VE group showed an increase compared to VC+VE (*p*<0.10). Elasticity (upper cheekbones): no significant change. Fine wrinkles: significant improvement in the AX+VC+VE group (also improved compared to VC+VE group)<br>*No safety concerns were identified in this study.*
Tominaga K. *et al.* 2012 [96] (Study 2)	Randomized, double-blind, placebo-controlled, prospective study	36 male subjects (20 to 60 yrs.)	0, 3 mg/ twice a day* (0,6 mg/day)	6 weeks	✓ Skin health	TEWL: significantly decreased in AX (p < 0.01). Water content: increasing trend in the left cheek in the AX group (*p*=0.08). Elasticity: significantly improved in the AX group (*p*<0.05)<br>Fine wrinkles: total area ratio and volume of wrinkles in the AX group decreased (*p*<0.05). Sebum production: decreased in the AX group (*p*=0.085).<br>*No safety concerns were identified in this study.*

Choi H.D. *et al.* 2011 [97]	Randomized, two-arm, prospective study	23 obese and overweight subjects	5 and 20 mg/day	3 weeks	✓ Oxidative Stress (Obesity)<br>✓ Cardiovascular health<br>✓ Metabolic syndrome	5 mg/day: MDA decreased by 34.6%, isoprostane (ISP) decreased by 64.9%, SOD increased by 193%, and TAC increased by 121% after 3 weeks compared to baseline (*p*<0.01). 20 mg/day: MDA decreased by 35.2%, ISP decreased by 64.7%, SOD increased by 194%, and TAC increased by 125% after 3 weeks compared to baseline (*p*<0.01). Decreased LDL cholesterol and ApoB.<br>*No safety concerns were identified in this study.*
Choi, H.D. *et al.* 2011 [98]	Randomized, double-blind, placebo-controlled, prospective study	27 overweight subjects	0, 20 mg/day	12 weeks	✓ Oxidative Stress (Obesity)	MDA reduced by 17.3% and 29% after 8 and 12 weeks compared to placebo (*p*<0.01), ISP reduced by 40.2% and 52.9% after 8 and 12 weeks compared to placebo (*p*<0.01), SOD increased by 124.8% after 12 weeks compared to placebo (*p*<0.01), and TAC increased by 130.1% after 12 weeks compared to placebo (*p*<0.05).<br>*No safety concerns were identified in this study.*
Djordjevic B. *et al.* 2011 [84]	Randomized, double-blind, placebo-controlled, prospective study	32 male elite soccer players	0, 4 mg/day	90 days	✓ Oxidative Stress (exercise)<br>✓ Sports performance	Changes in elevated O2-⁻ concentrations after soccer exercise were statistically significant only in the Placebo group (exercise × supplementation effect, *p*<0.05); TAS values decreased significantly only in the Placebo group after exercise (*p*<0.01). After intervention, total SH group content increased (21% and 9%, respectively), and the effect of AX was marginally significant (*p*=0.08). Basal SOD activity was significantly reduced in both the Placobo and AX groups at the end of the study (main training effect, P<0.01).Post-exercise CK and AST levels were significantly lower in the AX group than in the Placebo group (*p*<0.05)<br>*No safety concerns were identified in this study.*
Earnest C.P. *et al.* 2011 [99]	Randomized, double-blind, placebo-controlled, prospective study	14 amateur endurance-trained subjects (18-39 years, $\dot{V}O_{2peak}$ = 52.84 ± 3.5 mL·kg⁻¹·min⁻¹, W_{max} = 330 ± 26 W	0, 4 mg/day	28 days	✓ Sports performance	Improved performance in the 20-km cycling time trial in the AX group (n=7, -121 s; 95% CI, -185, -53), but not in the Placebo group (n=7, -19 s; 95% CI, -84, 45). AX group significantly increased power output (20 W; 95% CI, 1, 38), while the Placebo group did not (1.6 W; 95% CI, -17, 20). N.S: carbohydrate, fat oxidation and blood indices indicative of fuel mobilization.<br>*No safety concerns were identified in this study.*
Hashimoto H. *et al.* 2011 [23]	Open-labeled, prospective study	35 subjects who underwent bilateral cataract surgery (intraocular lens implantation) (Mean *c.a* 71 yrs.)	6 mg/day	2 weeks	✓ Eye health<br>✓ Oxidative Stress (in aqueous humor of cataract patients)	Reduced total hydroperoxides (hydrogen peroxides, lipid peroxides, and peroxides of protein in aqueous humor; *p*<0.05)<br>*No safety concerns were identified in this study.* [Article in Japanese]
Kim, J.H. *et al.* 2011 [100]	Randomized, repeated measured, prospective study	39 heavy smokers, 39 non-smokers	0, 5, 20, or 40 mg/day	3 weeks	✓ Oxidative Stress (smoking)	5 mg/day: MDA and ISP significantly lower after 2 and 3 weeks compared to baseline in smokers (*p*<0.05). SOD and TAC significant increase after 1, 2, and 3 weeks compared to baseline in smokers (*p*<0.05) 20 mg/day: MDA and ISP significantly lower after 1, 2, and 3 weeks compared to baseline in smokers (*p*<0.05). SOD and TAC significant increase after 1, 2, and 3 weeks compared to baseline in smokers (*p*<0.05). 40 mg/day: MDA and ISP significantly lower after 1, 2, and 3

							weeks compared to baseline in smokers (*p*<0.05). SOD and TAC significant increase after 2 and 3 weeks compared to baseline in smokers (*p*<0.05)<br>*No safety concerns were identified in this study.*
Miyazawa T. *et al.* 2011 [101]	Randomized, double-blind, placebo-controlled, prospective study	30 middle-aged & senior subjects (mean: 50.6 yrs.)	0, 1, 3 mg/day	12 weeks	✓	Bioavailability, ADME	Erythrocyte AX concentrations after 4 or 12 weeks of supplementation (3 mg/day administration, 2.5nM AX in packed cell for 4weeks, 2.9nM for 4weeks respectively) were significantly higher than after placebo or 1 mg AX supplementation. No significant changes in erythrocyte carotenoid composition or phospholipid hydroperoxide concentration after astaxanthin intake in either the 1 mg/day or 3 mg/day groups.<br>*No safety concerns were identified in this study.*
Miyazawa T. *et al.* 2011 [102]	Randomized, double-blind, placebo-controlled, prospective study	20 middle-aged & senior subjects (mean: 50.6 yrs.)	1, 3 mg/day	12 weeks	✓	Bioavailability, ADME	Plasma AX concentrations were significantly elevated in a dose-dependent manner after AX supplementation than before in both the 1 mg/day (0.4, 12.3 and 18.9 nM for 0, 4 and 12 weeks after administration respectively) and 3 mg/day (0.7, 14.4 and 62.4 nM for 0, 4 and 12 weeks after administration respectively) groups.<br>*No safety concerns were identified in this study.*
Nakagawa K. *et al.* 2011 [91]	Randomized, double-blind, placebo-controlled prospective study	30 healthy subjects	0, 6, 12 mg/day	12 weeks	✓<br>✓	Oxidative Stress<br>Bioavailability, ADME	6mg/day: reduction in total phospholipid hydroperoxides (PLOOH) after 12 weeks compared to baseline (*p*<0.01) and compared to placebo (*p*<0.05). Reduced phosphatidyl-<br>ethanolamine hydroperoxide (PEOOH) after 12 weeks compared to baseline (*p*<0.05) and compared to placebo (*p*<0.05). Increased AX concentration in plasma after 12 weeks (86 nM) compared to baseline (*p*<0.01, 6 to 9 nM) and compared to placebo (*p*<0.01, 8 nM). 12mg/day: 48% reduction in total PLOOH after 12 weeks compared to baseline (*p*<0.01) and 35% less total PLOOH at 12 weeks compared to the control group (*p*<0.05). The 12mg/day group had 46% less phosphatidylcholine hydroperoxide (PCOOH) at 12 weeks compared to baseline (*p*<0.01). Increased AX concentration in plasma after 12 weeks (109 nM) compared to baseline (*p*<0.01, 8 to 9 nM) and compared to placebo (*p*<0.01, 8 nM)<br>*No safety concerns were identified in this study.*
Peng L. *et al.*, 2011 [103]	Randomized, placebo-controlled study	115 healthy subjects	0, 40 mg/day	90 days	✓	Oxidative Stress	Comparing with the control group, MDA contents in the test group decreased significantly (*p*<0.01), and SOD and GSH-Px activities increased significantly (*p*<0.01).<br>*No safety concerns were identified in this study.*

Satoh A. *et al.* 2011 [104]	Randomized, single-blind, placebo-controlled prospective study	26 healthy subjects	0, 3 mg/day	4+4 weeks	✓	Skin health	After 4 weeks of administration, UV light was irradiated (2 MED) and the given test substance was administered for another 4 weeks. Skin color was evaluated with a colorimeter, a mexameter, and a skin color scale before administration, before UV irradiation, and 1, 7, 14, 21, and 28 days after UV irradiation. The results showed that the L value of the colorimeter and skin color scale scores were significantly higher in the Ax group than in the placebo group, and the amount of melanin after UV irradiation was significantly lower in the Ax group than in the placebo group.<br>*No safety concerns were identified in this study.* [Article in Japanese]
Nagaki Y. *et al.* 2010 [105]	Randomized, single-blind, placebo-controlled prospective study	82 healthy subjects with VDT operation (6 h or more per day for more than 1 year) and frequently experienced eyestrain	0, 9 mg/day	4 weeks	✓	Eye health (Eyestrain)	(1) The post-treatment accommodation ability of the AX group with respect to value and rate of change was significantly higher than that of the control group. (2) The distribution of rate of change also showed significant improvement in post-treatment accommodation ability of the AX group when compared to control group. (3) Subjective questionnaire regarding 4 conditions ("eyestrain", "hazy vision", "flickering images", "my shoulders/back feel stiff"), showed that AX group significantly improved to those of control group.<br>*No safety concerns were identified in this study.* [Article in Japanese]
Park J.S. *et al.* 2010 [106]	Randomized, double-blind, placebo-controlled, prospective study	42 healthy subjects	0, 2, 8 mg/day	8 weeks	✓<br>✓<br>✓	Oxidative Stress<br>Immunity<br>Bioavailability, ADME	Plasma AX concentration<br>From 4 weeks after administration, *c.a.* 0.1 μM (2 mg AX/day), *c.a.* 0.12-0.14 μM (8 mg AX/day)<br>Oxidative Stress<br>2 mg/day: Concentrations of plasma 8-hydroxy-2′-deoxyguanosine reduced after 4 weeks and 8 weeks compared to placebo (p<0.05). 8 mg/day: Concentrations of plasma 8-hydroxy-2′-deoxyguanosine reduced after 4 weeks and 8 weeks compared to placebo (p<0.05)<br>Immunity<br>Increased natural killer (NK) cell cytotoxic activity, and increased total T and B cell subpopulations, but did not influence populations of T_h, $T_{cytotoxic}$ or NK cells. No difference in TNFα and IL-2 concentrations, but plasma IFN-γ and IL-6 increased on 8 week in subjects given 8 mg AX.<br>*No safety concerns were identified in this study.*
Yamada T. *et al.* 2010 [107]	Open-labeled, prospective study	6 healthy subjects and 6 Sjoegren's syndrome (SS) subjects	12 mg/day	2 weeks	✓<br>✓	Oxidative Stress<br>Immunity	Although increased amount of salivary secretion after the intake of AX for 2 weeks was faint in the SS group (1.02 g/2 min to 1.04 g/2 min, p = 0.69), a significant increase was also observed in the normal group (6.23 g/2 min to 7.02 g/2 min, p = 0.03). reduced protein oxidation (-10%, p<0.05)<br>*No safety concerns were identified in this study.*

Yoshida H. *et al.* 2010 [108]	Randomized, double-blind, placebo-controlled, prospective study	61 non-obese subjects with fasting serum triglyceride of 120-200mg/dl and without diabetes and hypertension	0,6,12,18 mg/day	12 weeks	✓ Cardiovascular health<br>✓ Metabolic syndrome	Multiple comparison: triglycerides were significantly decreased by 12 and 18 mg/day and HDL-cholesterol was significantly increased by 6 and 12 mg. Serum adiponectin was increased by AX (12 and 18 mg/day), and changes in adiponectin were positively correlated with changes in HDL-cholesterol.<br>*No safety concerns were identified in this study.*
Iwabayashi M. *et al.* 2009 [109]	Open-labeled, prospective study	35 healthy female subjects (with high oxidative stress, postmenopausal)	12 mg/day	8 weeks	✓ Oxidative stress<br>✓ Metabolic syndrome<br>✓ Mood/Stress (unidentified complaints)	Increased blood biological antioxidant potential (Biological Antioxidant Potential (BAP); +4.6%, $p<0.05$). After eight-week treatment with astaxanthin, significant improvement was observed in 5 of 34 physical symptoms listed in the common questionnaire, including "tired eyes", "stiff shoulders", "constipation", "gray hair", and "cold skin", and in 3 of 21 mental symptoms, including "daily life is not enjoyable", "difficulty in falling asleep", and "a sense of tension". In addition, systolic ($p=0.021$) and diastolic blood pressure ($p<0.001$) significantly decreased.<br>*No safety concerns were identified in this study.*
Kajita M *et al.* 2009 [110]	Open-labeled, prospective study	82 healthy males with presbyopia (Mean 53.9 yrs.)	6 mg/day	4 weeks	✓ Eye health (Eyestrain)	The pupillary constriction ratio before and after AX supplementation was measured by TriIRIS C9000. The change in subjective symptoms after supplementation was examined by a questionnaire. The results showed a significant increase in pupillary constriction ratio after supplementation of AX, therefore suggesting that astaxanthin may also improve the accommodation function of the eye and some subjective symptoms related to presbyopia in middle-aged and older people with complaints of eye strain.<br>*No safety concerns were identified in this study.* [Article in Japanese]
Okada Y. *et al.* 2009 [111]	Open-labeled, prospective study	Healthy subjects; before the meal to nonsmokers (n=7), after the meal to nonsmokers (n=6), and after the meal to smokers (n=7)	48 mg	Single dosing	✓ Bioavailability, ADME	Dosing timing significantly affected AX bioavailability, including area under the curve ($AUC_{0\text{-}168h}$, 2,968 ± 959 μg·h/L in the pre-meal group and 7,219 ± 3,118 μg·h/L in the after-meal group), suggesting a higher availability in the after-meal group. Smoking also affected pharmacokinetic parameters, significantly reducing the elimination half-life (t1⁄2) of AX.<br>*No safety concerns were identified in this study.*
Satoh A. *et al.* 2009 [112]	Open-labeled, prospective study	20 subjects at risk for developing metabolic syndrome (from 127 healthy subjects)	4, (8, 20) mg/day	4 weeks	✓ Cardiovascular health<br>✓ Metabolic syndrome	When subjects who met the diagnostic criteria for metabolic syndrome in Japan (SBP≥130mmHg, DBP≥85mmHg, TG≥150mg/dl, FG≥100mg/dl) at the start of the study were selected fron 4mg group, significant decreased in SBP ($p<0.01$). On the other hand, there was no significant decrease in DBP. Reduced TG after treatment (218 mg/dl) than the baseline value (292 mg/dl), marginally reduced fasting glucose after the intervention ($p<0.1$).<br>*No safety concerns were identified in this study*

Satoh A. *et al.* 2009 [112]	Open-labeled, prospective study	10 healthy male subjects (50–69 yrs.)	12 mg/day	12 weeks	✓ Cognitive function	In the CogHealth tasks (simple response, choice response, working memory, delayed memory, and divided attention), significant reductions in reaction time were observed in the "divided attention" task after 6 weeks and in all tasks after 12 weeks. Accuracy on the "working memory" task was significantly improved after 12 weeks of treatment, but no such effect was observed on the "delayed recall" task. *No safety concerns were identified in this study*
Satoh A. *et al.* 2009 [113]	Randomized, single-blind, placebo-controlled prospective study	27 patients with atopic dermatitis	0, 12 mg/day	4 weeks.	✓ Oxidative stress<br>✓ Skin health<br>✓ Atopic dermatitis<br>✓ Immunity	Severity (SCORAD), pruritus (VAS), quality of life (Skindex-16, STAI), immune function (Th1/Th2, blood catecholamines) and antioxidant status (urinary 8-OHdG, isoprostanes) were assessed. There were significant differences in the degree of itching between the Ax and placebo groups. However, there was significant improvement in Skindex-16 symptoms and STAI status anxiety in the Ax group. In addition, the Th1/Th2 balance shifted significantly toward Th1, and urinary 8-OHdG concentrations decreased slightly but significantly. *No safety concerns were identified in this study* [Article in Japanese]
Seya Y. *et al.* 2009 [114]	Open-labeled, prospective study	10 healthy subjects with VDT operation (Mean Age 24.6 yrs., VDT 6.9 h/day)	6 mg/day	4 weeks.	✓ Eye health<br>✓ (Eyestrain)	The effects of visual fatigue on reaction times measured in a visual pursuit task. Regardless of the duration of the intake period for AX, became shorter the reaction times at the early trials/blocks of the reaction time task than those at the late trials/blocks during a long lasting, 500 trial experimental session. I addition, shorter the reaction times at the late stages (14th and 28th day) of the AX intake than those at the early stage (1st day). *No safety concerns were identified in this study* [Article in Japanese]
Kupcinskas L. *et al.* 2008 [115]	Randomized, single-blind, placebo-controlled prospective study	131 patients with functional dyspepsia with or without *Helicobacter pylori* infection	0, 8, 20 mg/ twice a day (0,16, 40 mg/day)	4 weeks	✓ GI health	No statistically significant differences were observed between the three treatment groups at the end of treatment (week 4) for mean scores on the Gastrointestinal Symptom Rating Scale (GSRS) for abdominal pain, indigestion, and reflux syndrome, and similar results were observed at the end of follow-up (week 8). The reflux syndrome tended to improve at the higher dose (40 mg) compared to the other treatment groups (16 mg and placebo,) and this response was more pronounced (p=0.04) in *H.pylori*-infected patients. *No safety concerns were identified in this study.*
Malmstena C.L.L. *et al.* 2008 [116]	Randomized, double-blind, placebo-controlled prospective study	40 young healthy subjects (17-19 years)	0, 4 mg/day	3 months	✓ Sports performance	Increased average number of knee bending (squats) increased by 27.05 (from 49.32 to 76.37, AX group) vs. 9.0 (from 46.06 to 55.06, placebo subjects), p = 0.047. *No safety concerns were identified in this study.*

Miyawaki H. *et al.* 2008 [117]	Single-blind, placebo-controlled prospective study	20 healthy subjects	0, 6 mg/days	10 days	✓ Cardiovascular health (Microcirculatory flow)	The time for blood to pass through the microchannel array flow analyzer decreased from 52.8 ± 4.9 seconds to 47.6 ± 4.2 seconds in the AX group (*p*<0.01), and a significant difference was found when comparing values in the AX group (47.6 ± 4.2 seconds) and placebo group (54.2 ± 6.7 seconds) (*p*<0.05). *No safety concerns were identified in this study.*
Parisi V.. *et al.* 2008 [118]	Randomized, two-arm, prospective study	27 patients with nonadvanced AMD and visual acuity ≥0.2 logarithm of the minimum angle of resolution (69.4 ± 4.3 yrs.)	0, 4 mg/days*	12 months	✓ Eye health (AMD)	one-year results of CARMIS study; *Interventions were vitamin C (180 mg), vitamin E (30 mg), zinc (22.5 mg), copper (1 mg), lutein (10 mg), zeaxanthin (1 mg), and AX (4 mg)* . In nonadvanced AMD eyes, selective dysfunction of the central retina (0°-5°) was ameliorated by carotenoid and antioxidant supplementation. There were no functional changes in the more peripheral (5°-20°) retina. *No safety concerns were identified in this study.*
Tsukahara H. *et al.* 2008 [119]	Open-labeled, prospective study	13 healthy subjects with shoulder Stiffness	6 mg/days*	4 weeks	✓ Fatigue (Shoulder Stiffness)<br>✓ Cardiovascular health<br>✓ EyeHealth<br>✓ Microcirculatory flow	*6mg of AX and 50mg of flaxseed lignin.* All patients completed the efficacy evaluation, which confirmed significant improvement in physical symptoms such as shoulder stiffness, physical fatigue, mental irritability, cold hands and feet, eye fatigue, and redness of the eyes. At the end of the treatment, laser Doppler graphics also confirmed a significant increase in blood flow in the shoulders. *No safety concerns were identified in this study.* [Article in Japanese]
Uchiyama A. *et al.* 2008 [120]	Open-labeled, prospective study	17 subjects at risk for developing metabolic syndrome	8 mg twice day	3 months	✓ Cardiovascular health<br>✓ Metabolic syndrome<br>✓ Bioavailability, ADME	Significant decreases plasma HbAlc (*p*=0.0433) and TNF-a levels (*p*=0.0022) and increase adiponectin concentration (*p*=0.0053). *N.S*: body weight, BMI and waist circumference. The blood concentration reached the plateau after a month treatment and retained at that level unti1 3 months of the treatment (0.2-0.25μg/mL (0.34-0.42μmol/L)). *No safety concerns were identified in this study.*
Andersen LP. *et al.* 2007 [121]	Randomized, double-blind, placebo-controlled, prospective study	44 patients with functional dyspepsia with or without *Helicobacter pylori* infection	0, 20 mg/ twice a day (0,40 mg/day)	4 weeks	✓ GI health	There were no significant changes in either H. pylori density or interleukins during or after treatment; significant upregulation of CD4 (*p*<0.05) and downregulation of CD8 (*p*<0.001) was observed in *H. pylori* patients treated with AX. *No safety concerns were identified in this study.*
Fukamauchi M. *et al.* 2007 [122]	Randomized, double-blind, placebo-controlled, prospective study	32 healthy subjects	0, 6 mg/day	6 weeks	✓ Sports performance<br>✓ Weight loss	Synergistic effects of AX intake (12 mg/day, 6 weeks) and aerobic exercise (walking) were studied. AX contributed to reduction of body fat and suppressed the increase in blood lactate level after exercise. *No safety concerns were identified in this study.* [Article in Japanese]

Karppi, J. *et al.* 2007 [123]	Randomized, double-blind, placebo-controlled, prospective study	39 healthy subjects	0, 8 mg/day	3 months	✓<br>✓	Oxidative Stress<br>Bioavailability, ADME	Decreased oxidation of fatty acids in healthy men (p<0.05) AX supplementation elevated plasma AX levels to 0.032 nM (*c.a.* 0.02μg/mL, p < 0.001 for the change compared with the placebo group)<br><br>*No safety concerns were identified in this study.*
Iwasaki T. *et al.* 2006 [124]	Randomized, double-blind, placebo-controlled crossover study	39 healthy females (Mea 20.5 yrs.)	0, 6 mg/day	2 weeks	✓	Eye health (Eyestrain)	Accommodative function and subjective symptoms relating to eyestrain were measured before and after the task, and after the 10-minute rest following the task. The data were then compared between AX and Placebo groups by the double-blind cross-over method. After the task, accommodation contraction and relaxation times were extended in both the AX and Placebo groups. Comparison between the two groups showed that after the task, accommodation relaxation time was significantly extended in the Placebo group, in contrast to AX. Accommodative contraction and relaxation times were significantly prolonged after the 10-minute rest in the P group as compared to AX. The symptoms eye fatigue, eye heaviness, blurred vision and eye dryness in the Placebo group were increased, but the AX group only showed increases in eye fatigue and eye heaviness.<br>*No safety concerns were identified in this study.* [Article in Japanese]
Nagaki Y. *et al.* 2006 [125]	Randomized, double-blind, placebo-controlled, prospective study	48 healthy subjects with VDT operation (6 h or more per day for more than 1 year) and frequently experienced eyestrain	0, 6 mg/day	4 weeks	✓	Eye health (Eyestrain)	1. Significant improved the magnitude of change of amplitude of accommodation before and after supplementation in the AX supplemented group compared with the control group.<br>2. Significantly better scores of the distribution of the percentage change in amplitude of accommodation after supplementation in the AX supplemented group compared with the control group.<br>3. In the subjective asthenopia evaluation, significantly improved for the two items "dimness of sight" and "stiff shoulders and back" the AX supplemented group compared with the control group, and an improvement tendency was seen in "heavy head".<br>*No safety concerns were identified in this study.* [Article in Japanese]
Yamashita E. 2006 [126]	Randomized, single-blind, placebo-controlled, prospective study	49 female subjects (Mean 47 yrs.)	0, 2 mg/ twice a day (0,4 mg/day)	6 weeks	✓	Skin health	Water content (left cheek): significant improvement in the 6-week treatment group (compared to the start of treatment). Elasticity (left eye rim): significant improvement in the placebo group at weeks 3 and 6 (p<0.05) Inspection/Palpation by dermatologist: improvement in fine lines, wrinkles and elasticity (week 6) Skin surface observation: improvement in fine lines, wrinkles and elasticity (week 6)<br>*No safety concerns were identified in this study.*

Bloomer, R.J. *et al.* 2005 [127]	Randomized, placebo-controlled, prospective study	20 resistance trained male subjects (25.1 ± 1.6 years)	0, 4 mg/day*	3 months	✓ Oxidative Stress (exercise)<br>✓ Sports performance	*N.S*; Muscle soreness, creatine kinase (CK), and muscle performance was measured before and through 96 h post- eccentric exercise<br><br>*No safety concerns were identified in this study.*
Comhaire F.H. *et al.* 2005 [128]	Randomized, double-blind, placebo-controlled, prospective study	30 males with infertility of ≥ 12 months	0, 16 mg/day	3 months	✓ Infertility<br>✓ Oxidative stress	Significantly decreased ROS and Inhibin B and sperm linear velocity increased in the Astaxanthin group (*n*=11), but not in the placebo group (*n*=19). The total and per cycle pregnancy rates among Astaxanthin group (54.5 % and 23.1 %) were higher compared with 10.5 % and 3.6 % respectively in the placebo cases(p=0.028; p=0.036).<br>*No safety concerns were identified in this study.*
Nagaki Y. *et al.* 2005 [129]	Randomized, placebo-controlled, prospective study	36 health subjects (*c.a.* 41 yrs.)	0, 6 mg/day	4 weeks	✓ Eye Health<br>✓ ADME<br>✓ Cardiovascular health (Microcirculatory flow)	The fasting plasma AX level in the AX group was significantly (p<0.001, 35.6 ng/mL at 4 weeks) higher than before supplementation. The fasting plasma AX level in the placebo group after placebo treatment remained unchanged. After 4 weeks supplementation, retinal capillary blood flow in the AX group was significantly (p<0.01) higher than before supplementation in both eyes, while retinal capillary blood flow in the placebo group after placebo treatment was unchanged. Intraocular pressures in both groups remained unchanged during the supplementation period.<br>*No safety concerns were identified in this study.* [Article in Japanese]
Nitta T. *et al.* 2005 [130]	Randomized, placebo-controlled, prospective study	30 health subjects (Mean time consumed for close work (e.g., VDT work) was approx. 7 h./day)	0, 6, 12 mg/day	4 weeks	✓ Eye health (Eyestrain)	l. Significantly increased the objective accommodation power of the AX 12 mg group compared to that of pre-dosing.<br>2. Significantly shortened the positive accommodation time was in the AX 6 mg and the 12 mg groups compared to those of pre-dosing, and negative accommodation time was significantly shortened in the AX placebo and the 6 mg groups compared to those of pre-dosing.<br>3. VAS; many parameters in subjective symptoms were improved in the AX 6 mg group.<br>*No safety concerns were identified in this study.* [Article in Japanese]
Shiratori K. *et al.* 2005 [131]	Randomized, placebo-controlled, prospective study	39 healthy subjects who complained of eyestrain	0, 6 mg/day	4 weeks	✓ Eye health (Eyestrain)	1. Significantly higher sub-objective accommodation power (changing rate) in the AX group than that of the control group.<br>2. Significantly higher rate of positive and negative accommodation times (rate of change) in the AX groupcompared to those of the control group.<br>3. In the AX group, subjective degree of asthenopia (eye strain) measured by VAS showed significant improvement in two parameters, i.e., “My eyes get bleary” and “I get irritated easily” than the control group.<br>*No safety concerns were identified in this study.* [Article in Japanese]

Takahashi N. *et al.* 2005 [132]	Open-labeled, prospective study	10 healthy subjects	6 mg/day	2 weeks	✓ Eye health (Eyestrain)	Effects of astaxanthin on accommodative recovery derived from a rest after VDT work were studied. Evaluated (9 dominant eyes) by values for objective diopter, HFC (High Frequency Component in Accommodative micro-fluctuation) and accommodative reaction. Increased HFC after rest was significantly restrained by AX supplementation compared to the increase shortly after working. *No safety concerns were identified in this study.* [Article in Japanese]
Coral-Hinostroza G.N. *et al.* 2004 [133]	Open-labeled, prospective study	3 male subjects (41-50 yrs, BMI 27.7-27.8)	10, 100 mg	Single dose	✓ ADME (*R/S, E/Z,* esters)	*Test meal: Dissolved the oily AX diesters in warm olive oil (30% of meal weight) and served as a dressing to a pasta salad.* 100 mg (C_{max}: 0.28±0.1 mg/L, T_{max}: 11.5±1.2 hr, $T_{1/2}$: 52.2±39.5 hr. $AUC_{(0-\infty)}$ 11.0±2.8mg h/L) C_{max} at the low dose (10 mg): 0.08 mg/l (non-linear dose response). AX esters were not detected in plasma. *No safety concerns were identified in this study*
Kim Y.K.. *et al.* 2004 [134]	Open-labeled, prospective study	15 healthy postmenopausal females	0, 2, 8 mg/day	8 weeks	✓ Oxidative Stress ✓ Metabolic syndrome (postmenopausal females)	Decreased plasma TBARS levels: 2 mg group from 1.42±0.18 to 1.13±0.18 nM/mg (*p*<0.05). 8 mg AX group from 1.62±0.14nM/mg to 1.13±0.12nM/mg after 8 weeks (*p*<0.05). Increased TAS from 0.85±0.42 mM/l to 1.90±0.58 mM/l in the 8 mg group. Urinary 8-isoprostanes excretion did not decrease significantly. Increase HDL-cholesterol levels in 2mg and 8mg group increased significantly after 8 weeks from 50.6±5.8 to 60.4±7.1mg/dl, 44.4±10.7 to 49.4±2.7mg/dl respectively (*p*<0.05). In the 2mg group, triglyceride decreased significantly from 171.6±67.4 mg/dl to 145.8±5.1mg/dl (*p*<0.05) *No safety concerns were identified in this study.*
Nakamura A. *et al.* 2004 [135]	Randomized, placebo-controlled, prospective study	49 healthy subjects	0, 2, 4, 12 mg/day	4 weeks	✓ Eye health (Eyestrain)	For far visual acuity (5 m), no significant difference in the results for uncorrected visual acuity of the 0 mg group and 2 mg group before administration was begun and after the start of peroral administration, but uncorrected visual acuity improved significantly for the 4mg group and 12mg group (*p*<0.05). For corrected visual acuity, no significant difference was found for any of the groups. No significant changes were found in refraction or flicker fusion frequency. For the accommodation test, positive accommodation time was shortened significantly for the 4mg group and 12 mg group (*p*<0.05). No significant change was found in the other items. For pupillary reflex, no significant difference was found in the miosis ratio (%), T1 (ms), T2 (ms) or VC (mm2/s). *No safety concerns were identified in this study.* [Article in Japanese]

Tajima T. *et al.* 2004 [136]	Randomized, double-blind, placebo-controlled, crossover study	18 healthy subjects (35.7 ± 4 years)	0, 5 mg/day	2 weeks	✓ Sports performance	Increased in CV_{RR} and HF/TF (Heart rate variability) were significant during exercise at 70% maximum heart rate (HRmax) intensity ($p<0.05$). Also, after the AX supplementation, decreased minute ventilation (V_E) during exercise at 70% HRmax ($p<0.05$). Decreased LDL cholesterol ($p<0.05$) and respiratory quotient after exercise.<br>*No safety concerns were identified in this study.* [Article in Japanese]
Mercke Odeberg J. et al. 2003 [137]	Open-labeled, prospective study	32 healthy male Subjects (nonsmoker/no medications)	40 mg	Single dose	✓ ADME (Lipid formulation)	*Test meal:* Haematococcus *algal meal and dextrin in hard gelatin capsules (reference), and with long-chain triglyceride (palm oil) and polysorbate 80 (formulation A), glycerol mono- and dioleate and polysorbate 80 (formulation B), and glycerol mono- and dioleate, polysorbate 80 and sorbitan monooleate (formulation C).* The highest bioavailability was observed with formulation B (3.7 times higher $UC_{(0-\infty)}$ compared to reference control)<br>*No safety concerns were identified in this study.*
Nagaki Y. *et al.* 2002 [138]	Randomized, placebo-controlled, prospective study	26 VDT subjects + 13 non-VDT subjects (control)	0, 5 mg/day	4 weeks	✓ Eye health (Eyestrain)	Group A: 13 non-VDT workers/no supplementation. Group B: 13 VDT workers with AX, 5 mg/day, for 4 weeks, Group C: 13 VDT workers with placebo, 5 mg/day, for 4 weeks.<br>Accommodation amplitudes in Groups B and C before supplementation were significantly ($p<0.05$) lower than in Group A. After AX supplementation, the accommodation amplitude in Group B was significantly ($p<0.01$) larger than before supplementation, while accommodation amplitude in Group C after placebo supplementation was unchanged. The CFFs in Groups B and C before supplementation were significantly ($p<0.05$) lower than in Group A. The CCFs in Groups B and C did not change after supplementation. Amplitudes and latencies of P100 in PVEP in Groups B and C before supplementation were similar to those in Group A and did not change after supplementation.<br>*No safety concerns were identified in this study.*
Sawaki *et al.* 2002 [139]	Randomized, double-blind, placebo-controlled, prospective study	18 healthy male Subjects (College handball player)	0, 6 mg/day	4 weeks	✓ Sports performance (Muscle fatigue)<br>✓ Eye health (Eyestrain)	No changes in static and dynamic visual acuity measurements were observed before and after administration of AX. Regarding the deep vision measurements after administration of AX, the AX group showed better values compared to the placebo group. Flicker values after AX administration showed that visual acuity was significantly more acute in the AX group than in the placebo group.Although there was no difference between the two groups in post-exercise CK levels or heart rate, the AX group had significantly lower blood lactate levels 2 minutes after exercise.<br>*No safety concerns were identified in this study.* [Article in Japanese]

Yamashita E. 2002 [140]	Randomized, double-blind, placebo-controlled, prospective study	16 healthy female subjects with dry skin	0, 2 mg/day*	4 weeks	✓	Skin health	Moisture content: trend of improvement in the AX group compared to placebo at week 2 on the left eye corner and at week 4 on the cheeks, with significant improvement at week 4 on the eye corner (p<0.05). Wrinkle depth (eye corner): No significant difference. Subjective symptoms (questionnaire): subjective improvement in spots/freckles (week 2), acne/wipes (week 4). Inspection/Palpation by dermatologist: Improvement in smoothness, moistness and firmness. *No safety concerns were identified in this study.* [Article in Japanese]
Østerlie M. *et al.* 2000 [141]	Open-labeled, prospective study	3 male subjects (37-43 years, BMI 27.5-31.7)	100 mg	Single dose	✓	ADME (*R*/*S*, *E*/*Z*)	*Test meal: prepared by dispersing the AX beadlets in warm water (40°C) and mixing with olive oil (50% of meal weight) and cereals.* C_{max}: 1.3±0.1 mg/L , Tmax: 6.7±1.2 hr, $T_{1/2}$: 21±11 hr, $AUC_{(0-\infty)}$ 42±3 mg h/L. Accumulated 13*Z*-AX selectively, whereas the steroisomers distribution was similar to that of the experimental meal. AX was present mainly in VLDL containing chylomicrons (36-64% of total AX), whereas LDL and HDL contained 29% and 24% of total AX, respectively. The AX isomer distribution in plasma, VLDL/CM, LDL, and HDL was not affected by time. *No safety concerns were identified in this study.*

This table was updated and prepared from Ref. [142] to May 2023. For statistical analysis of efficacy, most analyses were performed with "Per Protocol Set," thus the number of subjects adopted for the final analysis is shown.

† With the exception of the study in which safety was the only primary outcome, the other outcomes were noted.

* In addition to AX, other nutrients such as antioxidants were used in the study.

# All the studies in the "Dose" column that did not indicate the origin were conducted using AX derived from *Haematococcus* algae.

AX concentrations were shown as free form.

References

1. Barker, G.A.; Parten, A.L.; Lara, D.A.; Hannon, K.E.; McAllister, M.J.; Waldman, H.S. Astaxanthin Supplementation Reduces Subjective Markers of Muscle Soreness following Eccentric Exercise in Resistance-Trained Men. *Muscles* **2023**, *2*, 228-237.
2. Ciaraldi, T.P.; Boeder, S.C.; Mudaliar, S.R.; Giovannetti, E.R.; Henry, R.R.; Pettus, J.H. Astaxanthin, a natural antioxidant, lowers cholesterol and markers of cardiovascular risk in individuals with prediabetes and dyslipidaemia. *Diabetes Obes Metab* **2023**, doi:10.1111/dom.15070.
3. Heidari, M.; Chaboksafar, M.; Alizadeh, M.; Sohrabi, B.; Kheirouri, S. Effects of Astaxanthin supplementation on selected metabolic parameters, anthropometric indices, Sirtuin1 and TNF-alpha levels in patients with coronary artery disease: A randomized, double-blind, placebo-controlled clinical trial. *Front Nutr* **2023**, *10*, 1104169, doi:10.3389/fnut.2023.1104169.
4. Jabarpour, M.; Aleyasin, A.; Nashtaei, M.S.; Lotfi, S.; Amidi, F. Astaxanthin treatment ameliorates ER stress in polycystic ovary syndrome patients: a randomized clinical trial. *Sci Rep* **2023**, *13*, 3376, doi:10.1038/s41598-023-28956-8.
5. Nieman, D.C.; Woo, J.; Sakaguchi, C.A.; Omar, A.M.; Tang, Y.; Davis, K.; Pecorelli, A.; Valacchi, G.; Zhang, Q. Astaxanthin supplementation counters exercise-induced decreases in immune-related plasma proteins. *Front Nutr* **2023**, *10*, 1143385, doi:10.3389/fnut.2023.1143385.
6. Rostami, S.; Alyasin, A.; Saedi, M.; Nekoonam, S.; Khodarahmian, M.; Moeini, A.; Amidi, F. Astaxanthin ameliorates inflammation, oxidative stress, and reproductive outcomes in endometriosis patients undergoing assisted reproduction: A randomized, triple-blind placebo-controlled clinical trial. *Front Endocrinol (Lausanne)* **2023**, *14*, 1144323, doi:10.3389/fendo.2023.1144323.
7. Saeidi, A.; Nouri-Habashi, A.; Razi, O.; Ataeinosrat, A.; Rahmani, H.; Mollabashi, S.S.; Bagherzadeh-Rahmani, B.; Aghdam, S.M.; Khalajzadeh, L.; Al Kiyumi, M.H.; et al. Astaxanthin Supplemented with High-Intensity Functional Training Decreases Adipokines Levels and Cardiovascular Risk Factors in Men with Obesity. *Nutrients* **2023**, *15*, doi:10.3390/nu15020286.
8. Sekikawa, T.; Kizawa, Y.; Li, Y.; Miura, N. Effects of diet containing astaxanthin on visual function in healthy individuals: a randomized, double-blind, placebo-controlled, parallel study. *J Clin Biochem Nutr* **2023**, *72*, 74-81, doi:10.3164/jcbn.22-65.
9. Yoshida, K.; Sakai, O.; Honda, T.; Kikuya, T.; Takeda, R.; Sawabe, A.; Inaba, M.; Koike, C. Effects of Astaxanthin, Lutein, and Zeaxanthin on Eye-Hand Coordination and Smooth-Pursuit Eye Movement after Visual Display Terminal Operation in Healthy Subjects: A Randomized, Double-Blind Placebo-Controlled Intergroup Trial. *Nutrients* **2023**, *15*, doi:10.3390/nu15061459.
10. Waldman, H.S.; Bryant, A.R.; Parten, A.L.; Grozier, C.D.; McAllister, M.J. Astaxanthin Supplementation Does Not Affect Markers of Muscle Damage or Inflammation After an Exercise-Induced Muscle Damage Protocol in Resistance-Trained Males. *J Strength Cond Res* **2023**, doi:10.1519/JSC.0000000000004408.
11. Wika, A.A.; Reason, K.W.; Green, J.M.; Killen, L.G.; McAllister, M.J.; Waldman, H.S. Astaxanthin Reduces Heart Rate and Carbohydrate Oxidation Rates During Exercise in Overweight Individuals. *Int J Exerc Sci* **2023**, *16*, 252-266.
12. D'Aloisio, R.; Di Antonio, L.; Toto, L.; Rispoli, M.; Di Iorio, A.; Delvecchio, G.; Mastropasqua, R. Choroidal Changes in Blood Flow in Patients with Intermediate AMD after Oral Dietary Supplement Based on Astaxanthin, Bromelain, Vitamin D3, Folic Acid, Lutein, and Antioxidants. *Medicina (Kaunas)* **2022**, *58*, doi:10.3390/medicina58081092.
13. Dede, G.; Saylan, A. The effect of astaxanthin on human sperm parameters after cryopreservation. *Can Urol Assoc J* **2022**, *16*, E552-E557, doi:10.5489/cuaj.7876.
14. Ghantabpour, T.; Nashtaei, M.S.; Nekoonam, S.; Rezaei, H.; Amidi, F. The Effect of Astaxanthin on Motility, Viability, Reactive Oxygen Species, Apoptosis, and Lipid Peroxidation of Human Spermatozoa During the Freezing-Thawing Process. *Biopreserv Biobank* **2022**, *20*, 367-373, doi:10.1089/bio.2021.0112.
15. Gharaei, R.; Alyasin, A.; Mahdavinezhad, F.; Samadian, E.; Ashrafnezhad, Z.; Amidi, F. Randomized controlled trial of astaxanthin impacts on antioxidant status and assisted reproductive technology outcomes in women with polycystic ovarian syndrome. *J Assist Reprod Genet* **2022**, *39*, 995-1008, doi:10.1007/s10815-022-02432-0.

16. Rad, N.R.; Movahedian, A.; Feizi, A.; Aminorroaya, A.; Aarabi, M.H. Antioxidant effects of astaxanthin and metformin combined therapy in type 2 diabetes mellitus patients: a randomized double-blind controlled clinical trial. *Res Pharm Sci* **2022**, *17*, 219-230, doi:10.4103/1735-5362.335179.
17. Stonehouse, W.; Benassi-Evans, B.; Bednarz, J.; Vincent, A.D.; Hall, S.; Hill, C.L. Krill oil improved osteoarthritic knee pain in adults with mild to moderate knee osteoarthritis: a 6-month multicenter, randomized, double-blind, placebo-controlled trial. *Am J Clin Nutr* **2022**, *116*, 672-685, doi:10.1093/ajcn/nqac125.
18. McAllister, M.J.; Mettler, J.A.; Patek, K.; Butawan, M.; Bloomer, R.J. Astaxanthin Supplementation Increases Glutathione Concentrations but Does Not Impact Fat Oxidation During Exercise in Active Young Men. *Int J Sport Nutr Exerc Metab* **2022**, *32*, 8-15, doi:10.1123/ijsnem.2021-0138.
19. Tian, L.; Wen, Y.; Li, S.; Zhang, P.; Wang, Y.; Wang, J.; Cao, K.; Du, L.; Wang, N.; Jie, Y. Benefits and Safety of Astaxanthin in the Treatment of Mild-To-Moderate Dry Eye Disease. *Front Nutr* **2021**, *8*, 796951, doi:10.3389/fnut.2021.796951.
20. Yamazaki, I.; Egawa, K.; Tanaka, K.; Rogi, T.; Nakai, M.; Miura, N. Effects of Sesame Lignans and Astaxanthin Supplementation on Daily Fatigue —A Randomized, Double–blind, Placebo–controlled, Parallel–group Comparison Study—. *Japanese Pharmacology & Therapeutics (Yakuri To Chiryo)* **2022**, *50*, 1111-1119.
21. Brown, D.R.; Warner, A.R.; Deb, S.K.; Gough, L.A.; Sparks, S.A.; McNaughton, L.R. The effect of astaxanthin supplementation on performance and fat oxidation during a 40 km cycling time trial. *J Sci Med Sport* **2021**, *24*, 92-97, doi:10.1016/j.jsams.2020.06.017.
22. Hashimoto, H.; Arai, K.; Takahashi, J.; Chikuda, M. The effect of aging on the antioxidative activity of astaxanthin in human aqueous humor. *J Clin Biochem Nutr* **2021**, *68*, 169-172, doi:10.3164/jcbn.20-87.
23. Hashimoto, H.A., K.; , Okamoto, Y.; Takahashi, J.; Chikuda, M.; Obara, Y. Effect of astaxanthin consumption on hydroperoxides in the aqueous. *Rinsho Ganka (Jpn J Clin Ophthalmol)* **2011**, *65*, 465-470, doi:10.11477/mf.1410103607.
24. Ishiwata, S.; Kato, T.; Kasai, T.; Sato, A.; Yatsu, S.; Matsumoto, H.; Shitara, J.; Murata, A.; Shimizu, M.; Suda, S.; et al. Changes in self-reported physical activity and health-related quality of life following 3-month astaxanthin supplementation in patients with heart failure: results from a pilot study. *Ann Palliat Med* **2021**, *10*, 1396-1403, doi:10.21037/apm-20-1378.
25. Kawamura, A.; Aoi, W.; Abe, R.; Kobayashi, Y.; Kuwahata, M.; Higashi, A. Astaxanthin-, beta-Carotene-, and Resveratrol-Rich Foods Support Resistance Training-Induced Adaptation. *Antioxidants (Basel)* **2021**, *10*, doi:10.3390/antiox10010113.
26. Kizawa, Y.; Sekikawa, T.; Kageyama, M.; Tomobe, H.; Kobashi, R.; Yamada, T. Effects of anthocyanin, astaxanthin, and lutein on eye functions: a randomized, double-blind, placebo-controlled study. *J Clin Biochem Nutr* **2021**, *69*, 77-90, doi:10.3164/jcbn.20-149.
27. Liu, S.Z.; Valencia, A.P.; VanDoren, M.P.; Shankland, E.G.; Roshanravan, B.; Conley, K.E.; Marcinek, D.J. Astaxanthin supplementation enhances metabolic adaptation with aerobic training in the elderly. *Physiol Rep* **2021**, *9*, e14887, doi:10.14814/phy2.14887.
28. Liu, S.Z.; Ali, A.S.; Campbell, M.D.; Kilroy, K.; Shankland, E.G.; Roshanravan, B.; Marcinek, D.J.; Conley, K.E. Building strength, endurance, and mobility using an astaxanthin formulation with functional training in elderly. *Journal of cachexia, sarcopenia and muscle* **2018**, *9*, 826-833, doi:10.1002/jcsm.12318.
29. McAllister, M.J.; Mettler, J.A.; Patek, K.; Butawan, M.; Bloomer, R.J. Astaxanthin Supplementation Increases Glutathione Concentrations but Does Not Impact Fat Oxidation During Exercise in Active Young Men. *Int J Sport Nutr Exerc Metab* **2021**, 1-8, doi:10.1123/ijsnem.2021-0138.
30. Nakanishi, R.; Kanazashi, M.; Tanaka, M.; Tanaka, M.; Fujino, H. Impacts of Astaxanthin Supplementation on Walking Capacity by Reducing Oxidative Stress in Nursing Home Residents. *Int J Environ Res Public Health* **2022**, *19*, doi:10.3390/ijerph192013492.
31. Shokri-Mashhadi, N.; Tahmasebi, M.; Mohammadi-Asl, J.; Zakerkish, M.; Mohammadshahi, M. The antioxidant and anti-inflammatory effects of astaxanthin supplementation on the expression of miR-146a and miR-126 in patients with type 2 diabetes mellitus: A randomised, double-blind, placebo-controlled clinical trial. *Int J Clin Pract* **2021**, *75*, e14022, doi:10.1111/ijcp.14022.

32. Urakaze, M.; Kobashi, C.; Satou, Y.; Shigeta, K.; Toshima, M.; Takagi, M.; Takahashi, J.; Nishida, H. The Beneficial Effects of Astaxanthin on Glucose Metabolism and Modified Low-Density Lipoprotein in Healthy Volunteers and Subjects with Prediabetes. *Nutrients* **2021**, *13*, doi:10.3390/nu13124381.
33. Birudaraju, D.; Cherukuri, L.; Kinninger, A.; Chaganti, B.T.; Shaikh, K.; Hamal, S.; Flores, F.; Roy, S.K.; Budoff, M.J. A combined effect of Cavacurcumin, Eicosapentaenoic acid (Omega-3s), Astaxanthin and Gamma - linoleic acid (Omega-6) (CEAG) in healthy volunteers- a randomized, double-blind, placebo-controlled study. *Clin Nutr ESPEN* **2020**, *35*, 174-179, doi:10.1016/j.clnesp.2019.09.011.
34. Hayashi, M.; Kawamura, M.; Kawashima, Y.; Uemura, T.; Maoka, T. Effect of astaxanthin-rich extract derived from *Paracoccus carotinifaciens* on the status of stress and sleep in adults. *J Clin Biochem Nutr* **2020**, *66*, 92-102, doi:10.3164/jcbn.19-113.
35. Kato, T.; Kasai, T.; Sato, A.; Ishiwata, S.; Yatsu, S.; Matsumoto, H.; Shitara, J.; Murata, A.; Shimizu, M.; Suda, S.; et al. Effects of 3-Month Astaxanthin Supplementation on Cardiac Function in Heart Failure Patients with Left Ventricular Systolic Dysfunction-A Pilot Study. *Nutrients* **2020**, *12*, 1896, doi:10.3390/nu12061896.
36. Kumalic, S.I.; Klun, I.V.; Bokal, E.V.; Pinter, B. Effect of the oral intake of astaxanthin on semen parameters in patients with oligo-astheno-teratozoospermia: a randomized double-blind placebo-controlled trial. *Radiol Oncol* **2020**, *55*, 97-105, doi:10.2478/raon-2020-0062.
37. Sudo, M.; Yamada, K.; Yazawa, K. The effects of supplement intake on female floorball athletes. *Kokushikan society of sport science [in Japanese]* **2020**, *20*, 39-46.
38. Chan, K.-c.; Chen, S.-c.; Chen, P.-c. Astaxanthin attenuated thrombotic risk factors in type 2 diabetic patients. *Journal of Functional Foods* **2019**, *53*, 22-27, doi:10.1016/j.jff.2018.12.012.
39. Fleischmann, C.; Horowitz, M.; Yanovich, R.; Raz, H.; Heled, Y. Asthaxanthin Improves Aerobic Exercise Recovery Without Affecting Heat Tolerance in Humans. *Front Sports Act Living* **2019**, *1*, 17, doi:10.3389/fspor.2019.00017.
40. Hashimoto, H.; Arai, K.; Takahashi, J.; Chikuda, M. Effects of astaxanthin on VEGF level and antioxidation in human aqueous humor: difference by sex. *J Clin Biochem Nutr* **2019**, *65*, 47-51, doi:10.3164/jcbn.18-110.
41. Landi, F.; Martone, A.M.; Salini, S.; Zazzara, B.; Calvani, R.; Marzetti, E.; Nesci, A.; Di Giorgio, A.; Giupponi, B.; Santoro, L.; et al. Effects of a New Combination of Medical Food on Endothelial Function and Lipid Profile in Dyslipidemic Subjects: A Pilot Randomized Trial. *Biomed Res Int* **2019**, *2019*, 1970878, doi:10.1155/2019/1970878.
42. Sudo, M.; Yamada, K.; Yazawa, K. Effects of consuming a supplement with antioxidant action on physical fatigue and the state of the skin in middle-aged women. *Kokushikan society of sport science [in Japanese]* **2019**, *19*, 21-26.
43. Sudo, A.; Yamada, K.; Yazawa, K. Effects of Consuming A Supplement with Antioxidant Action on Physical Fatigue and Sports Performance. *Japanese Journal of Complementary and Alternative Medicine* **2019**, *16*, 21-26, doi:10.1625/jcam.16.21.
44. Takami, M.; Aoi, W.; Terajima, H.; Tanimura, Y.; Wada, S.; Higashi, A. Effect of dietary antioxidant-rich foods combined with aerobic training on energy metabolism in healthy young men. *J Clin Biochem Nutr* **2019**, *64*, 79-85, doi:10.3164/jcbn.18-40.
45. Basioura, A.; Tsakmakidis, I.A.; Martinez, E.A.; Roca, J.; Li, J.; Molina, M.F.; Theodoridis, A.; Boscos, C.M.; Parrilla, I. Effect of astaxanthin in extenders on sperm quality and functional variables of frozen-thawed boar semen. *Anim Reprod Sci* **2020**, *218*, 106478, doi:10.1016/j.anireprosci.2020.106478.
46. Hayashi, M.; Ishibashi, T.; Maoka, T. Effect of astaxanthin-rich extract derived from *Paracoccus carotinifaciens* on cognitive function in middle-aged and older individuals. *J Clin Biochem Nutr* **2018**, *62*, 195-205, doi:10.3164/jcbn.17-100.
47. Hongo, N.; Tominaga, K.; Okuda, J.; Suzuki, M.; Miura, N. Effects of Astaxanthin on Cognitive Function, Physical Function and Mood Condition in Elderly People-A Randomized, Double-blind, Placebo-controlled, Parallel-group, Comparative Study-. *Japanese Pharmacology & Therapeutics （Yakuri To Chiryo) [in Japanse]* **2018**, *46*, 2043-2062.

48. Imai, A.; Oda, Y.; Ito, N.; Seki, S.; Nakagawa, K.; Miyazawa, T.; Ueda, F. Effects of Dietary Supplementation of Astaxanthin and Sesamin on Daily Fatigue: A Randomized, Double-Blind, Placebo-Controlled, Two-Way Crossover Study. *Nutrients* **2018**, *10*, doi:10.3390/nu10030281.
49. Ito, N.; Seki, S.; Ueda, F. The Protective Role of Astaxanthin for UV-Induced Skin Deterioration in Healthy People-A Randomized, Double-Blind, Placebo-Controlled Trial. *Nutrients* **2018**, *10*, doi:10.3390/nu10070817.
50. Ito, N.; Saito, H.; Seki, S.; Ueda, F.; Asada, T. Effects of Composite Supplement Containing Astaxanthin and Sesamin on Cognitive Functions in People with Mild Cognitive Impairment: A Randomized, Double-Blind, Placebo-Controlled Trial. *J Alzheimers Dis* **2018**, *62*, 1767-1775, doi:10.3233/JAD-170969.
51. Madhavi, D.; Kagan, D.; Seshadri, S. A Study on the Bioavailability of a Proprietary, Sustained-release Formulation of Astaxanthin. *Integr Med (Encinitas)* **2018**, *17*, 38-42.
52. Mashhadi, N.S.; Zakerkish, M.; Mohammadiasl, J.; Zarei, M.; Mohammadshahi, M.; Haghighizadeh, M.H. Astaxanthin improves glucose metabolism and reduces blood pressure in patients with type 2 diabetes mellitus. *Asia Pac J Clin Nutr* **2018**, *27*, 341-346, doi:10.6133/apjcn.052017.11.
53. Petyaev, I.M.; Klochkov, V.A.; Chalyk, N.E.; Pristensky, D.V.; Chernyshova, M.P.; Kyle, N.H.; Bashmakov, Y.K. Markers of Hypoxia and Oxidative Stress in Aging Volunteers Ingesting Lycosomal Formulation of Dark Chocolate Containing Astaxanthin. *J Nutr Health Aging* **2018**, *22*, 1092-1098, doi:10.1007/s12603-018-1063-z.
54. Sarkkinen, E.S.; Savolainen, M.J.; Taurio, J.; Marvola, T.; Bruheim, I. Prospective, randomized, double-blinded, placebo-controlled study on safety and tolerability of the krill powder product in overweight subjects with moderately elevated blood pressure. *Lipids Health Dis* **2018**, *17*, 287, doi:10.1186/s12944-018-0935-x.
55. Canas, J.A.; Lochrie, A.; McGowan, A.G.; Hossain, J.; Schettino, C.; Balagopal, P.B. Effects of Mixed Carotenoids on Adipokines and Abdominal Adiposity in Children: A Pilot Study. *J Clin Endocrinol Metab* **2017**, *102*, 1983-1990, doi:10.1210/jc.2017-00185.
56. Carrascosa, J.M.; Floriach, N.; Sala, E.; Aguilera, J. Increase in minimal erythemal dose following oral administration of an antioxidant complex based on a mix of carotenoids: Double-blind, placebo-controlled trial. *Photodermatol Photoimmunol Photomed* **2017**, *33*, 284-286, doi:10.1111/phpp.12315.
57. Chalyk, N.E.; Klochkov, V.A.; Bandaletova, T.Y.; Kyle, N.H.; Petyaev, I.M. Continuous astaxanthin intake reduces oxidative stress and reverses age-related morphological changes of residual skin surface components in middle-aged volunteers. *Nutr Res* **2017**, *48*, 40-48, doi:10.1016/j.nutres.2017.10.006.
58. Chen, J.T.; Kotani, K. Effects of Astaxanthin on Liver and Leukocyte Parameters in Healthy Climacteric Women: Preliminary Data. *J Med Food* **2017**, *20*, 724-725, doi:10.1089/jmf.2016.3819.
59. Hongo, N.; Fujishita, M.; Takahashi, Y.; Adachi, Y.; Takahashi, J.; Tominaga, K.; Miura, N. Daily fatigue-reducing effect of astaxanthin - A randomized, placebo-controlled, double-blind, parallel-group study. *Japanese Pharmacology & Therapeutics (Yakuri To Chiryo)* **2017**, *45*, 61-72.
60. Ledda, A.; Belcaro, G.; Dugall, M.; Luzzi, R.; Hosoi, M.; Feragalli, B.; Cotellese, R.; Cosentino, V.; Cosentino, M.; Eggenhoffner, R.; et al. A natural pharma standard supplement formulation to control treatment-related toxicity and oxidative stress in genitourinary cancer: a preliminary study. *Eur Rev Med Pharmacol Sci* **2017**, *21*, 4196-4202.
61. Kaneko, M.; Kishimoto, Y.; Suzuki, R.; Kawai, Y.; Tateya, I.; Hirano, S. Protective Effect of Astaxanthin on Vocal Fold Injury and Inflammation Due to Vocal Loading: A Clinical Trial. *J Voice* **2017**, *31*, 352-358, doi:10.1016/j.jvoice.2016.06.017.
62. Marazzi, G.; Campolongo, G.; Pelliccia, F.; Quattrino, S.; Vitale, C.; Cacciotti, L.; Massaro, R.; Volterrani, M.; Rosano, G. Comparison of Low-Dose Statin Versus Low-Dose Statin + Armolipid Plus in High-Intensity Statin-Intolerant Patients With a Previous Coronary Event and Percutaneous Coronary Intervention (ADHERENCE Trial). *Am J Cardiol* **2017**, *120*, 893-897, doi:10.1016/j.amjcard.2017.06.015.
63. Saito, H.; Cherasse, Y.; Suzuki, R.; Mitarai, M.; Ueda, F.; Urade, Y. Zinc-rich oysters as well as zinc-yeast- and astaxanthin-enriched food improved sleep efficiency and sleep onset in a randomized controlled trial of healthy individuals. *Mol Nutr Food Res* **2017**, *61*, doi:10.1002/mnfr.201600882.
64. Tominaga, K.; Hongo, N.; Fujishita, M.; Takahashi, Y.; Adachi, Y. Protective effects of astaxanthin on skin deterioration. *J Clin Biochem Nutr* **2017**, *61*, 33-39, doi:10.3164/jcbn.17-35.

65. Coombes, J.S.; Sharman, J.E.; Fassett, R.G. Astaxanthin has no effect on arterial stiffness, oxidative stress, or inflammation in renal transplant recipients: a randomized controlled trial (the XANTHIN trial). *Am J Clin Nutr* **2016**, *103*, 283-289, doi:10.3945/ajcn.115.115477.
66. Fassett, R.G.; Healy, H.; Driver, R.; Robertson, I.K.; Geraghty, D.P.; Sharman, J.E.; Coombes, J.S. Astaxanthin vs placebo on arterial stiffness, oxidative stress and inflammation in renal transplant patients (Xanthin): a randomised controlled trial. *BMC Nephrol* **2008**, *9*, 17, doi:10.1186/1471-2369-9-17.
67. Hashimoto, H.; Arai, K.; Hayashi, S.; Okamoto, H.; Takahashi, J.; Chikuda, M. The effect of astaxanthin on vascular endothelial growth factor (VEGF) levels and peroxidation reactions in the aqueous humor. *J Clin Biochem Nutr* **2016**, *59*, 10-15, doi:10.3164/jcbn.15-137.
68. Huang, J.Y.; Yeh, P.T.; Hou, Y.C. A randomized, double-blind, placebo-controlled study of oral antioxidant supplement therapy in patients with dry eye syndrome. *Clin Ophthalmol* **2016**, *10*, 813-820, doi:10.2147/OPTH.S106455.
69. Talbott, S.H., D.; Capelli, B.; Ding, L.; Li, Y.; Artaria, C. Effect of Astaxanthin Supplementation on Cardiorespiratory Function in Runners. *EC Nutrition* **2016**, *11*, 253-259, doi:http://dx.doi.org/10.1249/01.mss.0000519563.97355.e1.
70. Tsukahara, H.; Matsuyama, A.; Abe, T.; Kyo, H.; Ohta, T.; Suzuki, N. Effects of Intake of Astaxanthin Contained Drink on Skin Condition. *Japanese Journal of Complementary and Alternative Medicine* **2016**, *13*, 57-62, doi:10.1625/jcam.13.57.
71. Andrisani, A.; Dona, G.; Tibaldi, E.; Brunati, A.M.; Sabbadin, C.; Armanini, D.; Alvisi, G.; Gizzo, S.; Ambrosini, G.; Ragazzi, E.; et al. Astaxanthin Improves Human Sperm Capacitation by Inducing Lyn Displacement and Activation. *Mar Drugs* **2015**, *13*, 5533-5551, doi:10.3390/md13095533.
72. Dona, G.; Kozuh, I.; Brunati, A.M.; Andrisani, A.; Ambrosini, G.; Bonanni, G.; Ragazzi, E.; Armanini, D.; Clari, G.; Bordin, L. Effect of astaxanthin on human sperm capacitation. *Mar Drugs* **2013**, *11*, 1909-1919, doi:10.3390/md11061909.
73. Baralic, I.; Andjelkovic, M.; Djordjevic, B.; Dikic, N.; Radivojevic, N.; Suzin-Zivkovic, V.; Radojevic-Skodric, S.; Pejic, S. Effect of Astaxanthin Supplementation on Salivary IgA, Oxidative Stress, and Inflammation in Young Soccer Players. *Evid Based Complement Alternat Med* **2015**, *2015*, 783761, doi:10.1155/2015/783761.
74. Maki, K.C.; Geohas, J.G.; Dicklin, M.R.; Huebner, M.; Udani, J.K. Safety and lipid-altering efficacy of a new omega-3 fatty acid and antioxidant-containing medical food in men and women with elevated triacylglycerols. *Prostaglandins Leukot Essent Fatty Acids* **2015**, *99*, 41-46, doi:10.1016/j.plefa.2015.05.002.
75. Nakayama, T. [Effects of Astaxanthin on Menstrual cramps and Hypermenorrhea] *Obstetrical and Gynecological Practice [in Japanese]* **2015**, *64*, 961-966.
76. Phetcharat, L.; Wongsuphasawat, K.; Winther, K. The effectiveness of a standardized rose hip powder, containing seeds and shells of Rosa canina, on cell longevity, skin wrinkles, moisture, and elasticity. *Clin Interv Aging* **2015**, *10*, 1849-1856, doi:10.2147/CIA.S90092.
77. Takemoto, M.; Yamaga, M.; Furuichi, Y.; Yokote, K. Astaxanthin Improves Nonalcoholic Fatty Liver Disease in Werner Syndrome with Diabetes Mellitus. *J Am Geriatr Soc* **2015**, *63*, 1271-1273, doi:10.1111/jgs.13505.
78. Ni, Y.; Nagashimada, M.; Zhuge, F.; Zhan, L.; Nagata, N.; Tsutsui, A.; Nakanuma, Y.; Kaneko, S.; Ota, T. Astaxanthin prevents and reverses diet-induced insulin resistance and steatohepatitis in mice: A comparison with vitamin E. *Sci Rep* **2015**, *5*, 17192, doi:10.1038/srep17192.
79. Balcerczyk, A.; Gajewska, A.; Macierzynska-Piotrowska, E.; Pawelczyk, T.; Bartosz, G.; Szemraj, J. Enhanced antioxidant capacity and anti-ageing biomarkers after diet micronutrient supplementation. *Molecules* **2014**, *19*, 14794-14808, doi:10.3390/molecules190914794.
80. Kono, K.; Shimizu, Y.; Takahashi, S.; Matsuoka, S.; Yui, K. Effect of multiple dietary supplement containing lutein, astaxanthin, cyanidin-3-glucoside, and DHA on accommodative ability. *Immunology, Endocrine & Metabolic Agents in Medicinal Chemistry (Formerly Current Medicinal Chemistry-Immunology, Endocrine and Metabolic Agents)* **2014**, *14*, 114-125.
81. Yoon, H.S.; Cho, H.H.; Cho, S.; Lee, S.R.; Shin, M.H.; Chung, J.H. Supplementating with dietary astaxanthin combined with collagen hydrolysate improves facial elasticity and decreases matrix metalloproteinase-1 and -12 expression: a comparative study with placebo. *J Med Food* **2014**, *17*, 810-816, doi:10.1089/jmf.2013.3060.

82. Zanotta, D.; Puricelli, S.; Bonoldi, G. Cognitive effects of a dietary supplement made from extract of Bacopa monnieri, astaxanthin, phosphatidylserine, and vitamin E in subjects with mild cognitive impairment: a noncomparative, exploratory clinical study. *Neuropsychiatr Dis Treat* **2014**, *10*, 225-230, doi:10.2147/NDT.S51092.
83. Baralic, I.; Djordjevic, B.; Dikic, N.; Kotur-Stevuljevic, J.; Spasic, S.; Jelic-Ivanovic, Z.; Radivojevic, N.; Andjelkovic, M.; Pejic, S. Effect of astaxanthin supplementation on paraoxonase 1 activities and oxidative stress status in young soccer players. *Phytother Res* **2013**, *27*, 1536-1542, doi:10.1002/ptr.4898.
84. Djordjevic, B.; Baralic, I.; Kotur-Stevuljevic, J.; Stefanovic, A.; Ivanisevic, J.; Radivojevic, N.; Andjelkovic, M.; Dikic, N. Effect of astaxanthin supplementation on muscle damage and oxidative stress markers in elite young soccer players. *J Sports Med Phys Fitness* **2012**, *52*, 382-392.
85. Hashimoto, H.; Arai, K.; Hayashi, S.; Okamoto, H.; Takahashi, J.; Chikuda, M.; Obara, Y. Effects of astaxanthin on antioxidation in human aqueous humor. *J Clin Biochem Nutr* **2013**, *53*, 1-7, doi:10.3164/jcbn.13-6.
86. Klinkenberg, L.J.; Res, P.T.; Haenen, G.R.; Bast, A.; van Loon, L.J.; van Dieijen-Visser, M.P.; Meex, S.J. Effect of antioxidant supplementation on exercise-induced cardiac troponin release in cyclists: a randomized trial. *PLoS One* **2013**, *8*, e79280, doi:10.1371/journal.pone.0079280.
87. Res, P.T.; Cermak, N.M.; Stinkens, R.; Tollakson, T.J.; Haenen, G.R.; Bast, A.; Van Loon, L.J. Astaxanthin supplementation does not augment fat use or improve endurance performance. *Med Sci Sports Exerc* **2013**, *45*, 1158-1165, doi:10.1249/MSS.0b013e31827fddc4.
88. Yagi, H.; Kawaguchi, M.; Nishio, K.; Sato, R.; Kobori, Y.; Arai, G.; Okada, H. Effect of Astaxanthin for Anticholinergic Agent-resistant Overactive Bladder. *Anti-Aging Medicine [in Japanese]* **2013**, *9*, 612-616.
89. Katagiri, M.; Satoh, A.; Tsuji, S.; Shirasawa, T. Effects of astaxanthin-rich Haematococcus pluvialis extract on cognitive function: a randomised, double-blind, placebo-controlled study. *J Clin Biochem Nutr* **2012**, *51*, 102-107, doi:10.3164/jcbn.D-11-00017.
90. Kiko, T.; Nakagawa, K.; Satoh, A.; Tsuduki, T.; Furukawa, K.; Arai, H.; Miyazawa, T. Amyloid beta levels in human red blood cells. *PLoS One* **2012**, *7*, e49620, doi:10.1371/journal.pone.0049620.
91. Nakagawa, K.; Kiko, T.; Miyazawa, T.; Carpentero Burdeos, G.; Kimura, F.; Satoh, A.; Miyazawa, T. Antioxidant effect of astaxanthin on phospholipid peroxidation in human erythrocytes. *Br J Nutr* **2011**, *105*, 1563-1571, doi:10.1017/S0007114510005398.
92. Piermarocchi, S.; Saviano, S.; Parisi, V.; Tedeschi, M.; Panozzo, G.; Scarpa, G.; Boschi, G.; Lo Giudice, G.; Carmis Study, G. Carotenoids in Age-related Maculopathy Italian Study (CARMIS): two-year results of a randomized study. *Eur J Ophthalmol* **2012**, *22*, 216-225, doi:10.5301/ejo.5000069.
93. Macdermid, J.C.; Vincent, J.I.; Gan, B.S.; Grewal, R. A blinded placebo-controlled randomized trial on the use of astaxanthin as an adjunct to splinting in the treatment of carpal tunnel syndrome. *Hand (N Y)* **2012**, *7*, 1-9, doi:10.1007/s11552-011-9381-1.
94. Saito, M.; Yoshida, K.; Saito, W.; Fujiya, A.; Ohgami, K.; Kitaichi, N.; Tsukahara, H.; Ishida, S.; Ohno, S. Astaxanthin increases choroidal blood flow velocity. *Graefes Arch Clin Exp Ophthalmol* **2012**, *250*, 239-245, doi:10.1007/s00417-011-1843-1.
95. Suganuma, K.; Shiobara, M.; Yuki Sato, Y.; Nakanuma, C.; Maekawa, T.; Ohtsuki, M.; Yazawa, K.; Imokawa, G. Anti-aging and functional improvement effects for the skin by functional foods intakes: clinical effects on skin by oral ingestion of preparations containing Astaxanthin and Vitamins C and E. *Jichi Medical University Journal* **2012**, *35*, 25-33.
96. Tominaga, K.; Hongo, N.; Karato, M.; Yamashita, E. Cosmetic benefits of astaxanthin on humans subjects. *Acta Biochim Pol* **2012**, *59*, 43-47.
97. Choi, H.D.; Kim, J.H.; Chang, M.J.; Kyu-Youn, Y.; Shin, W.G. Effects of astaxanthin on oxidative stress in overweight and obese adults. *Phytother Res* **2011**, *25*, 1813-1818, doi:10.1002/ptr.3494.
98. Choi, H.D.; Youn, Y.K.; Shin, W.G. Positive effects of astaxanthin on lipid profiles and oxidative stress in overweight subjects. *Plant Foods Hum Nutr* **2011**, *66*, 363-369, doi:10.1007/s11130-011-0258-9.
99. Earnest, C.P.; Lupo, M.; White, K.M.; Church, T.S. Effect of astaxanthin on cycling time trial performance. *Int J Sports Med* **2011**, *32*, 882-888, doi:10.1055/s-0031-1280779.

100. Kim, J.H.; Chang, M.J.; Choi, H.D.; Youn, Y.K.; Kim, J.T.; Oh, J.M.; Shin, W.G. Protective effects of Haematococcus astaxanthin on oxidative stress in healthy smokers. *J Med Food* **2011**, *14*, 1469-1475, doi:10.1089/jmf.2011.1626.
101. Miyazawa, T.; Nakagawa, K.; Kimura, F.; Satoh, A.; Miyazawa, T. Erythrocytes carotenoids after astaxanthin supplementation in middle-aged and senior Japanese subjects. *J Oleo Sci* **2011**, *60*, 495-499, doi:10.5650/jos.60.495.
102. Miyazawa, T.; Nakagawa, K.; Kimura, F.; Satoh, A.; Miyazawa, T. Plasma carotenoid concentrations before and after supplementation with astaxanthin in middle-aged and senior subjects. *Biosci Biotechnol Biochem* **2011**, *75*, 1856-1858, doi:10.1271/bbb.110368.
103. Peng, L., Zhao, P.; Li, B.; Zhang, J.; Huang, C. Antioxidant effects and impact on human health of astaxanthin. *Chinese Journal of Food Hygiene* **2011**, *23*, 313–316.
104. Satoh, A.; Mitarai, A.; Kanno, T.; Hori, Y.; Takeda, R. Effects of the lntake of Astaxanthin on the Reduction of Skin Darkling Induced by UV lrradiation in adult women. *Pharmacometrics (OyoYakuri) (in Japanse)* **2011**, *80*, 7-11.
105. Nagaki, Y.; Tsukahara, H.; Yoshimoto, T.; Masuda, K. Effect of Astaxanthin on Accommodation and Asthenopia. Japanese Review of Clinical Ophthalmology, . *Folia Opththalmologica Japonica. [in Japanse]* **2010**, *3*, 461–468.
106. Park, J.S.; Chyun, J.H.; Kim, Y.K.; Line, L.L.; Chew, B.P. Astaxanthin decreased oxidative stress and inflammation and enhanced immune response in humans. *Nutr Metab (Lond)* **2010**, *7*, 18, doi:10.1186/1743-7075-7-18.
107. Yamada, T.; Ryo, K.; Tai, Y.; Tamaki, Y.; Inoue, H.; Mishima, K.; Tsubota, K.; Saito, I. Evaluation of therapeutic effects of astaxanthin on impairments in salivary secretion. *J Clin Biochem Nutr* **2010**, *47*, 130-137, doi:10.3164/jcbn.10-31.
108. Yoshida, H.; Yanai, H.; Ito, K.; Tomono, Y.; Koikeda, T.; Tsukahara, H.; Tada, N. Administration of natural astaxanthin increases serum HDL-cholesterol and adiponectin in subjects with mild hyperlipidemia. *Atherosclerosis* **2010**, *209*, 520-523, doi:10.1016/j.atherosclerosis.2009.10.012.
109. Iwabayashi, M.; Fujioka, N.; Nomoto, K.; Miyazaki, R.; Takahashi, H.; Hibino, S.; Takahashi, Y.; Nishikawa, K.; Nishida, M.; Yonei, Y. Efficacy and safety of eight-week treatment with astaxanthin in individuals screened for increased oxidative stress burden. *Anti-Aging Medicine* **2009**, *6*, 15-21, doi:10.3793/jaam.6.15.
110. Kajita, M.; Tsukahara, H.; Mio, K. The Effects of a Dietary Supplement Containing Astaxanthin on the Accommodation Function of the Eye in Middle-aged and Older People. *Medical Consultation & New Remedies [in Jpananese]* **2009**, *46*, 325-329.
111. Okada, Y.; Ishikura, M.; Maoka, T. Bioavailability of astaxanthin in *Haematococcus* algal extract: the effects of timing of diet and smoking habits. *Biosci Biotechnol Biochem* **2009**, *73*, 1928-1932, doi:10.1271/bbb.90078.
112. Satoh, A.; Tsuji, S.; Okada, Y.; Murakami, N.; Urami, M.; Nakagawa, K.; Ishikura, M.; Katagiri, M.; Koga, Y.; Shirasawa, T. Preliminary Clinical Evaluation of Toxicity and Efficacy of A New Astaxanthin-rich Haematococcus pluvialis Extract. *J Clin Biochem Nutr* **2009**, *44*, 280-284, doi:10.3164/jcbn.08-238.
113. Satoh, A.; Kawamura, T.; horibe, N.; Ito, T.; Yagi, H.; Hashizume, H.; Takigawa, M. Effect of the intake of astaxanthin-containing *Haematococcus pluvialis* extract on the severity, immunofunction and physiological function in patients with atopic dermatitis. *Journal of environmental dermatology and cutaneous allergology (in Japanese)* **2009**, *3*, 429-438.
114. Seya, Y.; Takahashi, J.; Imanaka, K. Relationships Between Visual Fatigue and Reaction Times −Effects of a Repetition of a Visual Task and Long-term Intake of a Supplementation Food Including Astaxanthin on Reaction Time ―. *Japanese Journal of Physiological Anthropology [in Japanese]* **2009**, *14*, 59-66, doi:10.20718/jjpa.14.2_59.
115. Kupcinskas, L.; Lafolie, P.; Lignell, A.; Kiudelis, G.; Jonaitis, L.; Adamonis, K.; Andersen, L.P.; Wadstrom, T. Efficacy of the natural antioxidant astaxanthin in the treatment of functional dyspepsia in patients with or without Helicobacter pylori infection: A prospective, randomized, double blind, and placebo-controlled study. *Phytomedicine* **2008**, *15*, 391-399, doi:10.1016/j.phymed.2008.04.004.

116. Malmstena, C.L.L., A. Dietary Supplementation with Astaxanthin-Rich Algal Meal Improves Strength Endurance – A Double Blind Placebo Controlled Study on Male Students –. *Carotenoid Science* **2008**, *13*, 20-22.
117. Miyawaki, H.; Takahashi, J.; Tsukahara, H.; Takehara, I. Effects of astaxanthin on human blood rheology. *J Clin Biochem Nutr* **2008**, *43*, 69-74, doi:10.3164/jcbn.2008048.
118. Parisi, V.; Tedeschi, M.; Gallinaro, G.; Varano, M.; Saviano, S.; Piermarocchi, S.; Group, C.S. Carotenoids and antioxidants in age-related maculopathy italian study: multifocal electroretinogram modifications after 1 year. *Ophthalmology* **2008**, *115*, 324-333 e322, doi:10.1016/j.ophtha.2007.05.029.
119. Tsukahara, H.; Koikeda, T.; Arai, T.; Hayashi, H.; Ohno, S.; Suzuki, N. Supplementation Effect of Astaxanthin on Blood Flow and Shoulder Stiffness -A Preliminary Pilot Study. *Japanese Journal of Complementary and Alternative Medicine* **2008**, *5*, 49-56, doi:10.1625/jcam.5.49.
120. Uchiyama, A.; Okada, Y. Clinical efficacy of astaxanthin-containing Haematococcus pluvialis extract for the volunteers at risk of metabolic syndrome. *Journal of Clinical Biochemistry and Nutrition* **2008**, *43*, 38-43.
121. Andersen, L.P.; Holck, S.; Kupcinskas, L.; Kiudelis, G.; Jonaitis, L.; Janciauskas, D.; Permin, H.; Wadstrom, T. Gastric inflammatory markers and interleukins in patients with functional dyspepsia treated with astaxanthin. *FEMS Immunol Med Microbiol* **2007**, *50*, 244-248, doi:10.1111/j.1574-695X.2007.00257.x.
122. Fukamauchi, M. Food functionality of astaxanthin-10: Synergistic effects of astaxanthin intake and aerobic exercise. *FOOD STYLE 21 (JP)* **2007**, *11*, 22-24.
123. Karppi, J.; Rissanen, T.H.; Nyyssonen, K.; Kaikkonen, J.; Olsson, A.G.; Voutilainen, S.; Salonen, J.T. Effects of astaxanthin supplementation on lipid peroxidation. *Int J Vitam Nutr Res* **2007**, *77*, 3-11, doi:10.1024/0300-9831.77.1.3.
124. Iwasaki, T.; Tawara, A. Effects of Astaxanthin on Eyestrain Induced by Accommodative Dysfunction. *Atarashii Ganka (Journal of the Eye) [in Japanese]* **2006**, *23*, 829-834.
125. Nagaki, Y.; Mihara, M.; Tsukuhara, H.; Ohno, S. The supplementation effect of astaxanthin on accommodation and asthenopia. *Rinsho Iyaku (Journal of Clinical Therapeutics & Medicines) [in Japanese]* **2006**, *22*.
126. Yamashita, E. The Effects of a Dietary Supplement Containing Astaxanthin on Skin Condition. *Carotenoid Science* **2006**, *10*, 91-95.
127. Bloomer, R.J.; Fry, A.; Schilling, B.; Chiu, L.; Hori, N.; Weiss, L. Astaxanthin supplementation does not attenuate muscle injury following eccentric exercise in resistance-trained men. *Int J Sport Nutr Exerc Metab* **2005**, *15*, 401-412, doi:10.1123/ijsnem.15.4.401.
128. Comhaire, F.H.; El Garem, Y.; Mahmoud, A.; Eertmans, F.; Schoonjans, F. Combined conventional/antioxidant "Astaxanthin" treatment for male infertility: a double blind, randomized trial. *Asian J Androl* **2005**, *7*, 257-262, doi:10.1111/j.1745-7262.2005.00047.x.
129. Nagaki, Y.; Mihara, M.; Takahashi, J.; Kitamura, A.; Horita, Y.; Sugiura, Y.; Tsukahara, H. The effect of astaxanthin on retinal capillary blood flow in normal volunteers. *J. Clin. Ther. Med* **2005**, *21*.
130. Nitta, T.; Ohgami, K.; Shiratori, K.; Chin, S.; Yoshida, K.; Tsukahara, H.; Ohno, S. Effects of Astaxanthin on Accommodation and Asthenopia-Dose Finding Study in Healthy Volunteers-. *Rinsho Iyaku (Journal of Clinical Therapeutics & Medicines) [in Japanese]* **2005**, *21*, 543-556.
131. Shiratori, K.; Ohgami, K.; Nitta, T.; Shinmei, Y.; Chin, S.; Yoshida, K.; Tsukahara, H.; Takehara, I.; Ohno, S. Effect of Astaxanthin on Accommodation and Asthenopia-Efficacy-Identification Study in Healthy Volunteers-. *Rinsho Iyaku (Journal of Clinical Therapeutics & Medicines) [in Japanese]* **2005**, *21*, 637-650.
132. Takahashi, N.; Kajita, M. Effects of astaxanthin on accommodative recovery. *Rinsho Iyaku (Journal of Clinical Therapeutics & Medicines) [in Japanese]* **2005**, *21*, 431-436.
133. Coral-Hinostroza, G.N.; Ytrestoyl, T.; Ruyter, B.; Bjerkeng, B. Plasma appearance of unesterified astaxanthin geometrical *E/Z* and optical *R/S* isomers in men given single doses of a mixture of optical 3 and 3'*R/S* isomers of astaxanthin fatty acyl diesters. *Comp Biochem Physiol C Toxicol Pharmacol* **2004**, *139*, 99-110, doi:10.1016/j.cca.2004.09.011.
134. Kim, Y., K.;Chyun, J,H. The Effects of Astaxanthin Supplements on Lipid Peroxidation and Antioxidant Status in Postmenopausal Women *Nutritional Sciences* **2004**, *7*, 41-46.

135. Nakamura, A.; Isobe, A.; Ootaka, Y.; Abematsu, Y.; Nakata, D.; Honma, K.; Sakurai, S.; Shimada, Y.; Horiguchi, M. Changes in visual function following peroral astaxanthin "". *RinGan "Rinsho Ganka" [article in Japanse]* **2004**, *58*, 1051-1054, doi:10.11477/mf.1410100622.
136. Tajima, T.; Nagata, A. Effects of astaxanthin ingestion on exercise-induced physiological changes. *Health and Behavior Sciences* **2004**, *3*, 5-10, doi:10.32269/hbs.3.1_5.
137. Mercke Odeberg, J.; Lignell, A.; Pettersson, A.; Hoglund, P. Oral bioavailability of the antioxidant astaxanthin in humans is enhanced by incorporation of lipid based formulations. *Eur J Pharm Sci* **2003**, *19*, 299-304, doi:10.1016/s0928-0987(03)00135-0.
138. Nagaki, Y.; Hayasaka, S.; Yamada, T.; Hayasaka, Y.; Sanada, M.; Uonomi, T. Effects of astaxanthin on accommodation, critical flicker fusion, and pattern visual evoked potential in visual display terminal workers. *Journal of traditional medicines [in Japanse]* **2002**, *19*, 170-173.
139. Sawaki, K.; Yoshigi, H.; Aoki, K.; Koikawa, N.; Azumane, A.; Kaneko, K.; Yamaguchi, M. Sports Performance Benefits from Taking Natural Astaxanthin Characterized by Visual Acuity and Muscle Fatigue Improvement in Humans. *Journal of Clinical Therapeutics & Medicines [in Japanese]* **2002**, *18*, 1085-1100.
140. Yamashita, E. Cosmetic benefit of dietary supplements containing astaxanthin and tocotrienol on human skin *FOOD Style 21 [Japanese]* **2002**, *6*, 112-117.
141. Osterlie, M.; Bjerkeng, B.; Liaaen-Jensen, S. Plasma appearance and distribution of astaxanthin *E*/*Z* and *R*/*S* isomers in plasma lipoproteins of men after single dose administration of astaxanthin. *J Nutr Biochem* **2000**, *11*, 482-490, doi:10.1016/s0955-2863(00)00104-2.
142. Nishida, Y.; Nawaz, A.; Hecht, K.; Tobe, K. Astaxanthin as a Novel Mitochondrial Regulator: A New Aspect of Carotenoids, beyond Antioxidants. *Nutrients* **2021**, *14*, doi:10.3390/nu14010107.
