# Supplementary material for: Astaxanthin: Past, Present, and Future
Source: Mar Drugs. 2023 Sep 28;21(10):514. doi: 10.3390/md21100514 (PMC10608541; doi:10.3390/md21100514)
Supplement: Supplementary file 1 [file marinedrugs-21-00514-s001.zip › marinedrugs-21-00514-s001/S4.pdf]

Supplementary Table S5. Astaxanthin. Update on the Efficacy of Topical Use in Human Clinical Trials.

Author/year/ reference	Study design	Subjects	Dose#	Duration		Outcome†	Description
Kosyreva TF. *et al.*, 2022 [1]	3 arms quasi-experimental prospective study	105 patients with partial adentia aged 30-65 yrs.;	0.026% in gel*	7 days	✓	Dental health	The main group (*n*=35) received astaxanthin (AX) gel within 7 days after the placement of the removable denture. The patients in the comparison group (*n*=35) did not receive AX gel after the placement of the removable denture. The control group (*n*=25) did not receive any prophylactic agents and did not have removable dentures.In patients with immediate and partial dentures, the preventive and anti-inflammatory effects of AX gel were confirmed: the use of the gel for 7 days reduced hygiene indicators, bacterial plasmalogens and endotoxin levels in the oral fluid from a short-term perspective. *No safety concerns were identified in this study.* [Article in Russian]
Trakanwittayarak S, Meephansan J. 2019 [2]	Placebo-controlled repeated measured prospective study	13 volunteers with allergy associated with *p*-phenylenediamine	0.07% AX emulsion	7 days	✓	Allergic contact dermatitis (*p*-phenylenedia.)	On Day 2, pretreatment with AX emulsion resulted in a reaction in 5 of 12 patients ($p = 0.025$). On Day 7, pretreatment of skin sites with AX reduced the cutaneous allergic reaction to *p*-phenylenediamine in 2 of 12 patients ($p = 0.046$) as compared with skin treated with AX-free emulsion. *There were no serious adverse effects with patch test.*
Tominaga K. *et al.* 2012 [3] (Study 1)	Open-labeled, prospective study	30 healthy females (22-55 yrs.)	78.9 μM AX solution (1mL/day) and 6 mg/day p.o.	8 weeks	✓	Skin health	Combined dosing study with oral intake (p.o.) and topical application. Age spot (left cheek): significantly improved after treatment. Wrinkle depth (left eye corner): significantly improved in several parameters after treatment. Elasticity (left eye corner): significantly improved after treatment. Texture (left cheek): significantly improved after treatment. Moisture content (left cheek): Not significant (N.S.) *No safety concerns were identified in this study.*

Seki T. *et al.*, 2001 [4] Study 2 (Primary skin irritation test.)	Open-labeled, prospective study	45 healthy subjects	5% oil, 0, 0.7mg/g in cream	48 hours	✓ Skin health<br>✓ (Safety)	A few people in each group showed weak primary irritation, but this was not significantly different from the base product - judged to be safe products.
Seki T. *et al.*, 2001 [4] Study 2 (repeated application)	Open-labeled, prospective study	11 healthy subjects (Mean 35 yrs.)	0.7mg/g in cream (20mg, b.i.d)	3 weeks	✓ Skin health<br>✓ (Safety)	Water content (left eye corner): significantly improved. Inspection by dermatologist : partially effective for chloasma and senile pigment spot. Subjective symptoms (visual analogue scale: VAS): improvement trend in dryness, flushing, poor make-up application, itching and eczema.<br><br>*No serious advierse effects were identified in this study.*
Seki T. *et al.*, 2001 [4] Study 2	Open-labeled, prospective study	3 healthy females (Mean 33 yrs.)	0.7mg/g in cream (20mg, b.i.d)	2 weeks	✓ Skin health	Inspection/Palpation by dermatologist: improvement trend in wrinkles<br>Wrinkle depth (left eye corner): improvement trend<br>Moisture and sebum content (left and right eye corner, left and right cheeks): trend towards improvement in terms of moisture content.<br>*No serious advierse effects were identified in this study.*
Yamashita E. 1995 [5]	Open-labeled, prospective study	7 male subjects (Skin type III)	ODT	24 hour	✓ Skin health	*AX were derived from krill and synthetic product. AXs were applies by Occlusive Dressing Technique (ODT) for 24 hours. The areas of ODT were then wiped with alcohol, irradiated with 2 MED UVB and observed for erythema and hyperpigmentation up to 1 week.*<br>Topical astaxanthin from krill suppressed of post-UVB erythema and hyperpigmentation.<br><br>*No serious advierse effects were identified in this study.*

Reference:

1. Kosyreva, T.F.; Samoylova, M.B.; Voeykova, O.V.; Belfer, M.L.; Zatevalov, A.M.; Voropayeva, E.A.; Zhilenkova, O.G. The effect of astaxanthin on the adaptation of the oral mucosa to a removable denture. *Stomatologiia (Mosk)* [in Russian] **2022**, *101*, 17-22, doi:10.17116/stomat202210101117.
2. Trakanwittayarak, S.; Meephansan, J. The effect of astaxanthin on allergic contact dermatitis in response to hair dye containing p-phenylenediamine. *Eur J Dermatol* **2019**, *29*, 647-648, doi:10.1684/ejd.2019.3654.
3. Tominaga, K.; Hongo, N.; Karato, M.; Yamashita, E. Cosmetic benefits of astaxanthin on humans subjects. *Acta Biochim Pol* **2012**, *59*, 43-47.
4. Seki, T.; Sueki, H.; Kono, H.; Suganuma, K.; Yamashita, E. Effects of astaxanthin from *Haematococcus pluvialis* on human skin-patch test; skin repeated application test; effect on wrinkle reduction. *Fragrance J* **2001**, *12*, 98-103.
5. Yamashita, E. Suppression of post-UVB hyperpigmentation. *Fragrance J* [in Japanese] **1995**, *14*, 180-185.
