# Supplementary material for: Astaxanthin: Past, Present, and Future
Source: Mar Drugs. 2023 Sep 28;21(10):514. doi: 10.3390/md21100514 (PMC10608541; doi:10.3390/md21100514)
Supplement: Supplementary file 1 [file marinedrugs-21-00514-s001.zip › marinedrugs-21-00514-s001/SI(Section214).pdf]

2.1.1 Astaxanthin; Optical Isomers (Supplement)

Stereo-chemistry of AX.

Since AX has asymmetric carbon atoms (chiral or stereo centers) in C3 and C3' positions, there are in principle three stereo isomers in AX, i.e. (3*R*,3'*R*)-, (3*R*,3'*S*)-, and (3*S*,3'*S*)-isomers (see Figure X1). The assignment of the absolute configurations of these stereo isomers is merely depending on the sequence rule of the stereo-chemistry of AX, although which is somewhat confusing. In this supplementary section, how to assign the absolute configurations of AX is explicitly explained.

The "right hand" and "left hand" nomenclature is used to distinguish the enantiomers of a chiral compound. The stereocenters (chiral centers) are labeled as *R* or *S*. As illustrated in Figure X2, a curved arrow is drawn from the highest priority substituent **1** to the second lowest priority substituent **3**. When the arrow points in a counterclockwise direction (left-hand side of the upper panel in Figure X2), the configuration at the stereocenter is called *S* ("Sinister" in Latin, which means "left"), while when the arrow points clockwise (right-hand side of the upper panel in Figure X2), the stereocenter is called *R* ("Rectus" in Latin, which means "right"). Before applying the *R* and *S* nomenclature to a stereocenter, the substituents should be prioritized according to the sequence rules, which is summarized below.

Rule 1: First, examine at the atoms directly attached to the stereocenter of the compound. A substituent with higher atomic number takes precedence over a substituent with a lower atomic number. Hydrogen is the lowest possible priority substituent because it has the lowest atomic number. When dealing with isotopes, the atom with the higher atomic mass acquires higher priority. When visualizing the molecule, the lowest priority substituent should always point away from the viewer (a dashed line in Figure X2 indicates this). Then, draw an arrow from the highest priority atom to the 2nd highest priority atom to the 3rd highest priority atom.

Rule 2: If there are two substituents with equal rank, proceed along the two substituent chains until there is a point of difference. First, determine which of the chains has the first connection to an atom with the highest priority (the highest atomic number). That chain has the higher priority. If the chains are similar, proceed down the chain, until a point of difference.

Rule 3: If a chain is connected to the same kind of atom twice or three times, check to see if the atom it is connected to has a greater atomic number than any of the atoms that the competing chain is connected to. If none of the atoms connected to the competing chain(s) at the same point has a greater atomic number, the chain bonded to the same atom multiple times has the greater priority. If, however, one of the atoms connected to the competing chain has a higher atomic number, that chain has the higher priority. Remember that being double or triple bonded to an atom means that

the atom is connected to the same atom twice. In such a case, follow the same method as above. It is also to be noted that priority is determined by the first point of difference along the two similar substituent chains. After the first point of difference, the rest of the chain is irrelevant. When looking for the first point of difference on similar substituent chains, one may encounter branching. If there is branching, choose the branch that is higher in priority. If the two substituents have similar branches, rank the elements within the branches until a point of difference.

As illustrated in Figure X3, C3 (or C3') stereocenter of AX can be classified as *S*- or *R*- configuration according to the above-mentioned sequence rules. By referring to **Rule 1**, hydroxy oxygen attached to the C3 atom has the first priority. Then, the carbon atom at the position 4 (C4 carbon) has the second priority due to **Rule 2** because the carbonyl oxygen is attached to the C4 carbon. Hence, the carbon and position 2 (C2 carbon) has the third priority since the hydrogen atom that is attached to the C2 carbon has the lowest priority. Therefore, the enantiomer show in the left panel of Figure X3 can be assigned to the *S*-configuration as illustrated with the red curved arrow that shows counterclockwise rotation. With regard to the enantiomer that is shown in the middle panel of Figure X3, the chiral compound must be flipped so as the hydrogen atom that has the lowest priority goes away from the viewer; the right panel in Figure X3 corresponds to this configuration. Then, as illustrated with the curved green-arrow that shows clockwise rotation, the enantiomer is assigned to the *R*-configuration.

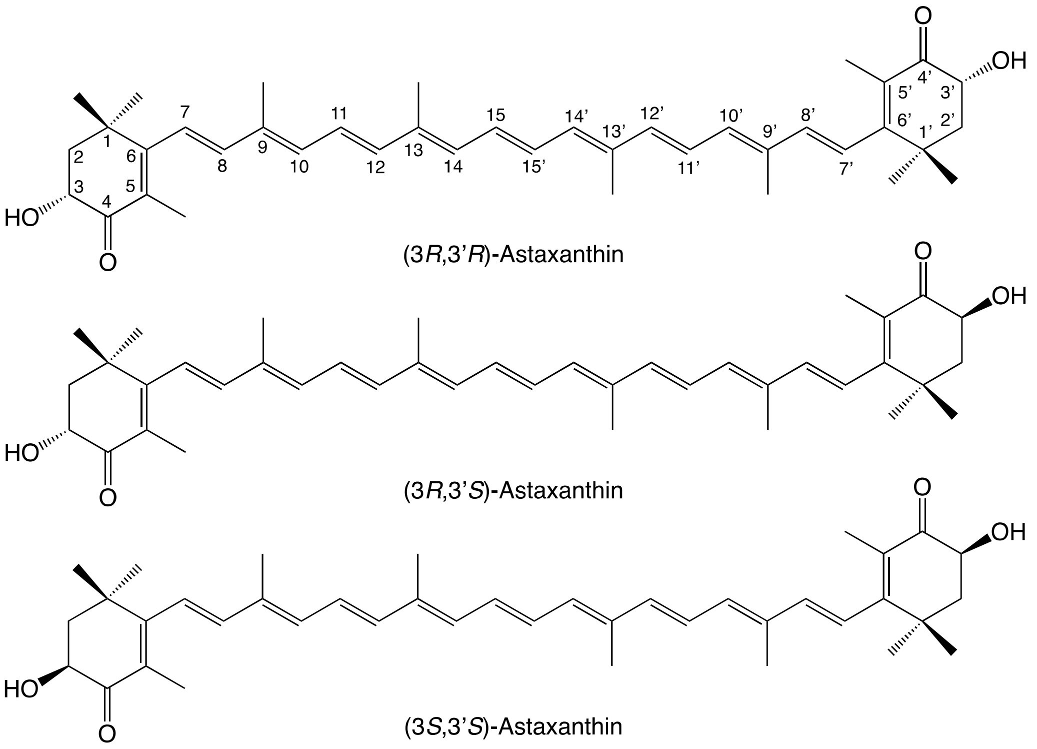

Figure X1. Chemical structures of the stereo-isomers of all-*trans* astaxanthin. The numbers of each carbon atom are shown in the (3*R*,3'*R*)-isomer for reference.

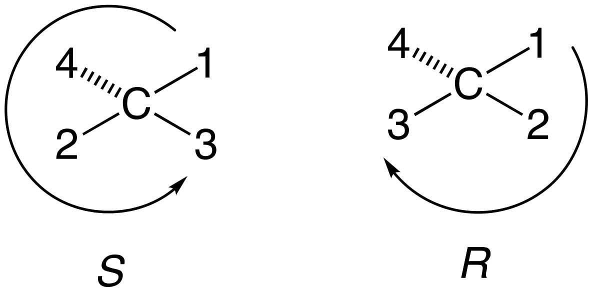

Figure X2. Schematic description that shows the stereocenters (chiral centers) with R or S configuration.

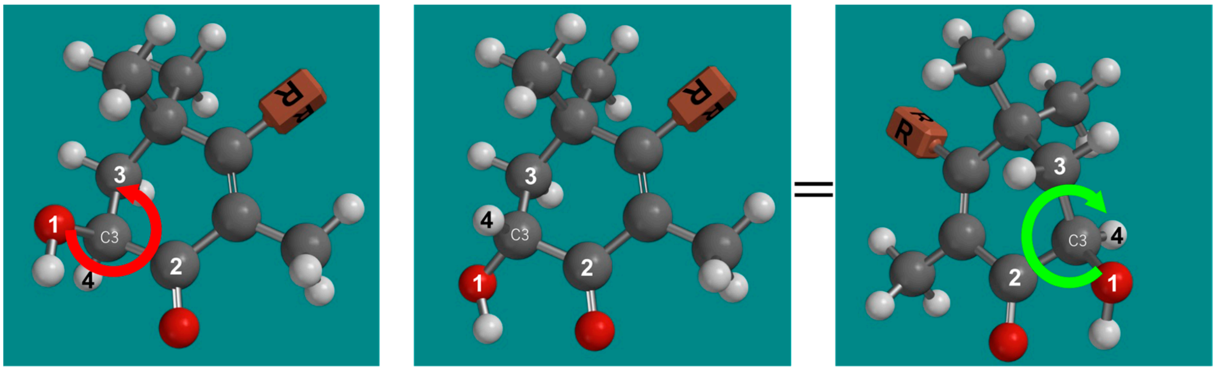

S-configuration

R-configuration

Figure X3. Schematic illustration to explain the assignment of the *S*- and *R*-configurations of astaxanthin (AX). 3D model of the molecule of one terminal end of AX was constructed using a SPARTAN '20 program ver. 1.1.2 (Wavefunction Inc.). Atoms drawn with white, dark grey, and red colors show, respectively, hydrogen, carbon, and oxygen atoms. The fragment R shows the rest of the AX molecule. The asymmetric carbon atoms of the positions 3 are denoted with C3 for clarity. According to the sequence rule, the priority of four atoms attached to the C3 carbon is denoted as **1**, **2**, **3**, and **4**.
